# Benchmark data for identifying N^6^-methyladenosine sites in the *Saccharomyces cerevisiae* genome

**DOI:** 10.1016/j.dib.2015.09.008

**Published:** 2015-09-30

**Authors:** Wei Chen, Pengmian Feng, Hui Ding, Hao Lin, Kuo-Chen Chou

**Affiliations:** a Department of Physics, School of Sciences, Center for Genomics and Computational Biology, North China University of Science and Technology, Tangshan 063009, China; b School of Public Health, North China University of Science and Technology, Tangshan 063000, China; c Key Laboratory for Neuro-Information of Ministry of Education, Center of Bioinformatics and Center for Information in Biomedicine, School of Life Science and Technology, University of Electronic Science and Technology of China, Chengdu 610054, China; d Gordon Life Science Institute, Belmont, MA, United States; e Center of Excellence in Genomic Medicine Research (CEGMR), King Abdulaziz University, Jeddah 21589, Saudi Arabia

**Keywords:** N6-methyladenosine sites, PseKNC, PseAAC

## Abstract

This data article contains the benchmark dataset for training and testing iRNA-Methyl, a web-server predictor for identifying N^6^-methyladenosine sites in RNA (Chen et al., 2015 [15]). It can also be used to develop other predictors for identifying N^6^-methyladenosine sites in the *Saccharomyces cerevisiae* genome.

**Specifications table**

**Table:** 

Subject area	Biology
More specific subject area	Bioinformatics, computational biology, biomedicine
Type of data	Text file
How data was acquired	Using flexible sliding window approach
Data format	Analyzed
Experimental factors	N/A
Experimental features	RNA sample was formulated by combining its dinucleotide composition (DNC) [1], [2] and the pseudo components [3] since nearly all the machine-learning algorithms can only handle vectors [4]. The concept of pseudo components was originally introduced to reflect the sequence patterns of protein sequences via a series of vector components [5], [6] and has been widely used in computational proteomics [7]. Recently, it has been successfully extended to cover DNA [8], [9], [10], [11] and RNA sequences [12], [13] as well. For the detailed development process in this regard, see a recent review particle [14].
Data source location	Chengdu 610054, China
Data accessibility	In Appendix A of this paper and at the web-site http://lin.uestc.edu.cn/server/iRNAMethy/data

**Value of the data**

• N6-methyladenosine (m^6^A) is one of the most abundant RNA methylations and plays very important roles in many biological processes [15].
• For in-depth understanding the regulatory mechanism of m^6^A, it is indispensable to characterize its sites in a genome-wide scope.
• The data can be used to develop computational predictors or high throughput tools for identifying the m^6^A sites in RNA.

## 1 Background

The benchmark dataset for developing computational methods to identify the methylation sites in DNA (see, e.g., [16]) is available [17], and the information thus obtained is very useful for both basic research and drug development. But so far no existing benchmark dataset whatsoever is available for developing computational methods to identify N6-methyladenosine in RNA. The present study was initiated in an attempt to construct a benchmark dataset for the later based on the experimental observations reported by Schwartz et al. [18] recently.

## 2 Data, experimental design, materials and methods

The data presented here are the benchmark dataset for training and testing iRNA-Methyl [15] (http://lin.uestc.edu.cn/server/iRNA-Methyl), a web-server predictor for identifying m^6^A sites in the *S. cerevisiae* genome. By means of the m^6^A-seq technique, Schwartz et al. [18] first identified 1,307 methylated adenine (m^6^A) sites in the *S. cerevisiae* genome. They have observed that most of the m^6^A sites share a consensus motif GAC where its center base may be methylated [18]. To construct the corresponding negative benchmark dataset, we used the flexible sliding window approach [19], [20] to search the *S. cerevisiae* genome, and obtained 33,280 RNA segments with exactly the same GAC consensus motif that, however, were not detected by the m^6^A-seq technique as methylated sites. Furthermore, it had been observed via preliminary tests that when the length of the RNA segments thus derived was 51 bp, the corresponding outcomes were most promising [15]. Accordingly, the 1,307 and 33,280 RNA segments each having 51 bp long were designated as positive and negative samples, respectively. Also, since the size of the negative samples thus obtained is overwhelmingly larger than that of the positive samples, to minimize the false prediction caused by such a highly skewed benchmark dataset, we randomly picked out 1,307 RNA segments from the 33,280 negative samples to form a negative subset that has the same size with the positive one. The final benchmark dataset thus obtained contains 1,307 positive samples and 1,307 negative samples. Their detailed sequences are given in Appendix A. They can also be downloaded at the web-site http://lin.uestc.edu.cn/server/iRNAMethyl/data.

## Conflict of interest

None of the authors claims conflicting interest.
