# Supplementary material for: Benchmark data for identifying N6-methyladenosine sites in the Saccharomyces cerevisiae genome
Source: Data Brief. 2015 Sep 30;5:376–8. doi: 10.1016/j.dib.2015.09.008 (PMC4773366; doi:10.1016/j.dib.2015.09.008)
Supplement: Supplementary file 1 — Supplementary material [file mmc1.doc]

**Appendix A. The sequences of the 1,307 positive RNA samples and 1,307 negative RNA samples.** Each sample is a 51-bp long RNA segment with a consensus motif GAC at the center. See the main text for further explanation.

**I. List of 1,307 RNA samples in the positive subset**

>P1

CAAAGGUGACCCACUUCGUUCAUGGACGUUCCCUGAAAUCAGGGACACUAU

>P2

GAUUGACAAUGACGAAGCAGAAGGGACUACAGAAUUAUUGCUGCUUGCCAU

>P3

AAUAGAGCUGACAACCAUUUCAAGGACACUUAUGGUUUCUCUAUUUUAGAC

>P4

UACCUUUGAACACAUUGCGCUAUGGACAAUUUUACAAUUGCUAGAAAGUCA

>P5

ACCGCGGCCACGCUAUUUAAAAGAGACAUUGUCACAAUGAACUUUAACAAU

>P6

AGGGUCAUUGUACCGCGAACUUUGGACCAUACAAGAAGAUCUGGAUCAUUU

>P7

ACAAUUUUAAGGGAAAUGCUAGAGGACAUUCAAUAGCUUCAUCAUUUGUUA

>P8

GCUUGCCGUAGCACAUGACAUACGGACUAUUGUGUGAGUGGUGAUGGGGUG

>P9

UUACUGGCUGACACUAGCUCUAUGGACCUCUCAGGCACGGGCGGCCAACAA

>P10

UCGCCCCAGACCCCAGCGUAUCUGGACUUUGAAGAACUUUGUAUCAAGGCA

>P11

ACGGUCCAUAGACGGAUCUAAUGGGACAUUUCAUGCUGCAGAGGAUAUUGC

>P12

AAGUAUGUGUUGAAAGGACAUGAAGACAUUAGACAGGAUAGCUUGGUCAUG

>P13

GGCAAUUACAUAAAGCCCUCUCAGGACAACGUGGAUAGCAAGGACUAACCG

>P14

CCACAAGAGCUUCUGUUGAUAAUGGACUAUGUGAUAAUUCUCUACAAAUCG

>P15

AAAUUGACUUUACUGAUUAUUUAGGACCAUAACACCAUUACAAAGACAAAG

>P16

AAAAAACAUGAACGAUCUCCCUCAGACUUUAUUAUAGACUAUAGCGUGUAC

>P17

AUGUGGACAGCAUUUACACCAAUGGACAUUUUGGGAGGAUUCUGGUUAUAA

>P18

AAGGCUACUGCCAAGACAUUCACGGACAAUAAUAGGCAGAAUGUUGGCUUU

>P19

GUUUUCAUACUGGGGCCGCCGGAGGACAAGAACAGUAAUAGUAACAUUAAC

>P20

GUGAAACAACUCGACGAUAUAACAGACAGUAAUACAACAGAAAUUUUAACU

>P21

UUAGGAGAAAUGGCCAAAUGCCUAGACAAUCCAGACCAGGGAAUAAGUGAU

>P22

ACUUUGGCGGAUCUUAUACCAGAGGACAUUUGACUACCAUGAACACUUUGA

>P23

UGGUCUAGUAGCUAUUUGGAUUCGGACAAUAAUCGGCGAAGGGUAUCUUCA

>P24

UUCAGCGAGGACAAUUUCAAUAAGGACUAUCAGUAUAGCGAAACGAGAAAA

>P25

AACUUCAUUAAGACAAUAUUUAAGGACUUUGUGUAAAGAUGCAGAGGUUUC

>P26

UGGUCAUACGACGGUAAAACAAUGGACAAUUGUAUCGCUUUAAGGACUAUG

>P27

UACAAAUCGGACAGGAAAUUUAGGGACAUUAUUUCCUACAACGAUAUCAUU

>P28

UGUCUACGAACACGAACACGAAAAGACUUUCAUUUAAGCUCUCCAUAUCUA

>P29

AUCAAGAGCAUGGACAUGGACACGGACAUUCCCAUGGCGGUAUCUUUGCCG

>P30

GGAAAAGGGGGAGUGAUAGAUUUGGACAAUAGUGUUACCCAGGAAGUAUAA

>P31

UCUGCUGUUGAUAUAUUGCAAAUGGACGAUAAUAAUAACAGUCUACCAACU

>P32

AAAAAUGAAAACACGGAUUUGAAAGACAUUAUCCAAGGUAAUCCACUGAUG

>P33

CAAAGCUACAUUUCCAACAGAGAGGACUUCAAUCAUGACAAUUUCAUUAAU

>P34

GUAUUGUAUACACUUUAACUAAUAGACGAUAUGGUGAAAAAGUGGUUGCUU

>P35

GGACAAUUACCUGAGGAUGCUAAGGACAUUAUUGCGAAAAGCGCCUCUAAU

>P36

UAUUACAGCAGCUGAAGGACAAUGGACAUAGGGCACUUAUCUUCACACAAA

>P37

UCAAAUCAGGUUGAACAUGCAAAGGACAAUGCAAAAAGAUGUUUCUGUUUU

>P38

UCGGGCAAAGACUUCACGUACAUGGACUAUUUAGAAAAGACACCCGAAUGG

>P39

AAGGAACUGCUUCUUGAAAUUGUGGACAAUGAAAUGAGCAAAUUCGCAGAG

>P40

UUCAAUCCAAGCAGGUUCCCCAUGGACGCUAUGUCGGCUGCCACAAACGAC

>P41

GUUCGUAGAGAGCGAAUAUGUUUGGACACUUAAGGAACAUACUAUGUUUAU

>P42

AGAUCACGAAAAGGACACUUAUCGGACAUUAAUGGAGGAAAUUGAAUCACU

>P43

GCGAACAACGUUUUUGGCUCCAGGGACUAAAUGGAUUCAAGACUGGAAAGA

>P44

UUAUUUUGUAUGGUACAUUAGCAAGACAUUUACAGCAAAGUGAUGCAAGGA

>P45

ACUCUGCAGGAGGUUAAUAACGCGGACGAUUAUUUUUCAUUGGGAGUGAAU

>P46

UGCUGGAACAGCAUAUACCUAAUAGACUUUCGGCAGAGAGCAGACCAACUU

>P47

UGGAUUCACUUGCCAAUCAUUAAGGACUUAAAGAAAGCCUGUUCUGAAGUU

>P48

AUCCAUCCACCCGUAUUCAGAUUGGACAUUCGUUUAGCAAAAGAUUUAAAA

>P49

CAAAUUUCUACUACGACAUCCAUGGACGUAACAACAAACGCAAACGUGAAC

>P50

CCCUUCCAAAGCGAAGAAACUGAGGACAAUUCUGCCUUUGCAUUUGAAGAU

>P51

GACUUGGGUCUCCAGGAAAUCAAGGACAUUGCAGAUGAAAUGAAGGGCAAA

>P52

UGGAGAACGAUUUAGAAGUAAAUGGACUAUUUUACGCAAUAGGUCACAGCC

>P53

CCAAGAUUUUUGCGUAUAAGAGAAGACAAGGGCGUAGAAGAUGCAACCUCU

>P54

GCCAAAUUUACCAACGUUAUUAAGGACAUUUACCGUGAAAAUAAUGGGGAC

>P55

AUUGGCGAUGUCGAUACACAAGAAGACGGCGUUAUUGAAACGAAUAUAAUC

>P56

GAAGGCCAAAUCAAGGCGGGAAGGGACAACCAGGACGUAAAGGGUAGCCUC

>P57

GUAAGCAGCAAUAGUGUUAACAAGGACAGUAACAAUAGCUCUGCAACACCC

>P58

ACAAAUCUAACUCCCUGGGAAAUAGACAUUUAGCCAGUGUUGAGAAACUUG

>P59

AUUACUUGAAAGAACUACGAAACGGACACAGUCUCGGCUCAGUCUUACACC

>P60

UUUAACUACCACUUUCGCAAGAGAGACGGAGGGGGUGGGAAAAGGCUGAAU

>P61

UACAGCACGGAAGAUAAAAAAUUGGACAAGAUUGCCUUUUUCGCGGGACUU

>P62

GGGGUUCUAUGUCAACCUCGCAAAGACAGUUUAAUGGAUUGCUAGACGUGU

>P63

GAGAUCGCGGCAAAGGAAAUUAUGGACGAUUGGUUGGCUUAUGAAAAUGUC

>P64

UAACGCUGAGGAGUUAAAGACCAAGACCGAAGAAUUGCAAACUUCCUCGAU

>P65

UCGUUACGUUCAAGGGACAUUAGGGACGUCGCAUUGAAAAGAGAGUUUACU

>P66

CUUUAUCUGGAAGAAGGUUCCGGAGACCAAGAGGAGCGGCUAAAAUGAAAU

>P67

CGAUGCUCUUGAUGCCUCUAACGGGACAAUAGACUUGUAUUUAAGAGUGGC

>P68

AGGACAACAGGGCAGAAAAAAGUUGACAUUAUGCAAUUGAUGAAAGAAGAA

>P69

ACUAUGAGGUUGAUGAUGCAGAUGGACUAAUUAUCAAUAUUGACCAGCCCU

>P70

GCAUGCUCCGAUAAUUGCGACAGGGACUAAACAAAUAAAGAUAUGGACGAC

>P71

ACUACGGGUUUAGAAGGAAAAAUGGACUUUUCAAGCAGCCUUCCGUAUUGC

>P72

CACCGCGGCCUGUAUCAUCGUUGGGACAGUUUACAUUUUUGUUAAAGAGAU

>P73

AUGAUGACAUCUGGUCCGGUAGAAGACUUUUGGACGAUUAAUGAUGACUAC

>P74

AUAGUGAUAAUAUGCCAUCUAACGGACUUUUUGGCAAUAGUACAUCGGACA

>P75

GUGGAUGAUGACACUGUAGACAAGGACUCUAAAUGUGUCACUUUUGCAGCA

>P76

AACUAAUGGGGAAGAAAUGAAUUGGACGUUUAUGAAUGCCUUUGAGGCUGG

>P77

CGUAGCAAUCGGUGAAAUUUCAAGGACAUUGACUGAAACUGUGGGGCCAAA

>P78

GAAAGCGAUGAAAAUACUAACCAGGACUUUUUGGAAAAAGCUGAUAUGAGU

>P79

GAUAGUGGUUACCAGUCGAAUAUGGACAAUAUAUCUUCUCAUAGGGUAAAU

>P80

AAUGCGUUGUAGGGACGCGUUAAGGACAUUCCUCUUUAUUCAAGUAUUUAU

>P81

GUGCUUACGAGCAUGGGCUGGGUGGACUUUUACAAAGCACAACAUAUUUAU

>P82

UGCUUUCGUUACUACAGCACACAAGACUAUUGUACUUUGUUAAAGAACUAU

>P83

CCAAAACGCGGAUGGCUUACCAAAGACAUUUAGAAAUAGAGCAUUUUCCAU

>P84

GCUUCAUUGAACAGCAUUACUAAGGACAAUAUCGAUUAUGCACCUAUGAGA

>P85

CUUCCUUCGAAACGCGACUAUCCGGACUAUUUUAAAGUAAUUGAAAAACCC

>P86

GGCUGUCGAUGACGACAACUUGCAGACGGAAGAAAAUUCCGCAGACGUUAA

>P87

AUGAGGAACAAAUAGCACAACAGAGACAUUCAGCGGAAGGUUUCCAAGGGA

>P88

AGAAUUGCUAUUGUCAAGUAUAAAGACGGAUUUCUGAUCAAUGGGCACAAC

>P89

UCUUAUAGUUACAGAAGCGAAAUGGACGUUGAUACUACUGACGAUUAUAUU

>P90

AUUAGACGUUUAGCUAAUUCUUUGGACUUUCCUGACUCUGUUAUGGACUUU

>P91

AUAAUUAUGCCAAGGGGUAUGCGAGACUACAAGACUACAUGGACAAAUAUA

>P92

GUGGGGACAUUUGGAAGAGAAACGGACUUCUCAAAUAUUGUUUUAAUUAUC

>P93

GGCAAAAUUAAAAUAGACGGUACGGACGUAAAUGACUGGAAUUUGACAAGU

>P94

UAUAUUGAUGAGUAUUCCCAUCUGGACGUUUUAUGGGCGCAUGAUGUUAUA

>P95

AUUUGAUAGCCUUCUUAAGAAAUGGACCAUUAUAAAGUUUUUGUAUCGCGA

>P96

GUGAUGUUAUCAAGAUUGCUCAAAGACUUUAGAUCAGUAAACAGACAUAUA

>P97

UAAAAAAAGGUUUGCUGCAAUUUGGACUUUAUGUAAAACAAAAAUGGUCUG

>P98

CAGGUUACUAAGGAAUAUUUCCAGGACGUUUAUGAUUUGACUGGCUCCGAA

>P99

AACGAAUUGGCAUAGACACCUAUGGACUAGUAAACAAAAAAUACUCAAAUC

>P100

CUACUGGAUUUGGGUUUGGUAAUGGACCUCAACAAUCAAGGCAAGCCAACA

>P101

AAUGCAAAGACCGGAGAUUUGUGGGACGCCUUAGCAGAUGCCUCUGGCAAG

>P102

CGGGUCGGUAAAAGCGGUAUUAAGGACUUGAAGAAGCCUACGAACCAGAAG

>P103

UCAAUGGAUAAUAGUAAGAAAUAAGACGGAAACUCCUUUUUGUGACUUUCU

>P104

CGCGAUACGUAUUUUGGAAAACUGGACUUUUCCCUCAAAUGCAUCUGGUAA

>P105

UUCAAGAAUGACCUCGUUUCUAUAGACUCUAAAAACCAUAAUGUUAUCCUG

>P106

GAAAUGAAGCUUUUGAAGAAAAAGGACGAUCCUUCUAAAGUGCGCCUACUU

>P107

CGGGCUUGAAUUUGUAGAACCAUGGACAGAACUUAUUGAGCAACGAUAUAU

>P108

UUGAACCAUACAGCAAACCUAAUAGACAUUAUAACUUAACCACCGCUGGAA

>P109

UAUCUGACAUUAAAGUUAAUACUGGACAUUGAAAAUUCUCAUGUAGGAGAU

>P110

AAUUCAAGAAAAACCAUGGCUAAGGACAAUAAGCCCAUGCCAGCUGAUGCG

>P111

UGAGAAAUCACCACUGGAAACUAAGACAGUAGAUGCUCAAACGAGAAGGGA

>P112

UGUUUUGACACGGCAGAGGAGCUAGACGAUAUGAACAUUGAAAUUAUUAGA

>P113

CCAUCUACGAUAAGGGUGCCGCAAGACCAACCGGUGGUGCCGGUACUGUUG

>P114

AUUUUGCCACAUAGUGGUUCACAGGACGAUUUAAGAAAACAAUUAGGCUCU

>P115

UUAAAAAAUGCGGCAAAGGAUAAGGACAAUCCAAGAAAUUCUCAUACCAUC

>P116

UUCCCAUUAAGCGGUAAAGUUACAGACUUUUUAUCUCAACAUCAAAAUAGC

>P117

GAGAGCUCCAAUGAUGAAAACAAGGACGAUGACUUAGAAGUUUUAUCAGAA

>P118

CCUAGUUUCAAGCCGAUCUACAAAGACACUAGGUUUGGAGGACGCAUGGAU

>P119

AGUGACAAUUUGAAAUACUCCUUGGACUGUGACUUUUCAUGAACGCUUUAA

>P120

GUAGCAGAAAGAGUCUCCCGAAAAGACAUACUGCAGAACGGCCGCUUAUUC

>P121

CACCGGUUUAGGAUAUAUGGCAUGGACUCAACCGACAAGGGAAUUGACUGC

>P122

GUUGUUAGAUGCCAACAAUUCGCGGACAUUGCAACAAGAAGCGUGGCAACA

>P123

CAUAUGAGUGAACCGGAAUAUGAGGACAUGAAAUUUCCAGAUAAGUUUUUA

>P124

ACUAUAUUGCUUACUGAAAAUCAAGACUCAAUAAUUUUUUUCUCUUCAUGU

>P125

UCAUAAGGCAUUUGGGACAUUAAAGACAAAUGACGAGCCUUUCCAGUGUCG

>P126

CUGAUUCUUCAAAAGAGCAUAACGGACGGUGUAAUGCGAAAGGUCAUUGGU

>P127

UCGUUUUCACUCCAGUACUAUACAGACAUACGUUUUAUGCAUCCAUUAGUC

>P128

AAUGAAGUGAUUGAACUUUUGAAGGACUCCGAGGAAAGAGCAAGAAGAUUA

>P129

AGGAGUGGUGUCAUUGAAACAGUAGACGAUAAAUUAAAAGAUAUAGUGGGC

>P130

ACUGUAGAAGAGGCAAGUUUGAAGGACGAAAUAAACUAUUUGAAUUCGAAG

>P131

UUAUGUGCUCAGACUACGCCAUUGGACAAUUCAGAUAUUUGGCUAGAUUGG

>P132

CAAGCAGGUGUGCAAGACACUAGAGACUCCUAACAUGAUGUAUGCCAAUAA

>P133

GGUGGUGAAACGGAGGAGGACGCGGACGCUUUAAACUCUGAUCACUUCACC

>P134

GAUCAUGCGAUUCAGUUUGUAGUAGACAAUUCCUUUACCAGAACUUCAUCU

>P135

ACUCAUCGUGAUAAGAAUAAAAUGGACAUGAUUUGCAGAAAUAUCAACAGA

>P136

UCCAGCACGCCAACGCCGCCACCAGACGCAAGUAGUGGAGUAAUGGCAAUG

>P137

CCAAUCAUUGCUUUUGGUACCAGAGACUCUCUAUUCCCUAAAGUAGUUUCC

>P138

GAGAAAUCUUGGGAGGAUGAUAUGGACGAUAUGGACGAUGAUGAUGAUGAU

>P139

CCAAAGGAUGCUUGUAGAUUAAAGGACUUCAAAGCCAAGGUCGACGAAGGC

>P140

AAUAGGAAAGAUAAACUCUCUAAGGACAUAUUAGAUGAUCUUUCAUCACAA

>P141

UUGAAGGAUUAAUGAUCAUUCUAGGACAUUAGUAAUUGGAAAAGAAAAUGC

>P142

AGUAAAAAAACCGAAUCGUAUAUGGACAAUAUAAGGAAACUAUCUUCUCAG

>P143

AAUUUGGGCAUCAUUGCGGACGCAGACUUUGUACCGAACCCUUUCAGGGUU

>P144

AGGAGAACACCUGAAGAUUGUAAGGACGACAAGUAUUUUGUUAAUUGAAUU

>P145

GAAGACGAAAGGGAUUAUAUUUUGGACUUUGAAUUUAACUACAGAAUUGGA

>P146

CUGUACCAUUAUGUAAUAUAAUAAGACACGGACGCACACACAAAAGAAUAG

>P147

UAACACAAUCAUGGGGGAAAAAAGGACAAUAAUCAAAGCGCUGAAAAACUC

>P148

CUUAUACCAUCAUAGUUAUAAAUGGACAUUUAUCACACUUCUCAUCACUUC

>P149

GAUGUAAAUAUUACCGAACUAGAGGACGAUGACGUUUUGAAAGGUGGCGAA

>P150

CACCUUACGAUAUACCUGAAGAAGGACUUUCUCAAAAAAUAUGCAUCUUAA

>P151

GUUGAUACGCAAUCGAAGAUUUUGGACAUUGAAGAAAAAAUGGAAAAUUUA

>P152

AAAAACAUGAAGAUCGAAGAUGAGGACUUUUUAAAGCUAAUGAAGAAGAUU

>P153

CUAAUUCAGACAAUAAUUAACGAGGACACUGGUUUUCACGAUGAUAUUACG

>P154

CUUCCGAACGCCUAUUUUGCUCAGGACAGUGAAGAAGAAUUAAUGUCGCCA

>P155

AAAAUUGUUGGUCUAGCGGUUAUGGACGUGGCGCUUGCUAAGGAAUUCUUA

>P156

GAUGCAUCCUCUUCAAUAACAAAGGACAUGAAAGGAUUUAAAGUAGUAGAA

>P157

ACUUAUUGGACGAAGCACUACUAAGACCAGGACGAUUCGAUAAAUUGUUAU

>P158

GACGUGUUUCGACAGCAAGUAAAGGACAAGGAAAAUUUGCAUAAGUCGGAA

>P159

UCAUUUAUCUCAACGUUAUUAAAGGACUUACAACGCUAUGUGAAUUAGAGC

>P160

AUUAAUCCGGCCAUAUGGACUAGGGACAAUAUUGCAAAAAUGAAACAACAA

>P161

AGCACGGGGUUGCAGGCGUAAUAGGACUAAUUUUCAAUGCCCUUUUUGGAG

>P162

AUGAACUUUUUGAAUGAUUUGAAGGACAAGCUAAUUCCGCUGGGCAGAAAG

>P163

GACAAUUCAUUUGAUAGUGUCGCGGACGAUUGGGACGAUAUAUUGAGAAAU

>P164

AAGCUUUAUCCGGAACACUUUAAGGACUUCAAAAACAACACUACCUGCGCU

>P165

UGAUGUUAUCCAGGGGAUCUCACGGACGUUGGCUUCUUUUAAUGAUAUCCU

>P166

GUAGGAUAGCCAAAUUUUUUAAUAGACGUUAAAAAAUUAGAUAUUAUCUCA

>P167

AUAUGUUUGCUCGUGUUGAUAAGGGACUAUCUGAGGCAAUUAAAAAAGUAG

>P168

CACAUGCCGCUAGCAUCUAUACCGGACAUUUUCAUUACGCCUACUCCCGUU

>P169

AAUUACGUUUUACAGACAGCAUUAGACAUUUCUCAUAAGCAAAAUGACUAU

>P170

UGUUUGCCACUUGAAUUUACAUAAGACAUUUUCCAUUCCUCAUGGUGGUGG

>P171

GGUUUGGAUUUUGUCUACAAAUUGGACGAGUUUAUCAAAAAGAACAGUGAU

>P172

GAACAUUUUGCUGAUCAGUUUAAGGACAAGUACAAGAUUGACAUUAGGAAA

>P173

UUAGAAAUGGAGCAUACGAGCAAGGACAUUCUCAUCAUUGCACAUGAGUCA

>P174

UUACAGGAACAUGGACUAUAUAGGGACAAUUCGGAUUACAGGGCAGAGGGU

>P175

GACAGGCUUAAGAUCUUGUAGAAGGACACAUAAACAAUAUUUUUGGAAAAA

>P176

AAAAUUAUGAGCCAAGAUAGCGAGGACGAUUCAAUACGGGAUGACUCAAAU

>P177

GCGUACGAAUCUGGGUGGUAUGAAGACGAAGAUUCUGGUGAUGACGAUAUG

>P178

UUAAGAUGGAUCGGUAAACUGCUAGACAAACCUGACAUAUUUUUGGAAAAG

>P179

UGACAACUCCGGCACUGUCCUAGGGACUGCCUAGACAUUUGUAUCGCUAUU

>P180

GGAAUGUUAAAACAAAAGGAAAAAGACAUUACAGCACCAAAACAGCCCUAU

>P181

CUUCUUAAAGCAGUGCAGGGACGAGACUGUCGCCACUGCGGACAUUAAAAA

>P182

UUUUACAACCAAAUAAUGAUGCUAGACCCACAGCGAAGACAGUGGAGGAGA

>P183

GCUCCAUUAGGUACUACGGCUACGGACAAUGCUAACAGUAAUAAUAGUGCU

>P184

GUGACAUAGCUCAUCCAUACAAGAGACGAUGUUCAUUCAGGGUAAGGAGAG

>P185

AUGAGAAAGAUCUCAAGCGAAACGGACGAUGACCACUCACAAGUGAUUAAU

>P186

AUGAUGGUGUUGCAGCCGUCAAGGGACUAAUGAAGCCAUCAGGCGGUAAGU

>P187

AACAUUAAGCUGGGUGUACAUGAGGACACUCAAAACAGAGCUGCUUUAGCU

>P188

GCCAGAACGUUACUAUAUCACAUGGACCUGCUUUUGAAAGUUAAAUGAAUG

>P189

UCAGUGGCGAACAGCUCUGAAAUAGACAGUGAAAAAAUGCAUUGGACAGAG

>P190

AAAUUGGUCCUAAUUCUACAAAGAGACAGUCAAAUGCUCCAAGUUUAAGCA

>P191

GUUGCAGCAGCAGAAGGGUUUACGGACAUUGUAAAGCUCUUGAUAGCCAAC

>P192

AUAAUGUCAAAAAGGAUUGCAAGAGACAAUAACCUUGGUCAUCACCUAUUU

>P193

GAUGGGGCCGAUCAAGGUAAGGAGGACAUUCCUGAUAGUAUAUUAGAGCUG

>P194

CACUUUUUCUGUAUACGAUAAACGGACUUAAAAGAAAGAAAAGCCUACAAU

>P195

UACCUGACUCCAUGAUGAAUUCUGGACGUUACAUUGCUGCGCAAACUGUCC

>P196

UGACCUACCAGAAACUGCCGGAAAGACUUUCGUGGAAAUAAAUGAGUUAUU

>P197

AGAAGGGCUGGCUGAGAGGCAAAAGACUUUGGUUUUUAGUGUUGACCAACU

>P198

AACAAUUGAACAAGAAAAGCAAUGGACAGAAUAUUUUGCCUUAUAUUGAAU

>P199

UAGCUAAUGUACUGGAGCAAGAAAGACACAGGUCAACAAUUACGGAGCCAU

>P200

GAACUAAAUUUUGACGCCGUGAAGGACUUUAAGUUGGGAAAAUUCAACUAU

>P201

AAAAACGGAUGUUUAGGCGCCAUGGACAAUAUCACACGUGAAAAUUGGUUA

>P202

UAAAACUACUACUGUCAAUGACAAGACCACCGAGUCGAAUCCAAGUUCUGA

>P203

AUCAAGGAAUCUUCUGCAAAGAAAGACGAUGGUGAAGAAUUCGAGGAAGAU

>P204

ACGGCUCAAUCAAACAAUUUUAUGGACAAGACUGGGUUAUUGAUUACAUUA

>P205

AAAAUUCAAGAAAAACUUCAAAAAGACGAUUUCAUUAAAGUCUCGACUGCC

>P206

UAGGAUAAGUACUCCAUCUGAGGAGACACUAACGAACAGAAAAAUUGACGA

>P207

CUUAGCUAUUGGCAGUAAACAUUGGACAUCAUUUAUUUUACCUCAAUUUUU

>P208

UAAUACCGCAUUCGCAUGUGUUUGGACCGAACUUUAUAGCCAAUAUCAAGA

>P209

GGCCGAUGAAAUGGGGCUGGGUAAGACAUUGCAGUGUAUAGCACUUAUGUG

>P210

UUCUUUUCGUAAAAAAAAACAAUAGACACUAUAUAUAGACACUUUUUCCUU

>P211

GGAAUCUCAUGGAUUUCUUACACGGACUCUUGCAACUUAAUCCGGAUAUGA

>P212

UUAGAUCUAUUGAAGGAUAAUAUGGACUCUAAACCGGAACAAUUAGAACCC

>P213

AUGGUAUUCCUGACGGUGUUAAUGGACAGUACUUGAGCUAUAAUGACCCUG

>P214

CGAACAGUAUUGAUUUUAAUUAUAGACCACCGAACCCACCCGCUAAUAAUC

>P215

AUAUCUGGAAGUGUAUUGACAAUGGACGAUAUCGACAUUAAUGACACAUCC

>P216

GGGGCAUUGCAUGGUAUUGCACAGGACUCUACACCUUGGAUCUUUAACGAG

>P217

GCCAAAAAUAAUUUUAAGAUAAUAGACAGUCAUAGCAUGAACAAUUCUGGA

>P218

AAACAAAAAAAUGUAGUUGAGAAAGACAUUGAAAAUUCUGAGAUUGCUGAU

>P219

GGGGAUAAAACGGCUGUUAAGCUGGACACUGAAGUAAAGAACACAAAUUCU

>P220

ACUUCGGCGAGCAUAAGCUAAAGGGACUCGAAACCAAAGAACUCGUUACUA

>P221

CAAUCACUGAAAAUACUGGGUAUGGACAUUUUCACUGCUCACAGCAUAGGA

>P222

CGCUCGAUUUGAUUCAUGAAGAAGGACAAAAGAACUAUUUAAUGUUCAUGA

>P223

GAAAGAUCCAAAGGUUGGCUGAAGGACAUAGAAGGUGAACACGAACAGGUU

>P224

CAAGCCAUCUGAAGAGGAUUUAUGGACUAUCAAAUUAAGGUGGAAAUAUGA

>P225

AAUUAUAACAAGCAUUUGCUUAGGGACUCUUUGCACGAGACGUAUAUCAGA

>P226

ACGGAUGGUGAUGGAAGUAACAAGGACAAUAUAUCAUUAAGUGCAUUAAUU

>P227

CUUGCCAGCAUAGAUGAAAAUACGGACACUUACUUGGUUGCUGGGUUAACA

>P228

UGGAUAAGUGAUCCAACUGAUACGGACUUACUGAACCUGUUACCUUUCUUG

>P229

ACCAAGAUUGUUAAUAAACCUAAGGACGUUCUUAUUGUUAUUGAUCAAUUG

>P230

ACGGUGGUUACCAACAACAACAGGGACAAUCUGGUGGUGCUUUUUCCUCAU

>P231

ACAAAACUCUCAAAGAAUCACAUGGACUAGCGAAAGAGUUGACCAAGAGUU

>P232

AAAAUUAGAGAAGAAAAAAUUACAGACUUUCAAAACAAAAACGCUAGCAAG

>P233

AGACCCCCUAAGGACUGGAACAAAGACAUUAAGCAAUUCGAGAACUCUCGA

>P234

GUGGUCGAUAAAUUGUAUGAAAUGGACAAUGCAGUCAAUAAUCCUCCGAAA

>P235

AUACUUAUGAAUUGGUCUAGCAAAGACGUUGUUUCAUCGCGCUAUUACCAA

>P236

AAAAGACGUGGACUAGAGAGAAAGGACAUUCCUGCAUGGAAACUUUGUAUU

>P237

CCAAUUGUAGAGACUAUCCACAAGGACAAUAUUUGUGACUUAUGUUAUGCG

>P238

GCGUACUGUCAGAAAGUUCUUAAGGACAAUAGGCUGCUGCCAUCGUGGCCC

>P239

UCUCAAGAUGCAAUAGCAACAAUGGACGUUGUUAAAGUCAAAAUAGGUAUU

>P240

ACCAGUGAGAAAAAUGACAUGUUGGACAUUAAGUAGGUUUUCACCAUGGAU

>P241

UCAAUGUGUGAGAUUAUGCAAAAGGACAAUAUGCAGCCUUGUUUCGAUGCU

>P242

UUUUGGGGGAGGACUAUAAAUAAGGACGUUCAAGUCAGCUUCAAUGACAAG

>P243

UGUUCGCCACUCCUGAUACUUCUGGACAACACAUGAGAGGUCUUUCUGUUU

>P244

AAGGAGAAAACAUCAUUGGAAUUGGACAAUUUACCAUUAGAUGCGGCGACC

>P245

CAGGAAAUCUGUAAACAGUGUAUAGACUUUAGAUAGAUAUUUAGCAUAUCC

>P246

CAGUUUUUGCAAGAAAAUAAGUUGGACAAUACCUUGUUUUCACAGUCCCCA

>P247

CUGACAUUAUUAUUGUUGGAAGAGGACUAUUUGCAAAGGGAAGGGAUGCUA

>P248

ACAUUAACAAUGUUUAUGGCUGGAGACUACAACAGGAGCAUGAACUAAUAU

>P249

ACUUGAGAAAUUAAGUUUUUGACGGACAGCUGUGGAUGCUUUCAAGGGCAA

>P250

UACACUGAUUUCUUAAGAUGGCAGGACAAGGAUGCCCUAGAUUUGUCAGCA

>P251

UCCUAGUACGGAGACUAAAGGAAGGACGCACACAUCCACUGCUGCCGUGGU

>P252

CAUAUCGAGGAACUGGAUGCUUUAGACGAAGAACUUUAUAAGAGAAUGUCG

>P253

GAGGAUGAUUCACCAUCCCACACAGACGAUAUGAAGAACAAUUACAAUGAU

>P254

CAAUUAAAGAUUACCAAAUACUUGGACACAUUUCCUUUUGAAUUUUAUGUU

>P255

AUCCAUUGGACGAUACGUAGAAGAGACCGAACUAUCACAAAAAAAUUGAAC

>P256

AGCAUGGGCAAAUAAUAGCGCAAGGACUUUUUGUAGUGUCUGCAAAGAAAA

>P257

GUCAGAGAUAAAAGAGACGUAUAGGACAUAUACAAUAUUCAUACCUUUCAC

>P258

AGGCUUGAUUUAUCACAAAAAAUGGACUUUAUAUUGCCUUUUAGGUCUCCC

>P259

UCAAUGGGAAUGCCUUAUCGAUUGGACACUUUAGAUAUAAAAUGGGAGAAU

>P260

ACACUUAUCAUGCAACUGCUAAGGGACAAUUUAACCUUAUGGACUUCAGAC

>P261

GCCAUAUCUUCUGAAGACCCACAAGACAUUGACACUGACGAAAUGCAAGAU

>P262

CGAAGAAGGAGAAGGCAGUAUGAGGACUUAGAGAAUAGUGAUGACGAUCUA

>P263

CAAGAAGUCCCAUCAUUCUUGAAGGACGCUAUGAUGAGUGCUCCAGGUAGC

>P264

AUUGCUCCUUUACAGUACAAACGAGACAUUAAUGUGAACUUGGAAUUUAAU

>P265

UGAGCCCUUCAGCGAAGAGGUGAGGACUGUGAUAUUUGAAGGGUCGAAUCG

>P266

GAUUUUUAUCGAAACCAAUCAAAAGACCAAAAUUGAAAACUAUUCUUACUG

>P267

AGGAUUAAACCUGGUGCUAAAUGGGACGAUUUACAUGCACUGACACACAAG

>P268

AGGAAAUGCAUCGUCAAUUGAAGAGACAAAGCACUAGCUCAACUAAGGGUU

>P269

UACAAACAUAUCGAGCCUCUAAAGGACAGACUUAAUGAAGACGAUUAUUUA

>P270

AAUGAGAUGUUACAGAAGCAAAUGGACUUUGAACUUCUGGGUGAGAAUCAU

>P271

UAUUAUCCCAAAGCAAUUUUUAAAGACAAUUAAGAGAACGGGUUUGAAAAA

>P272

GUGGUCUUCAAAGGCACUUUGAGGGACUUUUUGGUGCAAAUCAAAGAAGUC

>P273

GAAUUAGUUGGUAAUCUAGUAGAAGACGAAGAGCUUUAUAAAGUUAUAAGC

>P274

UCUCACACGUACUACACCUCUCAGGACAUUAGAAGCGCGGUUUCUCAGUAU

>P275

UUAGCGGAUCUUGGAAAGCAACUGGACUUGCCGCGGAAUCCAAAAAUGCAU

>P276

GUCUUCAAAAAAUCGCUUCAAUUGGACUUAUUCCAUGGUUAGGAAUUUGCA

>P277

UCUAGGCAACCAAUAUUAUACAAAGACAUUUUAAGAUCGCUGAAAUUACUC

>P278

GAUUACAUGCUAUCCGAGUUAAGAGACAUUAUUAGCAGGGCAAAAAGCAAA

>P279

GGUGUACCAGCAACAGUGGUAAAGGACGAUUACGUACAACAGUUUAUUAAA

>P280

CAAAGAAAAACCGAUUCUCUAAUGGACGAUGGGGAAGAAGGUGAGCGUGAU

>P281

AUAAAUAAUGCUAGUAAGCUAAAGGACAAGAAAGUUGAUAGCGUGCAAACC

>P282

UACGACGUCAAUAGCUCCAUCGAGGACAAGCUUACCAGCAUCGUCCAAAAA

>P283

AGAAGCAAGAACAAAGCUAGCAAGGACUAUACUAAAGCCCACUAUUUAUUG

>P284

GGUGCGUUAAUAAAUGAUGGUCCGGACACUAAUUUAAACGCGAAUAACACC

>P285

GGUCACUUCGACACAUGCCACUAAGACAAUACAGGCUCAAACACAAGAUCC

>P286

CGUUGCUGGUGUUGUCACCAACUGGACAUUAGUUACCCAAGCUCACGGUAC

>P287

AUUUUACAAAAACAAAUCAUCGAGGACGUUGAGUGAUUUGUUGGCAUGAUG

>P288

UUAAGAGAUGCUAAGGAGACCUGGGACGCUCAAGUUAAGGAAGUUGAACAU

>P289

AAACAGAAAUAAACAUACUUACCAGACUGUGCGUAAACUUGACAUGGACUU

>P290

AAUAUCUAUCAUGCUGUCAAUAAGGACGUUGCCUCAUGGAUUGUGGAUUUC

>P291

AAAAACAAACAAAUUAGCGUAAGGGACUUAUUGCAUUACAAAUCUGCAAAU

>P292

GCUCAAAGAGUUGCUGAAAAGAGAGACGCUUUGCUAAAGGAAGACGCUUAA

>P293

ACAUUAGGUGAUGACGAUAAUGAGGACAGUAUGGAAAUUGAUGAUGACCUA

>P294

GUAUCCACUUUAGAAACUCUGAAGGACACUACGGAUAAACAUCACACUUUA

>P295

GGCUUUGCAAAUCAGCAACAUAUGGACGAGAGCAAUGUUUUGCCAGCAAAU

>P296

ACACUUGCCGCGUUAGAGACUUUAGACGCUGGAAAGCCUUAUCAUUCAAAU

>P297

CCCCCAAUGAGGGUCCUUUUAGUAGACGUUCUGAAUAUAUUGCUUACUUAA

>P298

AGGAUGAUGAGUGCAAUUUUGAAGGACAUGUUCAUACUACUUGGGGAGAUU

>P299

GCCUCCAUUUCUACGUUUGUUAUGGACAAAUUAGGGCAGACAAUGAGUGUU

>P300

AUGUACAAACGUUUAAAUAUGAAGGACGAUGACAAAUUAGAGUUCAAGUUU

>P301

UAUUGACACUCCAGCUUUGGAUAGGACUAUCAAGUGGCAAUUUACUCCGGG

>P302

CCGAUUACAUAUUCGUAGAGUUUGGACUUUAGAGUUGAGCGUUAUUGGGGU

>P303

GUUUCUAUGGAAACACUUUUAAAGGACAUAGCUACUUGGAAUACAUUCUAU

>P304

UCGGUAAGCGAUAUUCUAAAAAAGGACUACAAACAAUUCAAUUUCCAAGGA

>P305

UACGGUUGGAAAAGAAAUGUUAAAGACUUUUGGCUUACAAGCGAUAUAAAU

>P306

AUUCAACUAAUGAACAACGACACGGACAAUAAUAAAAUUAAUACUUCGCCC

>P307

AAUGAUAAAACCUUAGAACUGAUGGACAGUAAGAAGACCGGGCAAUUUAAC

>P308

GGACACAACAACCAUCACCGUCAGGACAAUAACAACAAUAACGGUGGAUUU

>P309

GUGCUGAUAUGUUGAACCGUGCCGGACACACGGUCACUGUUUAUGAAAGAU

>P310

AUCAAUGGUGGCAACGUAAAAGAGGACUAUAAGCCAAGAUUAAUUUUGUUU

>P311

AAUUCUUGGUUGAUAACCAAAAUAGACACUAUUUCAUUGAAAUUAAUCCAA

>P312

UCAUGCGUGGUCGAUGCGAAAGAAGACGUUGAAAGGUUGUUUAGUUCUGAC

>P313

UGUUGUUGGUGUUAGCUGUGUUAGGACUCUUUAAAAUGGCAAUCAAACCAA

>P314

UUGGAAUUUUGGCUUUACUUGGAAGACACGCUAUAGGCGCUAUCAUCUUUG

>P315

GAUGCUCAAAAGGCUUGUAGAAUAGACGUUAAUAAAGCAUCAAGGCUAUUU

>P316

ACAUUUUAGCAUGGUUACCGUACGGACUAUUUCAUUUUGGGGCCCCAUUUG

>P317

AGACUCUCUUCAUCGGGUUCACUGGACAAUUAUUUCGAAAAAAACUUCCCA

>P318

AGCACUCAACUACAAACUACAAAGGACAUUGAUCAAAAGAUGAAGAAAAUG

>P319

ACAGGUCAGCAUAAGCCUAAGAAAGACGUUUGAAAUCGCUAAUCGGCUUUU

>P320

UUUUGCUACGGGAUCUAUUGACGGGACAGUGAUUAUCGGAUCGAUGGAUGA

>P321

CAUUGAGUACGAGGAUCUUGAUGAGACCAUUAACAAGAUAAUAGAAGAACA

>P322

AUCGCUCAAAAAAUGCUAAUGAAGGACAAUCCAAAUCCAAAAGGCAGAUGA

>P323

ACGGCGGAAACCAGAACUCCAAUGGACAGCACGAAUCCUUUAAUUUUUCUG

>P324

GCCAUGAACAUGAAUUUGAAUAUGGACACUAAAAUCAUGGAGAACCAAGAC

>P325

CAUGAGCAAAACAUCAUUCACAGAGACAUCAAGCCAGAAAAUAUUUUAUUA

>P326

GACAAAUCGGUGGAGGCAAUAACGGACAAGUAGACAUUAAUUUGCAAAGGC

>P327

GUGACAGCGAAAGUUUCCAGCAAAGACAUUUUUAAACUGAAGAAAAUGGUG

>P328

GGAAGAACUUUGAAAGGUAUUAUGGACAAUGUCAAAUAUAUGGUGAAAAAA

>P329

GUGAUUGUGGCACGUAGAGGAAUGGACGAGAAUACAGCAGCUACUGGGAUU

>P330

UGGUGUUUGUCCGUUAACGCAUUGGACGAUGUAGAGGAAAAGAGUAGCGCG

>P331

GCAAGAAUCCUAAUGAAAAUAGAAGACGAGGAGGACAAUAUAAUUGAUAUG

>P332

AAUGAUAUCAUUAAAUUGUGUAAAGACGCAUUAUAUUUGCAUGAGAGCGUC

>P333

AGAGGCCGACCAAAUAACCUCAAAGACAUUCUAUAUUUCAACCUUAUUUCC

>P334

AAAAAUGGCGAAGAUGAGGAUACAGACAAUUUGAUGGGAACAGAAAAUUCA

>P335

AGUAAUAGACACUCUUUCAGAAGGGACGCUCCUCCUGCAUCAAAAGAUAGC

>P336

AAGAUAAAUAUGAUUUUAAAAUUGGACUUUCAGAAAGCGAAAACGCGAAGA

>P337

CAACGCAAACAUCUGGACCCCACGGACUAUCGAUUUUUAGGGCUCGGGCAU

>P338

CAAUAAGCAAGGGAAGAGGGAGGAGACAAAGGGAUGUUUUCAUUGGGAGGG

>P339

CUAGAGAAUGAUAAGGCGCCACUGGACUAUGAUCUUGCUAAACAUUUUGCG

>P340

UGAUAUUGCUGCAGGUAAAGAACAGACUUUUGGAACCUUAUUUGCUAGAAC

>P341

AACGUUAUGCUCGAGAAGAUUAAGGACAAUAAGGAAAAAAUCAAGAAUAAU

>P342

CAUUUUUUCAAGUUUUUCUUUAUGGACGACAAACCAUCAGACAUUUCUGUC

>P343

UUUUGAAAAUGAUGUCGAGAAGAGGACAUGUUUCCAUUGCAAGACGUUAAA

>P344

GCAUACGACGACCGAAGCGCUCAGGACGAUAGCAGCGAUGAAAGUGAUCAC

>P345

GAAAAGGCUGCCAAUGCUUUGAAGGACAUUUACGGCUGGACUCAAACCUCA

>P346

CCUGAAAACGAACAACUGGGGUUGGACUGUAUAAACCACCUCGUCUUAAAC

>P347

CCCUCAGUGCAAAUUUAAGAAAUGGACAUUGGGCUUUAGACUACACUAUCA

>P348

GAAAGCACAAGAUAUUUUUUAAAGGACGAAAAAGGAUUGACGAAGGGGAAA

>P349

UGUAUUGAACGAUAUAUGGGAGAAGACUUUAUCAAACUAUAAUCUGACACA

>P350

CAAUUGUGGAGUUCUGCUGAAAAAGACGAUCCAUUUUAUAUGAAUUAUUUA

>P351

AGGAUAUAGAACUCAUAAUCUAUAGACGUUGUGGAUAUCUAGCCGUAUAUC

>P352

AUCCAUCAAUAGAUAUGUCUUGAGGACCGUGCUACCCAAAUGGACUGAUUG

>P353

UAUAAAUUAGCGCAAGAAUUCAGGGACAGUCUAAUGUUAUCGCACUUUUUC

>P354

UUCGUUUGCAUAUUAGGUUAAAGAGACUGUGAUUAUAUAUACAUGCACGUU

>P355

UCAUUGCGUACUCAAAAAGGACAAGACUAUUUGCAAGUAUCGCAGACGAGC

>P356

GACCCGAAGAUAACUGGUGAAAAGGACAUUAGAGCUCAGGUCAAACUGGCG

>P357

GAGGCUUAGAAAUUAACGAUUAUGGACAAAACAUGCAUAGAAUAUCAAAUG

>P358

GCUAUUGCUCAAAGAAUUGUCAAGGACUCUCCAGUUGAAAAGACUGUCAUC

>P359

CGAGUACCACGCAAGGGUAUGGAGGACAAUAGUGCAUUUUCUGCAGCCUCA

>P360

AUGGGCGCGAUAUGAAAUAUCAAGGACGUUUCUUGCGUUCCAAGUUUUUGG

>P361

GACACAAGGUGUAGUGGCUAUAAAGACAAUGAUGACAAAACUGCAUACACU

>P362

CAAAUGCAUGCGGCCCUUGUAUCGGACAAUGGAAUAGGGAAGAUGUCUCGA

>P363

AAAUCAUCAUCUUCGUUACCAAAGGACGUUAACAAACAAGAAGAACAGCCU

>P364

GGUUUGGUUGAACUACUAGCAAGAGACUUUAUAUUCAAUCCUCAAAGAACA

>P365

AUAAGCCUUGGAAUUGUCCCCAUGGACGUCCAACAAUGAGACAUUUAAUGG

>P366

ACGCGUUACAAAGAGCAAAAAUAAGACUUUAAUAGGCCUUCAAGGCAUUGU

>P367

UGGCUUAAAAUCAUUUAAAAAAGGGACGAUAUUUGAAAGAGCUUUGCCGAG

>P368

AGAAGCAUAAAUUCUCAGAAAAAGGACAACAAACAAAGCCAAAAAAAUCAA

>P369

CAAUCCACAUCUGACGAAACCAAGGACUUGUUGUUGUUAGAUGUUGCUCCA

>P370

CAACCCUCAGACGUUGACCCAAGAGACAUUUACAAAUCGAUAAUGAUGCUA

>P371

ACUAUUACUUUACCAACAUUGGUGGACAAUCAUUCAAGAUAGCGAAAAAUU

>P372

CUAAGAAAGACAGUAUCAAAUAUGGACUCAAUGAACCACCGAAGUGCGUCU

>P373

GCUCAUAAUACAAAUACCAAUAUGGACACCUCUUCUUCGCCAAGGGCGAAC

>P374

AACAAGCAUAAGAGAGCAUUAAAAGACAGUUGCAAAAACAUCGAACCAAUU

>P375

GACAAAGCUACCGGCCAAGAAAUGGACGUUGUCUCCGAAGAACCUUUAAUU

>P376

CCGGUGAAGAGGCCCAGAGAAUCAGACAAUGAUUAUGAUGCAAAUUCUUGG

>P377

GUACAACAUUACCAAGGACUUUAAGACAGGCAUAAGGAGAAGGUUGAAAUU

>P378

GAAAAGCAAGAGCAGCAAGAGCAGGACAAAAAGAAAAAAUCUAACCAUUAA

>P379

CAUUGAAGCACCACUUGAAGAAUAGACAUUUGCAAAUGAUUGCCAUCGGUG

>P380

UUUGUCGAAGACAAUAACCCCACGGACUUUUUAACAGAUAGAAUAAGAGAA

>P381

ACGAAAAAAUACUGUGAGAUAAUGGACGAUAACUCACAACUCGACUUUACU

>P382

AACUGGAAAAUGACCCAUGCCAAAGACGAUUCAGAAGUCGCUAGAUUAAAG

>P383

ACCGCUCUUCACGCCCGAAAGUUGGACAUUUUAAAUUUUAAUUCUCAUGAU

>P384

GCCUUUACUAACUGGAAUAAGAGGGACUUUAUGGCGUUUAUCAACGCCUGC

>P385

AGAAAACAUAUGCACGCAGAAAGAGACUUUUAUUUAGAUUUAGUAUAUCUC

>P386

CGAAAGAAACGUGUAGGCAAUUAGGACUUGGUACAAACAUUUAUAACGCAG

>P387

GUCAAUAUCAAAUAUUUUGGCAAGGACGUUCCUACCAAGAGUGGGGAUCAG

>P388

CUAAGGGACGAAUCCUUGACUGUAGACAAUCUUUCAAUCGCCAUCGUGGGU

>P389

CAGGGACAAUUGUUGGAUAAUAUGGACGAGGGUAUGGACGGUGUUGUAAAU

>P390

AACUUGGACAAGCAAGUGAAGCCGGACAUUAAAAAGAGGGACUACUUGAAU

>P391

ACCGACAAGGCAGACUCGGGAUUGGACAAUGAAAAAUGCCCGAGGAUGGGC

>P392

UCGUUCGAAUUGCGUACGGAGAUGGACUGAAGAAUACAAAAUUAGGUUCUG

>P393

UAAUGGCAUAUAAUGCCGCCUUGAGACACUAUAAUCUUCCUGAUCACCUUG

>P394

AAUCGGUAUUGUUUCCCACAAGGGGACGGUUAAUGAGGGACAUUAUAUUGC

>P395

CGAGUGUAGAUGACGUAGCCAAGGGACUUGCCAAGACCGAAAUAGCAAAUC

>P396

CGUAUCAUUGAAGAGCAGAUAACGGACUGUACAGACGAUCAAUUUCAAAAU

>P397

GAUGAGCAAGACGGUUUAACCAUGGACGUAGAACAGGUCAAGAAUGAAAUG

>P398

AACAAAUGAUAUGUAUUGAACCUGGACAUGUUCAUGAUUUUAUUUCCUUGG

>P399

UCAUCAAAUUUGAAGCAUGUAAUGGACAGACUAAAUGAAAUACAUGAAGUU

>P400

GAAUGCUCAGAAGGCUAUCGAAAGGACACUACAAAAAUUUAAGGAAUUUGA

>P401

AAAAUAGCAUUAUCAUGGGCCAAGGACAAUGGAUUUUUGAUAAAUCAACUG

>P402

UGCGGAGUAUUACUUUAUCCUGAGGACCCUUAUGAACAUUUUAGAGAAGCA

>P403

GGAAGUUUCUCGGAGCGUUCUUCAGACGAUAAUAAUGCGAACCAUCCGGAA

>P404

UCUUAUCUAAGAGAUUGCCAAGGAGACAAUUCAUACAAUCGAAUUCUUAUA

>P405

AACACAUUAUUGGAAGAGUUCAAAGACAUUAAAAUGGUGAACGGCGAGUUG

>P406

AAAUAUUGUCGAGGCGUUAAAAGAGACUUUUGAGACGAAUACUGAAUUUUU

>P407

CUAGAUGCCAUUGCCGAUUGGAAGGACUUAUUAAGUGGUGGUGAAAAGCAA

>P408

GUUUAGUGAAGCUAAUGGACUUCGGACUAGCCAAGAAACUUCGGAACAAUA

>P409

ACCACUUCUUUAUUAACGUGUAUGGACAAUUCGCAAUUAACGGCAUCAUUC

>P410

GCAUCUUUACUCAAUGAAUCUAUGGACGCUCUAAAGAUAUCAGAAAAUGAG

>P411

AAAACUGUCCAUUAACAGUGCAAAGACUUUACAAAACUUAUAUCCUAAUAU

>P412

GUGCACAAUAGACCAAUAUAAAAGGACAUUUUUUGGAUUUUCUAAGGGAAA

>P413

GAACAAAAGCAUGAAUUGUCAAAAGACUUCAGCAAUUUGGGAAUAUAAUUC

>P414

UACUCACCAUCUAAUCAAUGCAGAGACUAUUGAAAAAAUGAAAGAUGGUGU

>P415

AAAAGAUGAUCAAGCCAAAGGAAGGACGUAUCGAAAAAUACAUUUUAAGAA

>P416

CAACAAGAAAAAAUUUUAUGCACGGACUAAUAUAUAACCUCCCCGACGCAC

>P417

GCAGAAGUAUUAUAAAGGAUUAGGGACUUCUCUAGCACAAGAAGUCCGAGA

>P418

AGCGGACUCAGGCAAGACUACAUGGACACUGAGAAUAGAAGGUAAGCUUCU

>P419

UUACCGCAUUAUUUUUAAACCGUGGACAAUCAUGUUUAAUAGAGUCUUUAC

>P420

UCAAGACAUAUAUACAAGGAAAAAGACGUUGUUUUGGCCCAAUCUUGUAAA

>P421

GGUGCGUUAGGAAAGCAGGCGCAGGACAGUGAAGUUAGGAGGAAAAGAAAA

>P422

CAAGUUGAGAAAUUUGUUCAAAAAGACGUUAACAAUGCUUUACAACCUUUC

>P423

ACGUUACGUUAAAUUUACCGGAAGGACAAUACUUUUUAAUCCCAUUUUUAU

>P424

UUGUCAACACCAAUCAAUUCGAAGGACUUUUCAAAUAUUAUCGCUGUGUAG

>P425

AGCACCUACUUUAAGAUUAAGGUGGACUGGCCAGUUAUCUGAUAAGCCGGA

>P426

UUACUGAGAAAUGGCCGCUAUGUGGACACAGAUUUCGAUUCUAUGUGACAC

>P427

AGAAGAAGAAAAAGACAGGAGCGAGACAAAAAGAAGAAGGGUUGCGGUCAU

>P428

UUGAAGAAGUCCGCGAUCAUAAAAGACAUUGAUUACCUUUAUACCUUUAUU

>P429

CAUAAGGAUUCUAUUCACUAUAUGGACGAAUACAAGGACAGGACUGUUUUA

>P430

UUUCAAUUUAUUCAGCCUGCAAGAGACGAUUAAUGCUUUAAGAAAUAAAGA

>P431

UUGACUGCUGACUACGAUGCUUUGGACAUUGCUAACAGAAUCGGUUACAUU

>P432

GCAAAGAACGCUAUUGUCGUUAAGGACUUCCCAAAAUAUAGCUUAAACCCU

>P433

AUGGGAGAUGAAAGCCGCAAAGAGGACGUUAAGGAAAAAAAGAAGAAAAAG

>P434

GUUAUGAACUCUAUACACAAAUAAGACGGUUACAUAAGCUUAUGCCGUACA

>P435

UGGCUAGGAUCAUCAAACAAAACGGACUACUGUUGAUUGCAUCGGUCGGGG

>P436

CCAAAUCAAUGGGAAGAUGACAAGGACUCUGUUGUUGGCAAGCAAAACGUU

>P437

AAUUCUUGGUUGACAACCAAAACAGACACUAUUUCAUUGAAAUUAAUCCGA

>P438

GAAAAACUUCCCAGUGUAGAAAAAGACAGUAAUAUAGUGAUUCUUUGCCGC

>P439

AUGCAAAUUCUAGAUUUAUGGAUGGACUAUCUAAACUAGAUCGCUUACACG

>P440

UAAUUCAUUAAAUGACGAGUUUUGGACAGACCUUUUCAUGAAUGAUAUUUG

>P441

UACGAUGAAGAAAUUGGACGAUUGGACACAUUAUACUUGCAACGUCUGUCG

>P442

CGGUGGCUGUUUGUGGGCUACUCAGACAUUAGGGCCAAAUACGGUAUAAGG

>P443

CCUUACGAAGAUACUUCCAACAUGGACAUAGACGAAGUAUCUCAACCUGAU

>P444

ACAUAUACAUUGUACCCCCGGAGGGACGUAUACAUUGGCAUGAUAAUGUAA

>P445

AAUCUAUCUUCAGCAUUUCCCCAGGACGAUGAAGUGAACAGCUUAGCAAAA

>P446

UUCUUCACCCUUUUUUAUGCUAAAGACAUUGGAAUUUGAUGCAAACACUAC

>P447

ACGCUGCAGCCAUAUGGGCCACGGGACAGACGUGGUGGCAGAUCCCACCAG

>P448

GGCAACUUCUGAUGAUACUUUUAAGACAAACGCCGCAAUUGACCGAACUAU

>P449

GGAAGUAACGUUUAGGGCCUUAGGGACACCUACAGAUAGAGAUUGGCCCGA

>P450

ACUGCCAUCGUACGUUCCCCCAAAGACAAAAAUUAAUCCGUCAUUUAAAAG

>P451

GAGUCAGAAAAUAUUGUUACUAAGGACCACAAAAAUAAGCAACAAUUUUCC

>P452

UUUAACGAUGAGGAUAUUGGUGAAGACGAAGGUGCCUGGGAUUUGGGUGAC

>P453

CCUAUCGCGGGGACGCAUGGCACGGACAAUUCAGCUAUGAGUUCUGAUCUA

>P454

AGAAGCAUUGCUGGUAAUUCCAAAGACUUUGGUGAAGAACUCAGGAUUCGA

>P455

CAUCGAAGGUGUUGGAACCAUCAAGACCGUUUUGGGGGCUUGUGGCCGCAG

>P456

AACGCCGUGGCAUCUUUAAAGAAAGACACCGCAUGGACAGGUGUUGAUUGG

>P457

CAAUGGUAGAUCCUCUAACCCUGGGACCCAAGCUAGAAGAGGCGGCAGCCC

>P458

UCCAUCCCAUUUGAUCCAUCAAAAGACACUUUUGAUCAUUCUAAAUUAUUU

>P459

UUGAAGAUAGGAUUUACUACAAAAGACAGUCUUUCAAAGAUAGGUUUAAAA

>P460

UUUUGGGCUCACUACUGUUUAGCGGACUAUUUAUCAAUACUAAGAAUAUUA

>P461

CAAAUGUGAUUCCGGACAAACGAGGACUUUACGACCCUGAUUAUGAACAUG

>P462

UUCCGCCUACCUGUAAAAAAACAAGACAUUCACCAUUAUCCUAUUAUCCCU

>P463

UGACCAAAAUUUGUUUAUUUACUAGACCUGCAUCCCAGUACCUCUUCCCAG

>P464

GAAGUGGCUCAAAUGGAACAAGUGGACUUUCAAGAUUCUAAGGAUGGGCUU

>P465

CAAUAAGCAAUUGACUAAUCCUAGGACGAUUCGUGUACUAUAAGGAGAAAA

>P466

AGGUAUCUGAGUGGAUUUUCAAUGGACAUUGGCUAUUUACUACAUUUCCCA

>P467

UUAUUUGGGUUACUCACUUCCGUAGACACUUGAUGGUUUGGAAAAUCUUCU

>P468

AGGUUCCACGACCUUUUCGGUAAGGACAAUUAUUGGAUUGAAUACGAAACC

>P469

GAUUGCCUACGGGAUCACGAACGAGACUUUACGGGGGCAGUCGAAUCGCUG

>P470

UGUAAGAAGCAAUGUGAGGGCAAAGACGCUAAUUCAUUCGCUGUUCCCAUU

>P471

CUGAUGAUGAAAUCAAAUCCGUUGGACGAUAUAACGAUUUUGGAUGAACCC

>P472

AUCACUUGCGGCGACGAGUGUCUGGACAUUUAUGAACGGGUAGCCUCGUCG

>P473

GUUUGCAAAAGAAUUACUAAAAUGGACAUUUAAUGAAAAAUCUGUAAUCAA

>P474

UCCAUCUUGGACUAUGCUGGUAAGGACAUUACUGAGAUCAUGAAAGACUCA

>P475

GAAACUGACACUGAUACUGAUACUGACGCUGAAACAGAAAAUGACAUUGAC

>P476

CAAACGACAUAAGUCUGUUACUUGGACUUUUAAUAAUGGAAGCAAACCCAA

>P477

GCUGCUGACCGUGAUAAUAAAAAAGACGAAUCUACUUAGAACAUACGACUA

>P478

CCAACUAUUAUUAUUAUUUUCAAGGACGAAACUCACCAUUCUCACACAUUC

>P479

UAGGUGGCACUUUGGGUGGUUCAGGACAUAACUCCAAAGCUGCAGACUUAG

>P480

CCAGGACAAGACACGACAGAUUUGGACUCAAUUUAUAACAGUAAAUUACAC

>P481

UGACUCGCCGCCACAAAAUUCAAGGACUUGUUAUAGUAAUUUUGCUGUAUU

>P482

GCGAAAAACCACGAGAGCUAAGUAGACAUUUAGAAGUAGUUGAUUUUUUUG

>P483

ACAAAAAGUGUGAAUGACCAAAAGGACUCCUCGAUCGGCCAUUCCACUUCU

>P484

AAAAACAAGCUCAAGUAUCCAAAGGACAAUUCGGUAUACCAACAUUCUCAC

>P485

CAAGAAGAUUUCAAUAAUUUUAUGGACAGUAUGAAAAAUGAAAGCAGCUUG

>P486

GACACAAGUAAUCACAAGAAACAGGACAAUAAAGAGAUGGAAGAUGGGCUG

>P487

AGCAAUUAGCUCUUAUUACAAGAAGACAAUAUAUUUGUACGAAAAGAGACA

>P488

AGCGAAGUCACUGAUUUGAUUAGGGACACCAAACACACAUCAGAUUUUGAU

>P489

AUGAGCUGAGUGUAACGCAAAAAGGACUUAUAAUGGGAGUACAAGAAGAAC

>P490

CAAACAAACUCUUGAUGAAGUGAAGACAUUUUUCGAAACCAAUGAGAAUGC

>P491

AAGAGUAACUAGGACACUGCUAAAGACAUUUUUGGACAUAAACCGUGUUUU

>P492

GAUCUACAUUCUAACAACAUAAGGGACUUUGUUGACGGUAUGGAAAACCUC

>P493

AGACAGUACAUUUGUACCAAGAGAGACAUGACCUAUGUUUUCGCUAAAUAU

>P494

CGUUACAACAAUAAACAACAAUAGGACUCCAUUGCUAGCCACUCAGCAUAA

>P495

GGCCAAGGCAAGAAUGGUGGGAAAGACUUUACGUAUGGAAAGAAGUGCUUC

>P496

GUACUGUUGGGUAGCCGUAAUACAGACGUUCAGAAGCUUGCGUUGGACGCA

>P497

UCAUGCCAAGUUACUAAGUGAGCGGACAAAUACUUCAAACCAAUUUGAACA

>P498

AGGAACAUAGUGGUGGCAUGUAUGGACAAUUCGAUACGACUGUUCGAUGUU

>P499

CCCCCAGAGUUCUCUCAAUUUAAGGACUCCUAUCAAAAAGACUAUGAGAGA

>P500

CCAAAUGUUCCACAAGAAUUGAAGGACUUAUACAAGACUGUUUGGGAAAUU

>P501

GCAAAAAUUGUAGAUGAAGUUUGGGACAGUUGCUUCGCCGAUACGAGACCG

>P502

AAAAUUUGGUAUCAGAAAUGCAGGGACAGAGAAAUACAGGCUGCCUACGGC

>P503

GCUAAACGUAAAGCCAAUGAAUUGGACGAUCUAAAAACUAAUAAUGAAGAU

>P504

GGAAACGUUUAUAAUUUACAGCCGGACGGUAACACUUUUGAUAUCUUCAAG

>P505

CAUUGAACUGAUAUCUAGCUCAAAGACUAUUUAUUCGAAGUUUACGGACCA

>P506

AUGGUUAUAAUGCCAUAAUGUGGGGACAUUCCAUGACAAAGCUAUCUGAAG

>P507

ACAUAACGCCUUGCAAGCCCGGCGGACAGCUCAAGUUCCACAGGAAAUCUG

>P508

UUAGUCAAGCAAGAUGAGGUAAUAGACUCUCAAUUUUUUUAUAAGGAAUAC

>P509

GCUUCUUCAUCACCUUCAUCAACAGACUUUUCCACUAGUAUUGUUUCCUUG

>P510

CAACCCUUAGAAACAACAAUUACAGACGUUGAAAGGUUUAAGGAUACUGUA

>P511

CAAAGCAGAUUCAUGUUGCACACAGACGUUCCCAAAGUGAGUUGACAAAUU

>P512

AAUGAAAAUCCGGCACGUUAUUGGGACAUUUUUUAUAAAAACAACAAGGAA

>P513

GAUAAAGGAUACAGUACUGGAAAAGACAUUUUUGGGAACCUCCCUUGGACA

>P514

UUGGGUAUAGUGAACAUUAUCAAGGACUACUUGUUGUGCUGUGUCAUGGAA

>P515

AAUUUCAGAUUGAAUGGGUAAAAGGACACGAUGGUGAUCCAGGAAAUGAAA

>P516

GGAUAAUAAUGAAAAAACUUUAAAGACAAACUUGACGAUAGGCGACAAAAC

>P517

GUCAAGUUGGUAAAAAAGACCUUAGACGUUAUGACCUUGGCCAUUGGUGAU

>P518

AUUCUGGGAGUUAGAUUAUUUGAAGACUCCCUUGAAAAUUAAUAAAGACGA

>P519

AAACAAUUCAAUUGAGACACCAGGGACAGAAUAUUUACAGAAGUGGUGUAU

>P520

GGCAAGCUUCUGGCAAGUUAAGAGGACAAUGAAAGUGGUAUAUUCCACAUU

>P521

AAAAAAUUACCAAACCCGCAGUUAGACGUUAUAUUCACUUAUACUGAUUGG

>P522

AUUGGACUUUGAUGCUAUCACUAAGACAAUUGUUAAAUUGCCAUGGUACAC

>P523

AUUAAGGGACAACUUGACCUUAUGGACCUCUGAUAUUUCUGAAUCUGGUCA

>P524

GGUUGGAGGCCCAAACCAAACAUGGACACGAUAAAUUUCUUUUUGAACUAU

>P525

CACUUCUAGUCAAGAUCAAAUGCAGACUGACAAUAACAUUGAACAGUUAAU

>P526

UUUCCAAGUGACCUUAAAAGCAAGGACGAUUAUAAAAAGGUGCUUCUACAG

>P527

ACAGCAGACAUGGUUCUUGAUCCGGACCGCUAUGAAGAAGGACUGAAAAGA

>P528

AGUAUCAUGAACAAUAGCCGCUAAGACACUUCUAAAGAGGUCUACUUAGCU

>P529

GAGUGAUAAAAAGUCUUUGGAUGAGACGAAGGAGAAACCAGAGGGGAAGGA

>P530

UUGAUAUCUCCAUUCCAGCAUGAAGACAUAUCAGAAGACACGAAAGAACAA

>P531

AAUGCCAAGUAAUGGAACUCUAAAGACACUUGCUAAACCUUUACAAGUACU

>P532

CACAUGUCGUUUACAACUCCAUUAGACAAUUGUAUGACUAUGAAACUGAAG

>P533

GAGGAACUGGAAAAACUUUCUAGAGACUUUGAUAACAUCCAAAACUUUAUU

>P534

UGUUUGGCGAGAGGCAUGUUACGAGACGAAAAACAAUUUGUAAAAGUUAAC

>P535

GACAAACUAGAUCCUCAAGAAUGGGACAUCAACGAACGUUAUUACACUAUU

>P536

CAAGUAGAGGUGUAUUCUGAGGAGGACGAUGAGUACUCUACUGAUGACGAC

>P537

GUACCAUGUUUUCGACCCCGAAGAGACGCUCAAGGAGUUGUAUGAAUAUAC

>P538

ACUUUUAUUACAGUUUCACGUAUGGACACUUACACUCAAUACCAGAAUUUU

>P539

AGUUUUAUCUAUUUGAGGAAUUAAGACAUGUUAGUAGCGAACUGAAUAGGU

>P540

CCAAAAGGAAGGUGAUGAAGAACGGACAUAAAAAGCAAAAUGACUUGCUAG

>P541

GGAUAGAUGUCACAGAAUUGGACAGACUCGAGAUGUGCAUAUUUACAGAUU

>P542

ACACUUACUUACAAGAAUCUAAAGGACCGAGGACACCUGCUAAAGAGAUAC

>P543

GAGAACAUUUUUGACGCAGUAUUGGACAAGAAAUUAUUAUGAACCUGGCAU

>P544

UUGCCGAAACAAAACUGACGUCAGGACUCUACUAAAAAUACUAGAAUGGCA

>P545

AGUAUCGUAGGGACUAUAUCACCAGACUGAAACAAAAGCAUAUACAACAUU

>P546

GAAAAGCAAAAUUUAAUAGUAAAGGACGCUAAUUGAAGGCAAAGUUUUACU

>P547

GAGGCCUCUUAUCGAUGAUUACAGGACAAUACUUGUGACGGAGGGAGCAAA

>P548

AAAUCUCAAGAGACUGCAAAGAAAGACGAUCGGCCAAAAUUGGUGGCAAAU

>P549

AAGGUGAACGAAUACCUAAAGAAAGACAUAUAUGACUGUGCUACCAUCCUG

>P550

UAUAUAACAAAAGCUCGAAUGAAAGACGGUUGGCACAAGAGAAUUAACAAA

>P551

UGCUGGUCGUGUCUCAAAUGGAAAGACUACAAUUGAUAUCCAAAGGUAUCG

>P552

ACAAACGCUGUGGACUUUAAAAAGGACGUUGAUGGGUUCCACAGAUAUAAU

>P553

AGUCAACAACGGAAAAUUAUAUCAGACUCAAGAAACUGUAUGAAGUAAAAG

>P554

AGAGAUUCUUGACUGUGAAAUAAGGACGUUGUGAAAGAAUAAUACAAAGAG

>P555

ACAAGUAACAGACCCUUCAGGAAGGACUCAAUCAAUUACCAAAAAGACAAU

>P556

GCUGAUAUUCAAUUGGAAUAUUUGGACAAUGUUAAAGAAGCCGUAGCUUUG

>P557

CUCUUGCCUUCCCGCGACCUAAUGGACCAUCUGGAUUGAACCCUUUAUUAU

>P558

AACGGGAAUUUAUCGCUAAAAAGGGACAAUGCACACAUUGCUCAUGUUAAA

>P559

AGAAAUCCAGGGGUAUCAACAAGGGACACAAAUUCAACAACACCAAGGCCG

>P560

UUUGACUACAAAAGAACGGGACAAGACAUUUGAUGCUAUGGCUAUUGAUGC

>P561

GCUAACCAAACUAGAGCCAGAAAGGACUUUCGAUGUAAUGGUAACAUCAGU

>P562

AAUAGCGAGGAUAAUAUGGUCAAAGACAUAGCCUCGUUAAUAUAUUUCCAA

>P563

UCUGUGCUGUUCGUGAACUUCAUAGACAAUGUUAUUGGUGAGACAAUUAUA

>P564

AAAGAGAAGAAUGCCGAAAGUAAGGACAGUGAUGGCGUACAGGUAGCUAAU

>P565

GAACCAUUCGCAUUUGAGUAUAAAGACAUGGAAGUUGCUGUUGCUAGUGCA

>P566

ACCAAAUUAUUAGAGAUUACUAUGGACAUUUAUCGGGGGUUCGUACGGUGA

>P567

UAUUUCAAAGUUGAUUAUAUCAAAGACGUUAGCCCCAAAUUACACAAAUCC

>P568

AAUUUGCGAGAAGCGCGUUGAAUGGACGUUUAAAAUAUUUUCCGCAAGCCU

>P569

CUAUACUAAAACAGUUGACUGGUGGACAUUAGGUAUCUUACUGUAUGAGAU

>P570

GUCGCCGCAUACAAAGACGGUAAAGACAAGAAGGCCAGAACUGACAUGUGU

>P571

AGCACCACGAAUAAUAUAACAAUAGACAAUGGUAAUAAUGACAACAAAAAC

>P572

AAGUACAUAAAUGGUUUGCAUAAAGACGUUUAAUAGAAAGAAGAACAAUGU

>P573

AUAUGUGGCUACGGUUAUGGACUGGACGGUAACAUCCGUUAUAUUUACACU

>P574

AAUUUGAUUGUAAAAUAUAAAGUGGACUAUCCAAUAUCAUUAAACGACGCU

>P575

ACAUAAAAGCUUUGCAAAGUAUUGGACAAUUGAUUGGCGAAGUCAUGAAGG

>P576

UGUAUGGGAAGAUCAUGAACCAAAGACUUUUUGGGACAAAUUUUGGAAUGU

>P577

AUGAACAACAGUCAUUCGCAGAUGGACUUCGACGGCGACCAACACAUGGAU

>P578

GCCCAAUCUACAGAUGAAACACCAGACAAUGAGAACAAGCAAUAGUGGACU

>P579

ACAAUGGAUUUUCUUCUGAGUAAGGACAUAGCUUCAAGAAAAAGAAGCCUG

>P580

AUUCCCGUUUUAUUCACCUGCGUGGACUUUGCAACUAAAAGGUGAAUUGGC

>P581

AAUCAGCUACCCCACAACUCAAUGGACGAUAAAGAUUUGGAUUCAAGAGUA

>P582

CACUGAUUCACACACGGUGCAUAAGACAUUUUAUGAUUAUCCAUUAACUAU

>P583

CACCAGUGGGAUCAUAAAUGAAGAGACGUUUAACAAUUUCUUUCGAGUUCA

>P584

ACUAUUAGCUAUAAAAGGAAAAUGGACUAUGAAACAAUAUUGAGUAAUAAU

>P585

GUUUUGAGGGAUAUGCCAGAAGAGGACUUUGAAAAACACAAAGAGGCGUUA

>P586

CCACAGUGGAAGAAAUUGAUGAAAGACAUUAUCAACAACGACUCUACAAAC

>P587

AAGAUGAGGCUUUUAAAUCCAUUAGACACUCAACAAAAGAAAGCAAUGGAA

>P588

CAACUCUCUUGACAUCACUGCAAAGACCAAAUUAUUAAAUGAUUCCUUGAG

>P589

AGUUAAAAGCUUUCAGGAAAGUUAGACCCGUCCUACAGAGACAUAGCUCUU

>P590

AUUAUCUGCCCAAGAGGACACAAGGACGAUUUUAAAUGUAAGAUAAAAAAA

>P591

UAUAGGAGACAUAUCAAUGAAUUAGACGGGAAAUGUCGAGCUCUUGAGGAA

>P592

UUGCUAUUCCUCCAUACGUACAUAGACCCUUGAACCUACAUAUCCGCGUAA

>P593

AAAGGAUACAUUUGAAUUGAUCAAGACAUUUUUAGUAGAUUUAACUUCCUC

>P594

CAAAACGAACAUGCUACGCUCCAGGACAUUUCGAACUUCGAUAAAUACAAG

>P595

GCAUUUCCUAAUCAUGAUGUAAUAGACCAUAAUAAAAUGCUAAACUCAAAU

>P596

AGUAAGCACGAGGUAGAGGCAAAGGACAACACCAACAAGGGCCCUGACGUU

>P597

AAGAUUAUAAAUACGUUUUAAAGGGACAUGAAGAUAUAAGACAAGAUAGCC

>P598

GUGAGGAAACUGAAUACCAUGGUAGACCUUUCAAGAUAGACGUUCAAGUAG

>P599

AAUAAUGGACCCAACAAUGACAAGGACGAUGAUGAUGACAAAGACAAUGAU

>P600

ACCUUGUGAAUGAUAUGAGAGAAGGACUUUUAAAAAUUCUCCGAGCUUACC

>P601

GCUACAAGUGGUUAUAGACUAAUGGACAAAAACUACCCUAAUAGUGACGUU

>P602

UUAGGAAAUAAGUUCUUCGGGAUGGACAGCUUGAAACAAUUGAUUAAGAAC

>P603

AACGAAAUUAUCAACGUUGGAAUGGACUCCGCCGCUCCGUAUUCGAGUGUU

>P604

CCCGACGAUAAAUCGUCGGGCACAGACAUUUAUAUGGAUAAGAUGUUCAUU

>P605

AAACCCUCACUAAUCAACAAGUUGGACUUAAUUUGUGCAAGCAUGGAUGUU

>P606

AAGAGCACUCUUAAGAACGUUCUGGACAAUCAAGAAACACAAAACAUUACC

>P607

UGUACUACUAUGGUAAGAGGAUAAGACUUUGGACUAAGAGAUGGUAUUUGC

>P608

GAUCAAAGUUUGGCUCAAUCACUGGACACUAUUACUUCGAAAGCGCAGUGG

>P609

CCGUUCAAGAAUGGGAGGUAUUGGGACGGGUGGACGUUUAAGCAUACAUUU

>P610

ACAUUCCUUUUCUCAUAUUGUGAAGACCAAGAAGGAGUCCGAUCAUGUUUU

>P611

UUACCAGGCCCAGAAGUUCCAAAAGACGCCGUCAAGGACAGAGAACAAUUU

>P612

UGCUCUUCAACAGCCAUUUCGCAAGACUUCAAGAGGUUCUUUAUCUGGUGC

>P613

UCAUUGAUGCUCUGCUAUCGGUAGGACUGGCCAAUGCUAGUGUCGUUUGUU

>P614

CAGAUGAUGAAAAAUCGCAACAGGGACAAUCUGAUGACAAGACAACUACUG

>P615

ACGAAAUCAUCACAUACAGAGACAGACUAGAUGUAACGGCCAAAGAACUAU

>P616

GACGAUCCACAACCGGUAUUAUUAGACUCUAUCUCCGUCAAACCCAAUACC

>P617

GCCGAUGGGGUUCAUGUGGGCCAGGACGAUAUGCCAAUCCCAAUGGUAAGA

>P618

AUCAAGGCCUUGAGACUGUUUAAGGACGAGCCCAAAUGGCAAGCUAUCAAC

>P619

UCCUCCCAACGUUCCUGGUAUUUGGACACAUGAUGACGACGAAUCGCUAAA

>P620

GAAGAGGCUGAAAAGCAAAAUGGAGACGCAAAAGAAAACAAAGUUGAUGCG

>P621

AAGUUUUCUACAAUAAGAAGCGAGGACUUUUUGCCAUACAGCAACGAACAC

>P622

CGGUCAAAAGGGUACUGGUAAAUGGACUGCAAUCAACGCCUUGGAUUUAGG

>P623

UCUUUUAGUCAAUCGAAUGAUUUGGACUUUUUGAAUAAUCCAAGCGGAUCA

>P624

GAUGGAGUUUUGGAAAGGAUCUUGGACAUUUCGGCAGGUGACUUGAGAAGA

>P625

ACGGAUAUUGUAAGAUACAAUAGGGACAUUGCACCAUUUAGUGGGCUAUCU

>P626

CAGUUAAUAAAACUUAUCUAGAAAGACGUUUGUUUAUAAAAUAAUGUAUAG

>P627

AAAAGUGUUUAUCUUAACAAAAAAGACAUUUUAUAUUAUUGGACAAACACG

>P628

AUUCGACUAGCACUACGAAUACGGGACUUUUCGGUGCAAAGCCUAAUUCUC

>P629

ACCUCAAAUAUCCUCAUCGUGACGGACACUCUUCAUCUCACGAACAGCGCU

>P630

UGGUUAUACAUACUAUUAGGUUUGGACGGUAAGUAUUUCUCACCUAUCGUC

>P631

CUCAGAUUUCUACAACACUAACGGGACAGAAACUUUACAAUCCCACGCAGU

>P632

UUUUCUUCUUCUAUGCCAUCAACAGACACAAGCAAACUGUUUUAACUCCUA

>P633

AAAAGGAUAAAGAUCGACAUUAGGGACUUGAAGAUGGAUCCGAUUUUAAAA

>P634

UACACGGGUUACUUGGAUGUGGAAGACGAGGACAAGCAUUUCUUCUUUUGG

>P635

GGCUAACAGUCCAGUGGCACAUAGGACAUAUGUAACUGAUUAUGAUUAUCA

>P636

AUUUUAAUGCAAAUAUCAACACCGGACUUUACUUUACGUACGCCUUGGCAG

>P637

CAAGGAUGCAAGAUAUUUAUUUCGGACUACUGAUAGCAUUAGAGUCAUUUA

>P638

UACAGGAUCAACUAUCAAUCAAUGGACAGAAAUUUGACAAACGCCGAAGUU

>P639

GAUGAUGGACAUUAUUUCAGGAGAGACGAAAUUUGCUAAUGGUGAGAGUUC

>P640

AUUUCCUAGUAUAUGUCAAACAAGGACAAGAAGGUGAUAUUGAGCCAUUGA

>P641

CUUUCUGUGACCUCCACUGCUCAGGACUUGAGAACAGCAGUAGAUACAGUA

>P642

AGUUCAAUUCGCGAGUACGAAAGAGACAAUUCUACGGUGAAUUCUGCCAAU

>P643

AGAAGAAUUCAAUGUAUUAUACAGGACAAAAUAUAGAACUUUAUUUGCUAA

>P644

AUUUGUGGAAGAGCCUAACUUAUGGACAUCAAGCCAACUAUCUUUAACUAA

>P645

GACUUCGGGGUAGACCCAUCAAUGGACCCAGAACUGGCAAUGGCCUUGCGU

>P646

GGGUGUUCAGGUCAACAGGGCAAAGACAAUUACGGUGGACUUGCCACAUGG

>P647

AUGACGAUGAACUACCAAUAAGGGGACAAAUACCACCUUUUAAGUAUGAAU

>P648

UGAGAUAGAUAUAAGCACCCUCAAGACAAGGUUGUACAAGGACAAUCUUUA

>P649

AGAGAUAAACCAGGGGCCCACUCAGACUUUUACCAUACAAAUUAUUGCCUA

>P650

GUUUACAGAGAUAGUGAGCGAAUAGACUUUGAAAUAAAUGGCAGUUUCAUU

>P651

UCGGUUAUAAACAAGAAUUAAAAAGACAAUUUUCAACAUUACAAGUUUUCG

>P652

AGAAGAGCAAUUAAAUGCUACUAGGACAAUUCAAGCAUAUGGUGGUGAAAA

>P653

GCGUGGAAUAGGAUUAUAUAUAAGGACUUUAAAUCUUAUUUUCCGGAACUG

>P654

UCUUCCAAUGAAAGUACUUAUAAAGACAUUAAAGCCACCGGCAAUGAUGGU

>P655

GAAGAAGAUGAGGACGAGAACAUAGACGAUAGUGAAUUAAUCCAUAGCAUG

>P656

UCUUACCACGUGGAUUUAAAGAUGGACUCAUUGGUAUCCAGUGUGGUGUCC

>P657

GCAAUUCUCAGAAUUGCUUCUACGGACACUCACAAAGCAUAGUAUCGGCAU

>P658

AUCUACUUUGACGAUGGCACGGUAGACAUUACUACCACCACUACCUCCAAG

>P659

GAGCAUGGAUUGAAGCCAAGCAAGGACUUUCCGCACUUGAUUGUUUCGAAC

>P660

AACCGCAGUCAGUUAUUAUCGUGGGACAUUCCAUGGGCGGUAUUGUAUCCA

>P661

GGUCAUCUUCGAUACUAUUUCAAAGACAAUCCAUUUUAUCAAACGUUGACC

>P662

AGUAACAACUUAUUGGCAAACAAGGACAAUUCUAUAACAUCCAAUACUGGU

>P663

UCACAGAGUCCAAAAAUCUACCAAGACUUUCUCUUACAAGAGACCAUCAAC

>P664

ACUGCCCUUACCAAACCCUAGCCAGACCAUCAAUGCUUGAUAGAGAUUUAU

>P665

AAAUAUGGAUAGCCAGCAACUAAGGACAAGGAAAUUCACCAAUGUAAGGCA

>P666

CUAGAAGUUUGGACCGAAAAAAUGGACGAAUUGCUUGUGGAGCUUGGCUUA

>P667

UUGGAAGACCGAAGAAAAUAGAUAGACUUUCGAAUAAGUUCAGCAACAACA

>P668

CUGGGAUUUAUACGGAACAACAAAGACAUUGCGGUUUUCAAUGAUUUGUAC

>P669

UUUAUUGGCAGGAAAUUUAACAAAGACGUUAGAAUGCCGCUAAGUGGAAUA

>P670

GAUGCAAGUUUACAAUAUGUCGAGGACUUGGAUAUUUGGUUGGAGGGCUUA

>P671

GUUGGGCUAUCACUAGGUUAUCCGGACACCAACCAAUCGUUUUCAAGACAG

>P672

CAUGCUCUAUGGGUAUGGAUCCGGGACAAUACGAUGUACUUUGCUAGACUC

>P673

UCAUCACCGUUCUUAUUAGUCCCAGACGCAUGAAAUCACAGGGGAGAGCAG

>P674

AAAUAUGAUCCCUCCCAUCACAUGGACACUUUUAAGCACGUUUUGCUUGAA

>P675

CACCGAAACAAUACGAAAUCGAAGGACAAUUAUUUGCUUUAAAGACCUUUG

>P676

ACGAAACAUCAUUCCCGGAAUAUGGACGAAGAAAAUAAAGAAAAUGUCGCC

>P677

GCAGUAUUGAAGUCGAUACUGGAGGACGAUUGGGGUACCGCCCACUACACA

>P678

ACUAAAACACGGAACCUUAAAUUGGACUUUACCUGAAUCUUCAACUACUAA

>P679

UUGUCUUUGCCUUAUUCAUUCAUGGACUAUCUGAUGGAAUUGCAUUAGGUA

>P680

AAGACGUUGAUCAAGUAUGCCGAGGACCUUUUUGCCAGAGAUGAAAUUGAC

>P681

AAUUUAAGGAAUGAAUUUCCCAGAGACACUUAUUUUAUAUGCACAAAGGUU

>P682

GACUGGAUCAUAUCAAGAUUUGAGGACAAUUCUUUCACUGGUGUGGCCCCU

>P683

AUUUUGGAUGCUAAAAAGGAAAAGGACUAACUUUUCAUCUAAAUGGAUUAA

>P684

AAGGUCAGUUUAGCUUUGCAAAAGGACUUGGAAAACAAACGUGAACAAGAG

>P685

UUGCUAGUUGAAAAGGAUAAUUUGGACAUUAAUACUUUGAGAAAGAUCCAA

>P686

ACAUAGCGCGCGUGCAAACCAAAGGACAAGUGACAAUCUGCACCUGCUUUA

>P687

CAGAUGGAAGGCUAGGCAUAAAUGGACAAUGAAACCAGAAACUCAACUCCU

>P688

UUUGGGUUGGUUUGCUCAGCCACGGACACUUUGACAUCUCAGCCAGUUGCC

>P689

UUCGUGUUUUAUAAAAAACGCAAGGACGUUAGGGAUAUUUUACACUGUACC

>P690

ACAACAUCAAACGCCUCAUUAUUGGACUUUAAUGAGAUGCCUACGUCUCCG

>P691

UAUAAAAAUAUCCCUAUCGACGAGGACCCUGAGAAGCAAAUACGAAGCAUA

>P692

AUCUGGCAGUAGGUCAAGCAAAUGGACUUGUAGAAAUUUAUGACGUAAUGA

>P693

AAAACUACUUUCGCUGUGUACAUAGACGAUCCGUGGAAAGUAACAAAUAAA

>P694

CAACCUCAGCUUUACCUCAAUCUGGACAGUAAGGAAAUUAGCUCGUAUGGU

>P695

AAAAUGAUUUUCACAAAGGUGGGAGACAUGUGAUUGACUAUCUGAACAAAA

>P696

UUUUACAAGGCCCUUUCAGGCAAAGACUAUACUGCAGAAACUGAUCCCGGU

>P697

CAUCAUCCUUUGAGAUCCCAAAAGGACUUUAUCCAGGCGUAUAGUGAUGGA

>P698

CCUCUUAGUACGGAAAAUUUUCCAGACAUAUAGUCUCGAAGAAUUUUUUAU

>P699

CAACAUUUCAGACGAAUUUCAAAGGACAGUUGAAUUGCCAGAAUUGGAAAA

>P700

GAUCUUGCUCAGGGAUCUGUCAAGGACGUUAUCAAGCACUUGAGCAAAACU

>P701

CAAAGCCUAAGUAAAAUGGUUUGGGACACUCCAUUUAGUGACGAAACUAGG

>P702

AAUAUAAAUUCACCGACGAUCAGGGACAAAUGAUCCGCACUGAAGCUUUUG

>P703

GUUUCUGAUGAACAUUUUUUGCGAGACAAGCGAAGAGAGGAUGUACACUAU

>P704

AGAGUACAACAAGAACAUUGAAAAGACUUUGGAACCAUUUGUCUACGAGUU

>P705

AGAAAAGCAUUGCUAGUACCAAUGGACAUUUGCCUAACCCUAAACCUCAAU

>P706

CAAAGACCGUUGGGUCUAGAUAAGGACGUUUUGCUGCAAGCUGCGGAAAAA

>P707

UCAAAAGCUGCCAAAUCUAUUAUAGACAAAGAACUGGAAAAUAUAGAUUCG

>P708

CUCGUAUACGCUUCAAUAGAGAUGGACUCUUGGGAUGGGAUAGACGAAGGA

>P709

UCCAACUGCCUUGCCAGAAGAAAAGACUUUUAUCAAUAACGUAAUCAACGU

>P710

GGUACAUUUGACAUAUCCAUCUUGGACAUUGAAGAUGGCGUGUUUGAGGUU

>P711

UCUUAAGAAGCAUACUUGUAUAUGGACUAGCAGCUUACAUAGAAAUCUAAA

>P712

UUUACAGAGUAGUAGCCAUCAACGGACGCAUAUUGCCUCGCCAUGGAAGUG

>P713

AGGACAAACUUCUACGUCAUCGAGGACGUUUGAUUUGGAAGUGGUACUGCG

>P714

AAUGCCUUAGUCAGAAAAAUCAAGGACACUACAAAAAUUCCCGUGUUGGGU

>P715

UUGUUUGGUGGUGAAAGCUAUAGGGACGAUUCCAUCGAAGCAGAAGAUUAG

>P716

CGAACUCCGGUAGCGAUGCAAACAGACAAUCCAACUCAGGUGCCAAAUUUA

>P717

UGAUAAGAUAUCUUUUGCCGCAGAGACUCUCGAGUUGGCGGUGUCAAUUGA

>P718

GACGACAGAGUUAUGGGUUAUACAGACAUUAGUAUUGCUAACGUUCUACUG

>P719

AAAGUGGGAGCUCAUGACGAUAUGGACAAUGGUGAUGAUUGGUAAUGGAAA

>P720

GCAGACGUAUAUGUCAUUGUUUUAGACGAAUCCUUUCCUGCAAUAAUGUUC

>P721

AUGGAUUCUAAAAUAAGGUUUGUGGACACAAAGGAAAUGAACUUGAAAAAG

>P722

CAAGGACAAUAAGUGGCCGCGAUGGACUGCAAGAGAUACGCAUAUACCAAG

>P723

UGUGGCGAAAAGAAAAAUGCUAUGGACUAUUUUCAAAAAUGUGUCGACAUA

>P724

CUUCACCGUUCUUCAAGCAAAAUGGACUUAGAAAUCAAAGAUAUUUGUCCG

>P725

AACGUUGAGGCCGUGGCAUCCAAGGACAAUAUGUUAAGUAACAAAAUGGAU

>P726

AAUGGAAUAGAGGUCGAACUAUUGGACAGUAAUGAUCGCGUAAACCCACUU

>P727

AGAUGGUUUGACGCUAUGUUUCUGGACGUUUACGAAGAUUUAAGUAUUAGC

>P728

CCAGAAAAAAUAUCGCUAAAUUUGGACUCCAAGAAAGAUACAUCUAAAAAG

>P729

CAAAGAACGGCAGCUAUUACAAAGGACAGUAAAAGCAAACAGCUUUAAAUU

>P730

UACUUCAGAUCAAAAAAACAAAAGGACGCAGUUUUCAUGAGUGCUCAACGA

>P731

UAUGGUUCCUGGACACAAAUAAGAGACGAUCCAUUUCUAGGCAUUACUGAU

>P732

GUUGCUAGUGAAAACCGUGUACUGGACGACUUUAAGAAAGCAUUUCGCGAA

>P733

AUGAUAACGAAAAUGAGCUUCAUGGACAGGGUAGUGAAGAUACCUACAAUU

>P734

GUGACAUGAUAUUCCAAAGGAAAGGACAAUAUACUUUCAAGACUUUAUUUU

>P735

UUCCAUUUUAAAGCCGAACACAAGGACUAGGAUACUGUUAUUUUUAUAGCC

>P736

AUUGGUGUUCAGUCCUUGUACGAAGACGUUGCUCGUGAUACUAAUAGAGGA

>P737

ACGGUACCUGUUGAUUUGGAUUUGGACAUUGUCGAUCAGGUGGAUAUCAGC

>P738

GUAGCUAUUCAACUAAGUAAAAGAGACGUUGUUUAUCCGGCUAGAAUACUG

>P739

UGUUGCUAUGCCUGGUGGAUACGGGACUUUUGAAGAAAUCAUGGAAUGUAU

>P740

CGCGCCGCAGCCGGCCGGAUUACAGACAUUAAUAGAAAAGGUGAAACAAGA

>P741

UGCAAUGUUGCAAAAUCAUGACCGGACGUUAUUUUGAUGGGCAAAAGUUGC

>P742

ACAACGGUUUUGAAUAAAGACAUAGACAAUUCUAAGCCAGAUCCAAGAAAU

>P743

GGCCUCAAGAAAGAAACCUUUAUGGACUAUAGUUGAGAUAAUAUAUAUAUA

>P744

AUAUCAUUUGCACAUGAAUCAAAAGACAUUUUUUUGGAAAGAUUUAUUGAG

>P745

CUCACCUCUGUAUAUGCCGAUAAGGACAUUCAAUACAAUGCCUACAGCUGG

>P746

CAGAUGUUGAUUCAGGGUGUAUGGGACGUUGAUAAUCCGUUGAGACAGAUU

>P747

AUUGAAAACAUUAUCAAUUAUGGGGACUCUAUCCACAAAGAACUUUCUUUU

>P748

UAUUUAAUGGAAGAUACAAAAAAGGACUUGAAAAUCGAGGAGAUUAUAGAU

>P749

UCUGAAUACGACAACAUUAUGAGAGACUUUACAAAAAAAUCGUGGACACAC

>P750

AAGACGUGUUCAUCAUAAUAUACAGACAUUUACAAGGUUAUUUUCCCGAUA

>P751

AAGUUUACAAGAUGCAAACAUCAGGACGAUAAUGUGUACGGGUGAUAACAU

>P752

AGACAAGAUCUGGCAAAAUUAUGAGACGUAUUUUAAGAAAAAUCCUAGCAG

>P753

GCGGGUGUUUGGCUAACAAGUAAGGACAGUUUUUUCACCAGAGAACAAUAC

>P754

ACAAUUGGUCAUAACAAGACCUGGGACAGCUAUUGGUUGCUAUUUUUAUUA

>P755

ACAAAAAAUUCUGAAUGGGGAUGAGACACUCUUUCAUGAAGAAUCCAUAGA

>P756

AAUAACGGUAAUAACAGUAGCAAGGACGAUAUUGGCGACAUAUCAAUGAGC

>P757

ACUCAAAAGCUAGAAUAUUACAAGGACUUAUUAAUUCGGGAAUCUGAACUA

>P758

AGUUUGAAAGAUCAAUACGAACAGGACCAUGAAGACGCCACUAUGGAGAAU

>P759

AAGGAAACACAGAAACUUCACCAAGACAAUGAAAUAGAAUCAAGUAAGGAC

>P760

GCCACAGUUGUGGUCCUAAACAAGGACUGAAAAAGAUCGCAGAAACUAGCA

>P761

GGAAAUCCAGGCAAGACCAAGAAGGACAUGGUUCCAAAGCGAAUCAGAUAA

>P762

CUCAAGGUCACACCAUCUGAUAUAGACUAUGAUUCAAUAGCACCGAAGUUU

>P763

GGGUUGCAUAUUUUUUAAUUUUAGGACAUUAUGGAAAGAGAUUAUUUGAAA

>P764

AAUGAGCUUAUUCUACAGUUAAAGGACUCUGAUAGAUUGCUACAGCAAAAA

>P765

GGAGAAAGCAAGCAUGAUAAUAAGGACAGUUUUGCAGCCAUUGCCAACGAA

>P766

CUCCAAGUAUCAUUGCUUUGUCCAGACAAAACUUGCCACAAUUGGAAGGUA

>P767

UCGGGAAUAGACAGUAUAGUAAGAGACUGCGUGUAUGACAAAAACCAAGAU

>P768

UCAGUGUCCCUGACUGGCAAAAUGGACAGGUCGAAGACUCCAUCCCAUAGC

>P769

UUGCCAAGGAUCAAAAUUCAUGGAGACUAAGCUCAAUAAGACAGUCAAGGG

>P770

UGACGAUGAAUAUGCAACAGAAGAGACUUUGAGCCAUCAUGAUAACAACAA

>P771

UUUAAGUACGAGGACUUUAAGAAGGACAUUUAUAACCAGCUUCACAUGUUU

>P772

ACUUUUCCAUGCAGAACCAUCUGGGACAUUUUACCGUUAUAAUGCCAAAGC

>P773

CUUAUCCAUGAAAAUAAUAUGUUAGACUUUGUUAGCGACGAUCAGGGACUU

>P774

AACAUGGCAGAAUCCGGUAGCAUAGACGAUCUGUUUCAUCAUGUAUCACAU

>P775

UGUCGGUCUGGUUGCUGAUGUCAAGACACUAUCUGCUUCUGGCUACACUUC

>P776

AUGGGUGACAAUUGCUUAAGCGAAGACCAAUUAGCGGCAUUUACUAAGGGU

>P777

AACCUAACAAAAGUGACUCAAAAGGACAUUUGUAUGCUGGGUAUUCUUGAU

>P778

ACUAAGCUUGCUUUUCAUUUUUAGGACUGUUUGGAAUACCCAAGAGGUGUG

>P779

AUCUAAAAUGCAAAAUAUCGCUCAGACUCGUGAAGACUUUGGAAGUAUGCU

>P780

CAUUGAAAUCCUUAACUGAUAAAAGACAAUUAGAAAACGGUGAACCACAAG

>P781

AAAUGGUGGAAAUGUAGAUUUCUGGACAUUUCCAGGAGUGAUGGAUGCGAU

>P782

UAGGCGAUGAAGAUAACAUAAGAAGACAAAUUUUCGAGGAUUUUAAGCCUG

>P783

AAGGCUGAUGCGCCUAUAUGCUUUGACAGGGACUCUUUUAUCAAAGAUUUU

>P784

GUCCAGCAGAGUGACAUUCCCACAGACCCCUUCGAGGGCUGGACCGCGUCG

>P785

GGUAGCGCUGGUACGCUGUUGAUGGACUUCACAGUUUUUAUUCAAUUUUUC

>P786

GUGAAUGACGAGAUUCUUAUAGAGGACUAUAAAUUAAGGAAAAGAUUAUGG

>P787

CAACUAUACAACCUCGUUGCCAAGGACUACGCUCUCACGGACACCAUUGAG

>P788

UGCUGUAUAAUGAAGUUUAUGAGGGACCUACUUUAACAGAAUUUGAGGAUG

>P789

AAUCUUCUUUCCAAAGCGCUUCAAGACACGAAGCACAGCUCGAAGACACUC

>P790

GAAGUGGUUGUGAGAAUUAGUAAGGACGAUCUUAAAACAAGUGCUUCCGUU

>P791

GAUUUUUGAACAAUGGCUCCUACAGACAAUUAGAUUUCAUCAAUGUGAACC

>P792

UAUUCAUGCCGCACUGGUGAUAUGGACAAUGUUGAUAGAUUGAUUUCCACG

>P793

UAAUUAAGGAUUUUGAUACUAAUGGACUAGAACUCGAUAGAGAAAGGAUAA

>P794

UAUGGAAAUUUUUCAGGUCAUACAGACAUUCCCUAAGACAUUAUUGAACCC

>P795

GAAACACUGCCGGCAUUGGGUCAGGACGGCAUUAACGAAGACAAUCUGAAU

>P796

GAAGGCUGCGAGAUCAGUUGGAAGGACAACGCCCACAACGUCACUGUGGAC

>P797

GGCAAGUGCUAUCACUCAACAAAGGACAUAUCACCAAUCACCCUUCAUGGA

>P798

AAUUUCUAUACUUCAACAAAACAAGACAUUCUUGAAAGGCAUCGACAACAA

>P799

ACAACCGUAAAUAACCCAGAUCAAGACAAGGCAAUGCAGUACAGACAAUUG

>P800

AGGUGGAAAAACCAAGGGAAAGAGGACAGAUUAUAUACUAUAUAGCAAGAU

>P801

GAACUCUAUUGAUGUUGUAUGGGGGACGAUGGCAGAUUGCCAGGAUCACUU

>P802

ACAUAUUCUCUGCAUGAACCAACAGACGCUGUGCAUGCCAAUAUAGAUACA

>P803

GAGCAAAAUCAAUUACUAUUGGAAGACAACCUCAAACAGAUUGAUGAUAGG

>P804

GCCUUGAAGAAGGCUGACCACCAAGACGACGGCGGCAUCAAGGACUACGGU

>P805

UACUUGAUAACUUUAGCAUAUUUGGACAAACUGCAUCGCGAAAAAAAUCAA

>P806

AAUCAAAAUAGAAUGAAAGUAAAAGACAGACCAAGAGGCAAAGAUGCAAGA

>P807

UGAAAAUGUCAAUUUCCGUUUAAGGACAACUGCAUCAAUAAUAAAUGGGAU

>P808

AAAGCCAAAAAGAGAAGGACAAUAGACCCUAAUAGACUCAUAUGGAAACCA

>P809

AAAUCCACAACAGAAAAAGGCACAGACGAAAAAUAGUGUGGAGCUUGUAAG

>P810

CAAAAAUGGACAUGUCUACACAAAGACCGUCACCCAGGACGCUACUUUCGU

>P811

AAACGGUUACGAUACAAACAUCAAGACUUUAAUCAAUGAUGGUGUACUGAA

>P812

GCAGACUAUAGCUGUAAACCACAGGACUUUGAAAGGAAAACGAUAUUGGGG

>P813

ACUUAACUACACCACCCAGGUCUGGACACUUCCCAUUAGAGCCGGUUGAUG

>P814

AACCGGGAAUAUUACGGAUACAAAGACAGCAACGUUCAAAAAAAUAACCAA

>P815

AGAUAUCUCCUGGUGAAAUCUCUGGACGGUGUAGUGAAAAUAUGGGAUUGC

>P816

UGUGUUUACGGGAUCCAGCGAUAAGACGUGUCGAAUGUGGGACGUUUCUAC

>P817

AGCUAGAUAAAACUGAUUCUCAAAGACUAAAAGAUCAAAUUUCAUCCUUAA

>P818

GUUGUUUUAACUACCGUUGAUAGGGACGAUUUAGUCGAUGGUGGUGCUAAU

>P819

UCAUACAAACCCUCAACCUACUGAGACAAGUGAAGCCCAAAAUCAAAACCG

>P820

GCAGCACAAACCCUACUUCAAAUGGACUACAAGGAUGAUAUGAAAGUCGAU

>P821

CAAUUUUGACCACGACGACGACUGGACUUUAGACAUAGAGAAUGACAAAAA

>P822

CACGGGUGCGCAGUAAUCAUGACAGACUCUAAAAUGAAGCAAACAUAUGAA

>P823

GAAGUGGAAAACAUAACGAUUAGGGACACAUCAUUGGUCUACCUUCCACAA

>P824

AGAAGUUUACGACCACUACCAAAAGACAAUUUUAAAGCCAGGUGUCGAAGC

>P825

CUGUUGUUGGGCUAAGACAACAAGGACUUAAGAUGGACAGACUAUAUGAAA

>P826

AAAGUUCCGGAUCAGAUAUAUAAGGACUAUCUUUUGCACUCAUUUGUCGGG

>P827

GAAUGGGUAGCUUCAAAAAAUAUAGACAUUUCAAAACCAGGCGCGGAUGUU

>P828

GGGGUGAUCUUCAAUAAUGAAAUGGACGAUUUCGCUCAAUUCAACAAAUCU

>P829

CUGAAAAGGCCACAAUGAGGCCUGGACUUACGUUGAUAGGGAGACAGAGUG

>P830

AAACCACUGUCACCUGGUUGGACGGACCAAACUGCGUAUAACGCGUUUGGA

>P831

UUACAACACAACAUAACGUUGAAAGACAGUAUUAAAGUCAAAAAAAAUGGC

>P832

AGGAAGAGUUAGAAAUGUAUUGAAGACAUUAGCUUUGAGAAUUUGUGCCGC

>P833

GAACAAUUGUACUCAAAACUAAUGGACCUACCGCAAGAGAUGCAACAAAUA

>P834

AUUGAAGCAAUGGUCAAAGCAAGGGACAAUAUGGCCAUACAAUUGGAAGGU

>P835

GUUGAAGAAGCCCACAAGUUUAUGGACUGUAUCAACUUCUGGGCAGGUAGG

>P836

AAUGACACAAACGAGAUUAAAACGGACAUUAAGAAAGGCAAAUCCCUUGAA

>P837

CAAACACAUAAUAGCAAUUACGAAGACACCAAUACCAACGAAGGUGAAAAU

>P838

UGAAGAAGAUAUUUGGCAAAUUUGGACUAAAGGGUUUUUAUCGAGGACUGG

>P839

CUCCGGAUCGACAACACAGAAAAGGACAGAAUUAGACAGAUUUUUAAUCAG

>P840

UACAUAUAUGAUAUUCGCAAAUGGGACACUCCAUUGAGAUCACUAAUUGAC

>P841

UAUAGAAUGAGUGGGAUCGUGAAGGACUUUUCUGAAACUGUUGUUACGCAG

>P842

AGAAUCGGUGUAAAUAGGCUAAAAGACUACUUAAAGCCAUUAGUGGCCAAG

>P843

GGCCAAGAAGACAGCUUCAAUAAGGACACUAACGUUGUCAAAAGCGAAAAU

>P844

GAUGUAUCAUCACAAACAAUUAAGGACAAUAACAAUACUAAUACCAACACC

>P845

AGGGAGCCUGUGGUGGAUCAAAUGGACUCAAAAAAGGGGCACGAUUUUACU

>P846

AACCCUGAAGAAAAAGGUGCUUUAGACAUUGGUAUAAACUUGGCUGAAAAA

>P847

AGAUUGAACAUUUUAACACAAGAAGACAAUACGGUAAUGGUAUCCCAAAUU

>P848

AAAAUUUUGCACAUACGAGAAAUAGACAUUUUAUUGUCCAUCGCCGGCCUG

>P849

AUGAUUGGUGAAAUUAUCCCUAUAGACGUUAAGAUUGACCACUAUAAGCCU

>P850

ACUUUAUUAAGAGACUUCAAAAAGGACAAUAAAUUCAUUGAAGAGGAGUUA

>P851

GUCAGGGAUUUAUUGUGUCGCCAAGACCGAGAAUGACUGGAUUAGAGGAAU

>P852

UGCCUCCGUUUCUGGUGCCAUAUGGACACAAACCAUGCCCAACCAACUCUA

>P853

AAAUACAGCAAGUGAUAAUCAAAGGACAAUGAAAUAUCUGCAGGAACAGCU

>P854

CAAGUUUAUCUUGCAAAAAAAAAAGACAGUGAUGAAAUUUGUGCUUUGAAA

>P855

ACCACCGUUCCAUGCCCAUGGUUGGACGGUAAGCAUGUUGUCUUUGGUGAA

>P856

AUUUCCCCAUGGGUGAUAUUUGCGGACUAGAUUACACUUAUGCAUCUGACA

>P857

GACAUGACACAGAGUCGAAACAAGGACGUUUCAAAUGUUUCACAGAUACAU

>P858

AUAUUCAGCGAAAACAAUGACAUGGACAAUAAUAACGAUGGAGUAGAUGAG

>P859

UGAGUCGCAGCAGUAUCUAGACAAGACAAGUACGGAAAGAUUAGACAUUUU

>P860

ACACCGAUGAUGAAAUAUACAAUAGACAAUGAAGAAAAUAAUGAUAGAUAG

>P861

GUUGGAAAACCAACUUUGAGAAUAGACAGUAUUACACACAAUUUGAUUAGU

>P862

CUUGCUGGCAGAAAGAACUCCAGAGACGAUGAAUGAACAAUAUCUAUUAUG

>P863

ACUAUUUAAUUUCAAGAUUACUUAGACUUUGUCAUUCAAAUCGCCCUUUUA

>P864

UCAGAAACACUAAAUUUUAGAUCAGACAUUAUGAAAACUUUGGAGCUUCCC

>P865

GAUCUCUUCAAUGAAAGGCAAUUAGACACUUUCAUUUAUGCCGUCUGGGAU

>P866

ACAUUUGAUAAAGUACUUCAAGGAGACGUUAUAUAAGCUGGCCAAUGAAUA

>P867

ACUUCAGACCCCUGAUUUCAAUAGGACAAUAAGCAAUGAGCCAUUGACCAC

>P868

UGACUUUGAAAACACUUUCCAAAAGACUGUUGAACCUGUUUAUCAAAUGCA

>P869

UAUAUUAAUAUAAUAUCUGAAAGGGACGUAUUAAAGGAACACAACGCAUAU

>P870

AAAACAAAUAAUGUUACUUUGGAGGACUUUUCCAAGAGGCAUUUAAACAAA

>P871

GAGAGAAUAUCGUCAUUUGAAAUGGACUUCGUUAAAAUGCUAUUGUGCUAC

>P872

CACCUAAAUUCAGAACAGAUUAUGGACGUUUCAUUGGCUGAAAAUAGUUAA

>P873

AACAAAAUCUGGCUUCAAGAAAUGGACAGUAAGUGGAAAAUGAAUGGUCAU

>P874

AUUUGGAAUGAAGGUGUUGUGAUGGACGAUAUGGAUGAUGUGACGUUUUUU

>P875

UGUUUAGAACAAGCCAAGGCGGUAGACCUGUCACUCAAAAUUCCAUAUCUU

>P876

CUCCUUAACUGCAAAAUAUACAAAGACACUUAGCGAGUUGACAGAAAAAGU

>P877

GAUGUUGUGGAGCAAAAUGAAAUGGACAAUGAAUCUAACGUUGACGAAGAU

>P878

UAUGCCAAGUACGCCAUCGGUAAGGACGCUGCUGCUAUGAAGGCCGUUGUC

>P879

AAAGCUGAUGUGCCAGCUUUUAAGGACUUUAAGCUGGAGGACUCAGGUUCU

>P880

UUGGAUAUUGUGUGGUCAAUUAUAGACAUUUUUAAGGAGUUUUGUAAGGUG

>P881

AACGCUACCACUGAAAACUCUAAGGACAAACAGAACGAAUUUUUCAAAAAG

>P882

AGAAAUGCAGUGGUCCAAGGUAUAGACUCUUCCUUGAAGAUUGACGUUGAA

>P883

GGCUUCAAAUCGACCACCGUGAUGGACAUUCUGGAGAAGAAUUAUAAGACA

>P884

ACCGUACAGAGACCGCAAUGUAAGGACAUUUAUAAUGUUUACCACGUCUGU

>P885

AACCAGAUGGGAUUUUGCCUAAUGGACUGAACGUCAUCAAAUUUCAAGCAU

>P886

GAUGGACAACAACGAAAAAAGAAGGACAAUCCUCGUUGAAACCGUGGCAAA

>P887

GAUUCCAGACAUAGUCUCAAUCCGGACACUUCAGACGUUAUUUCUAAUAGG

>P888

ACCAAGAACUGGCAACGAAUUAUGGACUCUGGCAACUUUUUGCCACCAAGG

>P889

UAAACGACAUGCGAGUCAAUUAAAGACAUUUUUCUAGCUACAUAAUCCUUA

>P890

AUUCAGAAAUUGAAAUGCUUAAAGGACAUUUACAGUAGAUGAAUGAUGUAU

>P891

UGUUCAAGCACCGACGGAUGAUAAGACUCUCAAGCCAACUUUUUCUUUUAC

>P892

UGUUUAAUUUUGCGAUAAUUAAAGGACAAUUCGCUCGUAUUCACUUAGGUU

>P893

CAAGACUCUCUAGCUUUGCUAAUAGACUUUAAAGAUUACCCUGAUGUUCCU

>P894

CCACUGGUGCAAAUUAAAUUCGAAGACACUAACACUGAGAUCCCGGUUAGU

>P895

GCAUCAUUGAUCAUGCCAUUUUUGGACAAUCAGGUUAAUUUCAAAAAUCAA

>P896

GAUAGAGACCCCAGAACUAAAAGAGACGAUAUUCACUACUUUAAAUAUCCU

>P897

CAAACAAAUUAAAGAAGAAGUUGAGACUUUUAUAGUAUUUAUUAUUUAAUC

>P898

CGGUGUUGCGUCAAGGGCUGAAAAGACAAUCAAUUACUACAAAGGAAAGCA

>P899

AAGAACGCUUCAUUUAAUGACGCAGACUUAUUGUCGUCGUCGUACUGGAUA

>P900

AUACGGAUUCAACGAAAAACAGCGGACAGGCCAUUCUUUAUGAGACCGUGA

>P901

GGAGAGGGAUCUGGCAAUCACAAGGACAAUCGCUGCAACAAACUACUUUGG

>P902

UUGCUGGAAGAAAAAGGCCUAAAGGACGUCAAAGUGGAAACCAUCGAUGCU

>P903

AAUUUCUGGGAAAAAAGAUGGAAGGACGGCUGGGACGAAUAAGUACAAUCU

>P904

AAAAAACACUUCAACAGUGCCAAAGACUCUACAAAAAAUAUUAGAUCUACU

>P905

GGCUUUUACAAUCAUUUUGGUAAAGACAUUACAUUCUGGUGUACUAGCUUG

>P906

CAGAAUAAGUUUGUAAUUGUUAUGGACGAUGCCGGUCGUGAAAAUGAAGGU

>P907

AAAUCCGAACGGAGAUUUGUCCAGGACUGAAUCUAAAUUCAAGGGUAUGUU

>P908

GAUGGAGGGCAACCACACCAAUUGGACAUUAUGUUUAGUAAGAGAAUGGAC

>P909

AAAAAAGCGUCUAAUUCCAAAGAGGACAUUUCCAUUAACGAUUUGAUUUGU

>P910

UCCCUCCAUCCUGGGUUUGACGGGGACGUUCGAUGAGGUGAAGAACGCAUG

>P911

UUUUACCAACAAGUGCACAAGAAGGACAGAUUACAAUAUUUGCCAUUAUAU

>P912

UACGAUGUACCCAAAACGUCAAUAGACCUGAUACACCGAGUGGGCAGAACU

>P913

AUGAGUCUAUCUCAGUCAUGAAAGGACAAGUGGAAGAAUCGGAGAACGCUA

>P914

GAUAACAUCUAAAGACGAACUAAGGACUACUAAAUCGGAUUUUGACUUCGU

>P915

ACACAUCUAACAUGAAUGCAGGCGGACUUUUUGGUGCAAAGCCUCAGAAUA

>P916

GCUGACCUGACAUUUGUUAAAAAGGACGUAAGAUCGCUAUCGAAGUCAAGU

>P917

UUCAAGGAAUGGGGAGUUGGUUAAGACAUUACAGGCCAGUUGCAAUGGAUA

>P918

GUCAUUGCAGUAGCCAAUACAAUGGACUUACCAGAACGUCAGCUAGGCAAU

>P919

GAGGGCGUCGCGGAAGAGUUUGUGGACUUUGAUGAACCAAUAAGGCAAAAC

>P920

GAUACUGAUGACAUCUUCCAAAAGGACUUUGGUGUCAAGACCACUUUGCCA

>P921

AUCAAAAACGGACAUUACUUGCCAGACGCGAAUGAUAUGGAUAACAAUCAC

>P922

ACAAGGAUUUUAUUGACUUAAUGAGACUAUAUUUAACUAUUCAUUCUGAUC

>P923

UCAGAUCUCUUUAAUGAUUGUGCAGACCCAUUAGAUUACUACGAAAUAAAA

>P924

CUUUACAACGGCGUCGAUAAACUGGACGAUCCAUAUGACGACGAAACGUUC

>P925

GCUCUCAACACCAACAUCCAAAUGGACAGAACAAUGGAAACAAUAAUAGCA

>P926

UGCGAUUCUAGAAUACUGCCAAUGGACAAUAGUUUUUAUCAAUCAUUUAUG

>P927

UCGGGUCACUAUGAUCAAGAAACGGACUAUGUUUGGGGUCGCGGCUCCAAU

>P928

GGAUAGACUAAAUGCUUUAAAUAGGACUUUAAAAGGAAUGAUUAUCGGAAA

>P929

AAAAAAAUAACGUUACAGCUGCUGGACACUUCAAAUCAAAGAUUCAAGGAU

>P930

UCCUUCCAGAGUUUACUGGUAACGGACCAAACUUGGACAAUUUAAAGUAUU

>P931

UAAACCAUUUAACUCAGAAAAAGGGACCAUUCAACCAAUAAACAUAAUGGA

>P932

GGGUUAAUUGCCUCCGCAUCUAAGGACAAUUUAGUAAAACUGUGGGAUCCG

>P933

CUCUUUCCUCAUCUCCUUCUUUUGGACAACAAAAUGACAAUAGCACCAACG

>P934

UUUGAACUGGAAAGAAAACUAAAAGACGUUUGCAAUAAGAUUCUAAACGAU

>P935

GUUGGUGCUGAUUUAUGGGUAAUGGACACUAAUGGCGACACGCCGCUGCAU

>P936

AAAGAAAAUAGAGAUUCCCUCAAGGACGAACUGCUAGCUUUAAAAAAAAAA

>P937

UUUAUACACAGUGGCAAGACUGAAGACACCAAGGAACCGAGUCAAGGGUGU

>P938

GUACUCCCAAAUGUUGAGGGUAAGGACUCUUUAACAAAAAUUGCUAGAUUU

>P939

UAAUCGGUAUGAAUGACCCACCAAGACCGAACGUUAAAUUUGCAAUCGAAC

>P940

AAGGAAAAUAUAACAGCUAUUGUGGACAAUAAAUCAUCAUAUUAUGAUAAG

>P941

GAUACUAUUGAAUUGGACUUCCCGGACAUUAGCUUUAAUCAUAAGUGGUCC

>P942

UGUUCUACCCAGCUAUCUCACACGGACGUGUACGGUAAUCUUUUGUGGUUG

>P943

GAACUGCAUUUGUCAUCUCCCAAGGACUACCAUUAUACUAACCAAGGUGGA

>P944

GGUUCACAUUAAAGAAUACUCAGAGACUUUUGCUCAGCCGUAUUAGUAAAU

>P945

AAAAAGCAUUAUAAUCGCGAAAAAGACAAAACUAUGGAUUGGCACAUCAUC

>P946

GAAAACUCAUCCCACUAUAGCAAGGACGUUUGCGUUUGGUGCAACUGAUGG

>P947

CGAAUUGGCAAACUAAUGAUAUUGGACAGCAACUUAAUCUGGCAAUGUUUC

>P948

GAGAAUUCAAAUGUAUUCAAAAAGGACGGUGAAUCCAUCGAAGAUGGCGCA

>P949

GGCGGCUCCUCUAAUACUAUAAAGGACUUGGAAGAAGCGUUGGAUAGCAUG

>P950

ACGACACCAACACCCCUGACGUUGGACAAUUGAAUUUGCUAUUUAUCAAGG

>P951

UUCAAGAUCCUGCAACGUGCCACGGACUCUCAAUUGGCCACGCGGAUAGUU

>P952

GGAUUAAGUAACGCAUUUCCAACGGACUAAGAGCUGAUUUCUGGCUCAUCU

>P953

AGGGAAAUUGGGCUUCUGCCAGAGGACGCUUUAGUAAAUGAUUUUAGCGAC

>P954

AACGGGUUGCUUCAGGUGCUCAUGGACAUUAACACUCUGAACGGAGGGAGC

>P955

UGAGCUAUACAUAGAUAAUGUGAGGACGCUUCAUUUAUCAAAUCAGGAUGU

>P956

UUGCGAGACAAAUUCAUAACUAGGGACGAAUCAACAGCAACUUACAAGUAC

>P957

UGUAUAUUAACUAAAAGAACAAUGGACAGGCUCCCAAGAAAAAAGAAAUUU

>P958

UAUUGGGUGGGGGUGUAUGAUAAGGACAGGACAAAGUUUGUUGGGGAAUGC

>P959

AACGCAUUUGAAACAUAUGGCGUAGACUUUUUGAUUGAUUCGAAUUACGAA

>P960

GAGGGGCAUAAUAUAAAUGAAACGGACAUUUUAAGUGAAUACUCACCAAGG

>P961

UGCUUGACAGCAACAUGUGAGGAGGACAUUACAGAUUUAAUGAAAAACUAC

>P962

UUUGUCUAUCGGAAGACAAACAGGGACUGUGGAUUUGCUAGAUCCAACAUC

>P963

UGACUUUUUUAUCAUUCUACAAAAGACCAGUAACGAAAUGUACCCUGUUUU

>P964

AACUAAAGAAAAAGAAUAAAACUGGACUUUUAACGAAUGACUUUGAUGAUA

>P965

AAACAGCUCUCAAAAAAAUUUGGAGACUACUGACAGUUACCUAUACAUCGA

>P966

GAGCCUGAGACAAAGCAAUUGAAGGACAUAAAAAACAGGCUUAAUGGUUUG

>P967

GUUUGACUUCGAUUCCGAAACCUGGACUAAAAUAGACCUUUAUGCCAAAAC

>P968

UUUUACCGCUACAGGUCAGGUCAAGACGCCGAUGGAGGUAUAAUUGCUGGU

>P969

AUAGAGCUGUUUGCUAAUGGCAAGGACGAAGCCAACCAGGCGCUCUUACAA

>P970

ACUACUUUGAGCUUAGUUCAAAAGGACAUUAAUUUAUUGAAGAAUGGGAAG

>P971

UCGCAAAAUCAGCGUCUCUAAAAAGACUUUGAAAAGUAACUGGUAGCGAUA

>P972

CCUACUAAAAAAGAAAUGACAAUAGACUUUUAGGGCUUUAAGAAGAGUCCA

>P973

AAGCGAAAUCUUUUAGAAAUUUUGGACUUUAGUUGAAGAUUAAUGUGAGAA

>P974

CGGUACUGUCUUGCCAUGUAACUGGACUCCAGGUGCUGCUACCAUCAAGCC

>P975

UAUUACUAUCCCCGAGCAAACUCAGACUUUUGAAUUCGCGUCGCGUCGCGC

>P976

UAUUGAAGAUUACACUGUUUUGAAGACCAUCACAAUUGGGAAUUUGCCAAA

>P977

CGAUUCGUGGAAGAACGUUACAUGGACACACCUUAUCACUAUGCGUCUGAG

>P978

AGUAAUUUAAGCUUUAAGGACUUAGACAUUGAAGAGAGAAAGAGAUUGUUG

>P979

UGUAAUUGAUAAACACUAUAGAGGGACAAACAUACGCUUCACGAAGAACGA

>P980

UACCUCUAGAGGGUUGUAUAGAUAGACACAAAUUUUGACAAUCACAAAGAA

>P981

AGAUUUAUUAGAUGAUUUGGAAAAGACAUUGGAUAAAAAGGAUUCCAUUCC

>P982

ACAUGGAGAACUGGGCGAUUGCAGGACAGUUGUUGUUCACCUACAAAGAUU

>P983

CCAUAUAAAGCACAGACCGAACAGGACUUUAAUUUUCAACUUUAAUAAUAA

>P984

GAGCUCGAAAAAAAAGCAAACAGAGACAUUGAUUUUCUGCAAGAAAGGGGA

>P985

CUUUGGAUGUUAGAAACUUCUCUGGACGAUGAUGAUUUUACGAAUGCCUAU

>P986

CAAAGUGAAUAUGGAAGUGCGAAGGACUCUAGUAACAAUACAGGCCACAAC

>P987

GGUUUUUGCGAAUGCCAGCAUCAAGACAGAUGGAAACCGAAGCUGAUUACA

>P988

GAAGUUACGUAUAUCGGGCAUAAAGACUUUGUAUUGUCCGUGGCCACCACA

>P989

CGUCAAUUUUCGCGCUAAUGCAAGGACAAUUAUCGAUUAUACCCAGGUUUU

>P990

CAGAAAAAAUCUUUUCAGACUAUGGACUGUUAUCACUAUCGAGACAAGAUG

>P991

UGAUUAUAUCUUCUCCAUCUGAUGGACAAGUUAAAGAAGUGUUUGUCUCUG

>P992

UCCACUCUUUUUGAUGAUUACAAAGACAAUUAAUGCCAAGUCGAGGCAAUC

>P993

AGAGUUUCGCUGCCUCGAUAGCAAGACAUGAACAUUAUUUAGCUUACAAGG

>P994

GUUAUGAUUUAGUAUUCAAAAAGGGACAAUAUGGAAGACUACUUUAUUCGA

>P995

AUUAAAUUAGCAUUUUUCAAAAGAGACUUUUUUAAAGGAUUAUAGAUGAAA

>P996

CGUUCGUUCUGUGAUGAACAUCUGGACUAAAAAAGUUGGUUUCCCAGUCAU

>P997

GUGACACUGCGACUCAAGGCACCGGACGCUUAGAUUCCAUGUCCAACUUUG

>P998

AAAAGCCAUUCAACGAUUUAUACGGACGGUGAUUUUGAUUAGUCUAAUUGC

>P999

AAGGGUACGUUUUGUCCCCAGGUGGACGUUUUGGUGGGAAGUUUAUAGCAU

>P1000

UUGUUCACCCAUGCUCCAGUAUAGGACAAUUUUUUAUUCUCAGAUUCCUGA

>P1001

GAAAACCGUACCAGAAUGCAUAUGGACAUUUUCAUGAUACUUCUUUGUACG

>P1002

GGAAUCAUGUCCCCUCCAGCAAAGGACACCGCUGGGAGGAAGAAAGCAGAA

>P1003

ACCCGUUGGACUUGAUCAAAACAAGACUAUCGAUUCAGACAGCAAAUUUGA

>P1004

AACGGGGCCAUCCUGAGGCGGUAGGACAAUAAGUUAAAAGAAAGGUUACCU

>P1005

AGCUCAUAUACUAGCCUUUGCAAAGACUUUGAAGCAUGGUUUAAUUCCAAA

>P1006

UCCCAAUUAAAAAAGAAAUUCAUGGACUGGGAUAGUAAUUCCUGGACAGAU

>P1007

UUAUUGCCAAUGCUGUCAUUCAUGGACUAAAAGAACUCAAAGAGCAGCAUA

>P1008

UUGCAAGUAGAAAGUAGCGCAUUGGACCUUUUAGCCACGAUGGGUACAGAA

>P1009

UAUCCAGCUUUGUAAUGGUUUUAAGACGCUUCUAAAAUCUAACAGUAGUAA

>P1010

AGAGUCCACCGCCUUUUAUUAAUAGACGCGCUCUAUAUGUUUAUACAAAUG

>P1011

UAUGUCACUUCUGCUGGCGCAAAGGACUUCCAAAAAUGAUUCACAGCAGUU

>P1012

CUUACAGCCGUUCCCGUCCCACAAGACCGCUGGGUUAUCUCCCAGUAUGGG

>P1013

UAAACGGUGUGGUUGCUUUACAAAGACUAUGUAAGAAAUACCCAGAAAUAU

>P1014

CUUCUGAAACAAAUGCAAAGCCAGGACAACCUACCACUAAUAUUCAAAGAG

>P1015

GAUGGAACUGAGCAAAAUUUCAAGGACAUUGUCAUAUUUUUGCUGCAAAUG

>P1016

AAAAUAUCGCUAAGUGAUUUAGAAGACGUUUUAUUAGAUAUAGAAGAAUAU

>P1017

AAAUCACCGCUUAUGGUAAAGAUAGACUUUUGAACUAUGGUGUCGAUGACG

>P1018

CAUCCGUUCUGGAGAAUAUUUUUGGACACUUCAUCGUGCCAUUGUUAUCGC

>P1019

AUCCUACUACAUCUAAUUCACAAGGACCUGAUAAAGGUACACAGUAUCGCA

>P1020

CACAGAACACGCCGAUGAUCAAAAGACAAUGUGAAAUAAUUCAGUUCUAAC

>P1021

UACUAGCAAUGGCUCACCGAGAAGGACACAGGGAUUUGGACACUAAAGUGA

>P1022

GAUCCUACUUACAGAAAUCGCAAGGACAUAAACUGGCAGUCGCUUAAAUCC

>P1023

CGAUUUACCAGAAACCGCUGGCAGGACUUUUAUUGAGAUAAAUGAAUUGUU

>P1024

GCUACAGGAAGAAAUGUACAUUAAGACAUCCGCCAGGAAAUGAAAUCUACA

>P1025

AUUCAGGGACAUUAGAACCAUGCAGACAAUGAUCCAAAAGGAUAAGAACUU

>P1026

ACUGGACGAUUUAGUAACGAAAUGGACUAACCAACUAACAGAAUCGGCCUC

>P1027

GCGCACGACGAUGAGGCUUCCACAGACGUUGAAGGCUCCACAGACGUCAAU

>P1028

CUUGAAGGAAGUUGCACUACAUAAGACGGUUUCCGCUGGUUGUUCACAAAU

>P1029

AGAACAUUCCGGUAUCUCUUGUUGGACACAUCUAAUGUAUAUGAUGUUUUC

>P1030

ACUUUUAGGCAGAAUAAUUCAAUAGACUUUCCACUACAAAACAUAAUACCC

>P1031

AGCACCGAGGAAGCUGCCGACGAAGACGAAAAGAUCAGCGAAAUCGUCGAC

>P1032

ACCAAACAAUGUUGAUACAACAAAGACUAAUUUGCAAGUGUAUACCGAGUU

>P1033

CAAGAGAUCGUUGUUUCAGAGGAGGACAGUGAAAAAAAUAACAAGGAUGGA

>P1034

AAAGGCAUGAAGGGAAAACUCAUGGACUACUAGUUGCAUGGAACAAUAAAA

>P1035

CCUAACUUAUACUACCAUUUAAAGGACAAGUUGAAAUUAAAUGUGAUAAUG

>P1036

UUAGCAGGUUUGAAGAUCAUGUCGGACAGAAAUAACUUAAUGGUUGCUGAC

>P1037

UGAAGCCGAGCGACCAGUUUGAAGGACCUUCCAAACAAUUACAUUUAUACG

>P1038

UAUACAAAACCUGUAACUCUAGAAGACUUUCAAUCAGACCCAGAAAGACAU

>P1039

ACCCCCAUCGAUUACGAUAUAUUGGACCAGAUAUUAACUCCGCUAUUUACA

>P1040

ACACUAAAUGAUCACCAAAGAGAGGACUUGAUUGAUAAAUUAAACAUGACC

>P1041

GAAGUUUAACCGUAUGAACCUACAGACGACGCGGUUUAGAGAACCGCAGAU

>P1042

CUUGGAAGCUAGAGUUGGGACAAGGACAAUACCAUAGUAUAGAACAUUAUG

>P1043

ACAACCUUAUCAAGGCAGCCGAGAGACAAUAUAAUGGCGAGGCUUCGUCCG

>P1044

UCAAGAGAGAGCUUUAAAGUGAAGGACUGCGCUUAUUCUUCUUACUUUGAA

>P1045

AGAUAUCUCGGAUUUCCUGACAGAGACAAUAAAAGGGCCAGGAGAUUUCAG

>P1046

AUUGCAAGAUGAUUUUAGUUACGAGACAUUUGAAACAGAUUUGGGUGGGAC

>P1047

ACAAAAUGAACCACAGAUAUCUCGGACUAACUCGGGUACCUCAGAUUUUAC

>P1048

ACCGCCGGUUCCAAUUCUGAUAAGGACUAUUCGCAAGCAAAACUUUUGGAA

>P1049

CCGAUUGCAGCACCAAUGAAAAUAGACAUUUAAUCAAAGAUGCCUGUCAAA

>P1050

AUCAAUGGAACCCCUUUCCCCACAGACAUUAUUGAUAAGAUGUUUUACAAA

>P1051

UUAAAUCAACCAAAUUCAUCAGAAGACUUUUGGUGUUUUACAGACCACUCA

>P1052

CUCUUCGUGAAUUGAAACUACUAAGACAUUUAAGAGGGCACCCAAAUAUAG

>P1053

UCUGAGGUCAACAAGGCAAGCGUAGACAAUAAAGCAGCCCAAAGUCCAGAA

>P1054

GAAGACUAAACGUCGCCAUGACAAGACCCAGAAGGCAACUAGUUGUUGUUG

>P1055

AUACGUACAGAUAAUUCACAACAGGACAGUAAUAAUAGACGCGAUAUUGUG

>P1056

CAAAGUUUACUUGGCUGGGGCACGGACGUGUACAUCAAGCUCAAAGGCCCU

>P1057

CUUUUUGGGCAGCUUGUUAUUAUGGACCCAGGAUGUUUGCCAGAAGAAACU

>P1058

CAUUCGUCGUCACUCAAGUGUAAAGACGACAGAGAAUGCUGAAAAAGUAUC

>P1059

UGCCAAAGCUAAGGCUCGCGAAUGGACGAAAUUGUAUGCAAAGAAGAAACC

>P1060

ACAAUGCCAAGAUUCAUAAAUAUAGACGGAUUAUCAGGGAAGGUACGGAAA

>P1061

CCGGCAACAGGAACAAUAUCUCCGGACUCUACCAAGUCAUCUUCUUCAAGU

>P1062

UUGCAUCCAGCCGAUAGCAAAUUGGACUCUUCAAAAAGAAUUUCCCUUAGU

>P1063

GGGAUUAAUGUCUCAGUCGAUCAAGACUUCUCAAGAACACAACGACCAUUA

>P1064

ACAAUCUUAACACUGGAUCAUAUAGACGAUGAAUCUCGGAGAGAGGAGGAU

>P1065

GCUUUACUUGAUGAUCACUGUUGGGACCAGCUAUCUCACUUUCAAUGAAGC

>P1066

AUUGUUGUAAAUACAGACUUUUCGGACAAGAAAAACAAAAUUACUCAAAUC

>P1067

CGGGAAAAGCCGAGCUUUAGUGAAGACGUAAAGGAAGAAGAAAGCAAAGUA

>P1068

AUUGAGAACAUUGACUCAAAAUUGGACGAUAUAGAAAAGGAUUUGAGGGCA

>P1069

CGUUAGGCGAAUGCUACACAACUGGACACGAAUACAAACCUGAGAGUUCUG

>P1070

UCGAAUUUGCCACCUCUACCGAUGGACGCACAGGAACAAUUAAAUGCAGGA

>P1071

AUUAAGCACGUCAUUACGUGACAAGACUUUAAAACUAUUAUGGGAAGGCAA

>P1072

CACAACUAAAUCAAGGCUGCGUAAGACCGGUACGCCAACAUCUUCGCAACA

>P1073

UGAAGGCAGCUAUUAGAGACGUCAGACAAUACAUCAGCGACAAGAACUACA

>P1074

GAAUAGGAUGUACACCCUUUAUCGGACUCUUCAUUAUAACGAAUAAUUUGA

>P1075

AAGUUGGGUUCUAUGGAUUUCAAAGACCCGAUAAUAACCUCUACUAUCUAC

>P1076

UCCGCUCAGAAAUUGAAAAGAAAAGACAUUUUGAAUGCUGUCAAAGAUAAA

>P1077

CGUUAAGGAACCCAUAAAGCUAAAGACAUUUGUUGAAAACGAAUGUAAAGA

>P1078

AAGCUUUAUGUAAUUAAGGAAACGGACAUCGAGGAAUUUGCAUCUUUUUUA

>P1079

GGCAGAGGUGUUGGUAAGAAAAAGGACCACAUGUACUUACAAUUGAGCCAU

>P1080

GAGCAAACUGCUGCUUCCGUUCAGGACAACGGAACUGCAAAUAACGUUCAA

>P1081

UAACGAUGAUUUUUGAUGAGUACAGACGUUUCGCAUUUCCGAAGGCCAGGG

>P1082

AUGCUUCACAACCCUGAUGAAUCGGACUGGGAUAAAUCAACAUUUGGACUG

>P1083

GGUCACGAUUUGUACUGGCGCAAGGACACUAUUAACAAGAUCAUAAACGGU

>P1084

UGUGAACUUCUGUAAAGAGAUACGGACCAUAACAAUAGGCAUGAUGAUGGC

>P1085

AUUUUUAUCUUCAUUAAUGUUAAAGACUUUCCCUAAGAGAAAGCAAGUAUU

>P1086

AUGGCUUUGAUACAGAUAAUAAAAGACCAAGGCUGGCCGUUUCCCAAAAAC

>P1087

ACACUAAUAUUAAUACAAAAUCCGGACAAUCUGUUGAGUGAGAAGAUUUUA

>P1088

ACGAAAUUGCAGCACAAAUUUGUAGACAGGCUGGUUUGGUGAGACCUACCA

>P1089

CGAUAAUAGUGACGACAAAGCUUAGACUAUUUAGCAUAUAUAUUAUCAUGU

>P1090

AGGCCCUAUAUGAAAUUAGCUCGAGACUUUUAAAUUCAGGAUACUAUAAAG

>P1091

GUUGGAUUUCGCUACAAGAGAAUGGACAUAUCCUGCAUUGAUGAUGUUUUC

>P1092

CGAAUCAUCACUAAACUUUUCAGAGACGGUGUCAAGCCCGAGCAAAUUGGU

>P1093

GAUAACCUGGACUAUUUAAAGAUAGACAAAACGCUCUCCAAAAGAACAAUC

>P1094

AAUAGAUUAUUCAUACCGCUCUUGGACAACAGUUUUGACAUUCUUCUACCG

>P1095

GUAUACAUAUGACUCUUACGAAAAGACAGCGAGAAGGGACACUGGAAAAAU

>P1096

GCAAACGAAAACUCCCAACUUUUGGACUUUAUAAGAGAAUUGGGAGAUGUA

>P1097

AAGAAAGCUUUGACAUCCAAAAUGGACCGUAUUGAAGUAACAUACAAGCAA

>P1098

CCCUUCUUUGAUAUCCGAGGGACAGACGUUUUAUGAGUUAUUCAAGGGAGG

>P1099

GCUCAACUUUCCAAGGACGAAAUGGACGAUUUAAGAUGGAAUUAUGAGAAU

>P1100

UGGAGAAUUACUUGAACAAUAAGAGACACGAAUUGCAAGCAUUACAAGCAG

>P1101

UUAUUGAAAAAAUGGAAGUAUACGGACUUUGCGUGGUCAAGAAUUUUAUAG

>P1102

CAAGUAAAUACAAGACAUAUCGAGGACACUGAAAGCACGGCCCAUAUAAGA

>P1103

GCGUGUCGAAGAAAAUGGGACCUGGACAUUAUUCUCUCCAACAUCAGCUCC

>P1104

GAUGGAACAAUGCCAAAAACUUUGGACAAGGCUAAAAAUUUCUCAAGAAUA

>P1105

UUGAUUCGAACUCAGCAGACCAUGGACGUUAUUCUCGAAGAGUUGGGACUA

>P1106

CCAGAUGACUACAUUACCACCAAGGACUUGUUGCACAAACCUGAAUACUAC

>P1107

CAUGGUAAAUUUCAAGAUGUCCUGGACAAUUUCUUACAAGGCUGAGCGCAU

>P1108

GGGGCGGAGUACGUUCCAGAUUUAGACUUUACAAAGCUAGUGAAUGAGUGG

>P1109

GAUUACCAAAUUCACUAUCAGUGAGACGGAUAACGUGGCUCUACCCCAUAG

>P1110

UCAGAACCAAAACAGUUUUUAAAAGACAUUGAACUGAUCUACAGGGAUGCA

>P1111

GAGUGGAUGCAAUCUGGGAAGAUAGACGCUUUGAAAAAAUUGCUGAAAACA

>P1112

UACGAGGAAGGCCCAUGUAUCCAAGACCUGCUGAAGAUGCUUACAAUGCCA

>P1113

AAAAACUUUGUACAGCCCACGAAGGACAUUCAAAGGAUCGCCUCGUACGCU

>P1114

GACGACUACGCCAAUGGGUUAUUAGACACUAAAAUCGAAGAACUAUUAGGA

>P1115

UUCUCCCUUGACCAAUUGCGCAAAGACUAUAACCAAUUCAUUGAAAAUAGG

>P1116

GACUUUGGUUAUAUCUUGGGUCAGGACCCCAAACCUUUUCCGCCAUUAAUG

>P1117

CUUAUGUAUGAUGGGACGUUACGAGACGCUAAUAAAAUUCUACCCACGAUA

>P1118

GUCGCUCCUAUUCGACAAGGACCAGACAAAAAUGAAAAAGUAUGCCAAAGG

>P1119

UAAAAUAGAUGAUUGUAGUUUCAAGACUGGUGAAACCAUAUCCAUUGUAAU

>P1120

GACCUUUUCGAUUUGUCUGAUGAGGACGAUAACGACGACAAAGAGAUGUCA

>P1121

AUGAUGAGCCCGCCAAUGGUGAAGGACUUUGGCACGUAUUUGGACAGUGAC

>P1122

AUUGUCCAGUGGCAAGAAUUGAGGGACAAUUAUUUACCAAGAUCCAAGUGG

>P1123

CCUAACGCAGACUCCGAAAAUAAAGACAUUACCGUACAAGGUCCUCAAAAG

>P1124

CUAAAUUCAAGAGGUAUGAAAAUAGACAGUUUCAUCAUGAAGUAUGCCCGC

>P1125

UUAGUAUCACCAGGUAAAUCUAAGGACACUCAUGCGCUUUUCAAGUAUCCA

>P1126

GGUAAAUAUGCUAAAGAAAAAGAAGACAUGUUAUCCCUGCGUCAAGUCUUA

>P1127

AUGGCUUUUGAACCCGAAAUUAAGGACAAUGGCAAGUUGUCCAUGUAUAAA

>P1128

CCCGGUAGCCCUUCAGAUAAAGCAGACAUAAAGGUUGAUGAUAAGCUGAUU

>P1129

CUGGAACAGUUGGCAAGCACAAGAGACGUUGUUCUUACUGAUUUACCGGAC

>P1130

CAACGGCAGAAAUUAAGAAGAUGAGACAAUCUUUGCUGCAUAAAAGAGAAA

>P1131

AUAUGCGGAUGCCGAUCCAAAGAAGACUUUGAUGAAUACAUUGAUACUGAG

>P1132

UUUUUUAUCAUAAGACUCUUGCAGGACAAUUUGAUCAAGCCCAACUAUUAC

>P1133

UUGGAAGUCGGUGUCGCUACAAAGGACAAAUUCUUUACCUUGAGUGCUGAG

>P1134

ACGGUUGAAAAUAUGACCAUUAAGGACGUUUCAGAAAUCAAACUAGCUAAA

>P1135

AGAGUGUGUAUCUAAUUUUGAAAGGACUCAUAAUUGCAGGAUCACAUCUGA

>P1136

CAAUGUUACUUUGGAAAGCUACAAGACGCAGGCCAAGAAACUUUACCACCA

>P1137

AACGGUUCCGCGUCCAGAUCUGCAGACGGGAAGUACCACAUAAUAGAUCAC

>P1138

AUGUAUUCGGAUGAUACACUAAAGGACAUUUCAACAAGCUUUUGUUUUAUA

>P1139

CGCAACAGUAACAACAACCACAAGGACAAAAAGUUGUUACGAAGACUUUUG

>P1140

UUCCAACAUUUUGCCAUCUUAACGGACUGGGAAACACCAUACCUAACAAUG

>P1141

AACGAAUUUCCAGACUCUGAGAGGGACAAAUGGAGUGUAAGUAUAAACGUA

>P1142

AGCGAGCACUACCAAGACAUGUUGGACUUAUACGAUGUCGUGGUAGGUUUA

>P1143

UAUAAAGUAAUUUCUUCGAUAUUGGACAAUUAUAACGACACAACGUUUGUG

>P1144

AUGGUACUUUCAACAGUGAGGAUGGACAUUAUGAGUUAGAUAUAGAAGGCC

>P1145

AAGGAAGUCUACGAAUCUAACAAGGACAAGUACGGUCAAAUCAAAUAUAAC

>P1146

UUCCCGAAACUAAAGAUUAAAAGGGACGUCAAAGACAUUGAUGAUUUCAAG

>P1147

ACAAAACUGCGUUUCUGUUAUAUGGACUAGCCCUGCGACAUGGCUGCGGAG

>P1148

UAUACACCACAGUUGAUUAGACAGGACGUUAUUCCUCUGAAAAAAAUUCCU

>P1149

AAACACUCCAUUUAGGUCAUUAAAGACAUUUGACUUAUUCGCUUUUCAUUC

>P1150

AACAAUUCCUUCUGCUACAAAAAGGACAUUUUCCAUUGUACUGAUAUCUUA

>P1151

GGGCCUUAGCAUUGCUUAGAGUUAGACAACCACCAAAGAUAAUAUUCCUCU

>P1152

UACCGCUCAAUAAAACGUUCAAAGGACUCUAUGCUGAGGCUAAUUCCAUAU

>P1153

AUGGUGGCAGGUGGUAGCGGUCUAGACGAGUUCAUAAAUUUUGACCCAGAA

>P1154

GCAAAACUUAAAGUAACAAGAGAAGACUUUUUAAAUGCACUCAACGAUGUU

>P1155

AAGGUGCCAAACGUGGGGAAGGUGGACGUCGAGUUUGUUGAUGUAACCUCU

>P1156

GAAUAUGAGGAAAUUCACAGGUUGGACAAUGAAAAAGAAGUGCAAUAUAAG

>P1157

UUUGCAACUGCCACUGCAUUGAUGGACACCAUAUCAAACCAACACGUUAAG

>P1158

CUAUAAUGACGUUUUGGGUUAUAAGACAUUGGAUAAUGAUGGCAACGAUAU

>P1159

GUACAACAAAAUGAUACCAUAUUGGACAUUAAUAACAAUUUGAGUAUAAUU

>P1160

GUUGAGUAUAGCCGCUAAAAAAUGGACUAACAGAGCUGGAAUUUAUAAUGA

>P1161

AGAUUCAAUUAUCGGAAAAAGAAAGACAAUUAAUGCUUAAUAAAGUUAAUA

>P1162

CCUUUUUAUGACGAAGAAGAAGAAGACACGGAUUUUAGGCCUUUUACCUCG

>P1163

AAACUCAAGGUGUUUACGACAAAAGACAAACUUUAUUUAACAAACGUGCUG

>P1164

AAAUACAAUUGAAGAAUCGUAUAAGACAUUUUUCCCAGAUGAGGAUACGGC

>P1165

AAGGAAAUUGAAAACGCCACCAAGGACAUUUUCCCACUACAAAACAUCCAU

>P1166

AUUAAAGAAGAAUGUCAAAUUGAAGACGAUGGUAUCAUCGAAAGCCAUUUA

>P1167

AAAUAAAUCCUCCUUUACCAAAUGGACUUCCGAACCAGAGCAUAUCUUUGA

>P1168

AAGAUCGAAAUUGCUAGAGCCUUGGACGAUUUCGGUGUGGACUACAUCGAG

>P1169

ACAGAAUCCACUCGGGCCAGAGAGGACUUUCCUGAUUAUGACGCAAUACUG

>P1170

CACUCAAGGACUUCAUAUACAAGGGACCGUCAAUGUCCACUACUGACUUAA

>P1171

GGAUUUGGAAACGUUAGAAGCAGAGACGGUCAAGAUUGACAUUUAAAUAAA

>P1172

CGUGCACGAUGGUGAUUUUGACGGGACAGACACUAUCAACAACAAAAAGAA

>P1173

GCGUGCGCUUGAAUCUCUGGAAAAGACAUUAGACGAGGUAUACGAACAAAU

>P1174

AAUGAAACACAUAGUGGGUCCAUGGACUUUUCGCCAUUGGGAAAUAACUCC

>P1175

CAGCUGCAGCUAUUCCAUCGGCAAGACUUUAUACAAUUCUACAAAAAUUAU

>P1176

GUCAUUCGGGGAGUUUGUAUGGGGGACACUAUACUUCAUACGUUUAUAAGG

>P1177

AAUGCAGGUGCUAUUAUCCAGGAAGACGACUUUGGACCUAAGCCAAAAUUU

>P1178

UCAAGAUAUUUACUAUCUGGCGCAGACAUUAAAGUUAUGGAGAAAAAUUGC

>P1179

GUCCAUUCCUUGAAAACAAACACAGACGAUUCUUCAUCAGACUUAUGAGCG

>P1180

CCUGGCGAAGAAGAAAAAUAUAUGGACUAUAUCCAGCCAUAUCAUCCGAUG

>P1181

CAAUGCAGUCCUAGUACCCAUAAAGACUUUACUCUCAAACUCAAAAUCGAA

>P1182

GGUUUCGAUUUAGUUCACAUCAAGGACUCCUUGGACAACACUUUCGUCACU

>P1183

AAUUGCGCGCGAUUGUAUAACUGGGACGUUCAGAGAAGGCAAGACUUGAAG

>P1184

ACUUGUGGAAGCUGUCAAAAGCUGGACUAAUGCUUUUGAAAGACAAAAAGC

>P1185

GAAUGAUGUCGAGGCCAAGAAGCAGACUUUUAUUGAUAAAUUGCCGCAAGU

>P1186

AUUUUGAUGACUUUCAAUUUUUCGGACUUUCUAAAAGGCCAAUUAUUACUG

>P1187

GAUAGCACUAAUAGUGAACUUGUGGACAAUUAUGCUAUUAUAGCAAGAAAA

>P1188

ACUGUAUACGCUAGACGAUGAAUGGACUAAUAGUGUGAAGAAACUGGAGUA

>P1189

GGCCAUUUUACGAAUGGCUCAAGAGACAACAAUAACGACAAUAACCAUAGC

>P1190

CAGACCAGUUACUUUAAGAGCACAGACGGGCAUGUCAACCAAUGGGACUUU

>P1191

AGGGAGGAGGAAGACGCUGAAGAGGACUCUCAACCGACAGAAGAACCUGUA

>P1192

CAAUUGGUUGAUGUAGACUUAGAGGACUCUUUGGUUACUAAAAUAAACCAA

>P1193

ACCAUGGGAAAGCAAAUUUUGACGGACUCGCUAGCACCUAAAAUUCGUGAU

>P1194

UCCAAUUUUAAGACAUUCAAUCUGGACUUUUUAAAGCCGGAUUUAGACGAA

>P1195

CUGGAUGGUUCCUCAAAGUUGGAGGACCGUCGAGAUUUGGUCCAUGAUUGG

>P1196

GGGAAAUGGUAUGGAAAACAGGAGGACUUUUGACGAUAUUGAUUUUAUGGG

>P1197

GUUCACACAGAUUCGUAUAUACAAGACUUAAAUGACGAUCAUAUUUUACUA

>P1198

GAAUGUAGUGGAGGCUUGAAUUUGGACAACCUCAAGGAAUAUUUGUGUGAU

>P1199

CAAAGAUGGAAGAAGCAUUAAAGGGACUUUUGCUAAAUCGCAGUUUCUUUA

>P1200

CGGAAGGAAUUUCAGAUGAGGAUGGACAUUAUGAUUCCGUGGCUGUGCAGA

>P1201

GAGAACGACUGUGUCAUUGACGAAGACAUAUUCGAAGAUUCGUCUGACGAA

>P1202

GACUAAGUAAACAUAUAGCCCUCAGACCGGGCGUUGUUCCAAGCGAUUACC

>P1203

AAGCUGAAAGCUCACGUGGGAACAGACGGGGAACUAAGUGAACAAUUUAAC

>P1204

CAUUACACGUCACUGGUAAAUAAAGACCUUGAACACAAUGUGAAUAUUGGG

>P1205

UAUACUGAUCCUAUUUUGAGGAAAGACUGCAUGAAUGAGGUACACGAAAAA

>P1206

UUACUGUAGAUGAAGUGGUCUCCAGACAUUUAAACAGAUUAAAGUUAGCUG

>P1207

AGAGCAAAAGCUGAAAACUCCAAAGACGUUGUAGUAACAAACUUUUUUAUA

>P1208

AAAUAUUGCGCAAUUCUUAACAAGGACCGUUCAGGUUAAUAUGGGUGAAUU

>P1209

GUUAUCAGUUGUAAAGGUGGUGCAGACAAUGCUACCUUAACCCCCAGAGGC

>P1210

UAAGUCGUUCAGAUAUUUUGAUAGGACAUUCGCUACAGAAUGAUUUGAAAG

>P1211

AGUUAGUGAGGUCGAUGGUAUAAAGACAAUUAAAAAAUGUGCACGGUGGAU

>P1212

GAGAAAAGCGUAUUUGAAGAUAUAGACAUUAAAAGAAAGGAAAUUAUUAAU

>P1213

GAUUUCUUACUUGAUCACCUCAAGGACAUUAAAGCUGAUGAUUCCUGUUUU

>P1214

UGAUGACAGCGAUCUACUAUAAAAGACAAUAUGCGUAAUGAAAUUUCACUU

>P1215

GGAUUUACCAGAGAGCAUUUGGGGGACAAUCCAAAGAUUUUGGUAAAGGUG

>P1216

UUCCGAUUUUUCUGUGAACGUAAAGACAUUGAACCCUUCGUUCUCUGAGAA

>P1217

AGCGUUGGUGCUAUGCCUCUAACAGACAAUUCCCCCGGAUUCAUGAUGGAU

>P1218

UUGUGGUUGACAGUAUACCCAUUGGACGUUGUGAAAUCCAUUAUACAAAAC

>P1219

AAUCAAGGCGUAUUUUGAAGGGCAGACGAUUGAAUUUAAAUGUGCUAAUUG

>P1220

AUAUCAAACGGAAGUGAAAGCAAAGACUCUGACAAACCCUCUUUACCCUCU

>P1221

ACUCCUCAAACAGAAUUCCACACGGACGCCAUUGCUGACUUGAAUGUUAAG

>P1222

GUCUUCUAUUGAUGAUGACUCAAGGACUUACCUGCUAUAUUCCACAGAAAC

>P1223

UCUACCUUGGUUAGUUCUGGUGCGGACGCUUGCAUUAAAAGGUGGAAUGUU

>P1224

CGAAUAGUCAGAAUGAUAUAAUAAGACAUUUAAAAGAAAUGAGUUUUGGUU

>P1225

CACCAUGGACUGUCAACAAUACGGGACAUUUUCCACUUCGGGCUCUUCUAC

>P1226

CCUAUUGCAAAAAUGUUCUGGCAGGACGAAGGAUUGAAUAGCAGCGUUUAC

>P1227

AUAACUAAUGGCAUGUUUAUCAUAGACGACAUCGAGCGUAGUAAAUAUAAU

>P1228

GCUAGUUGCGAGAUGGCUAAAAAAGACAGAGAAAGAGACAGAAGCCAUGAA

>P1229

UCAGCUACCUGCUUUUGCACAAAAGACUACAUUUAUUGCUACAUGAACUCU

>P1230

UUUGAACCUGAUGACCAGCCCUUAGACAUUGAAUUGAAGUUUGCCAUCUUG

>P1231

AGUUUUUGAGGGUGAAAGGACAAGGACAAAAGACAACAAUCUACUGGGUAA

>P1232

UUCGCAAAAGUUUGAUUUAAGACGGACUCCAAGACAAAUUGGUUAUAUCCU

>P1233

GCCUCUUUGGCCAUUUGCUACCCAGACAUUUGGAAAUUAUAUAUGAUAUCA

>P1234

CAAAACGAUUUUAAAGAAUGAAUAGACGAAUUUCGACGAACGAUGAAUGUU

>P1235

AUGAUUGGAUGUGUAGGUGAUACGGACACUAUUCAUCUUUUCAAACUUGAU

>P1236

UAUAACACCUUUACUUCAACCUUGGACCUACCAAUCAAUGAUCAAUGAGUA

>P1237

AUCCCAGAGAUUCAUCAAAUCACAGACUUUAAAAAAAUCGAGAACGAGUAA

>P1238

GAUGCAUUAGGGCAAUCAUCUACAGACUUUGCAACUAACUUUUUGAAUCUA

>P1239

AAUGUACGCUUUUGCAGUGAUACGGACUUUAAUGAUGUGGUGGAUAGUGGA

>P1240

ACAAGAAAAGAUUGCGGAUGAAAAGACUCUAGUUCAAUUACAAGUGGAACA

>P1241

CAAAAUGAAUACCCAAACAAGGUGGACCCUUUUAUAUUGGCUAUCAAUUUG

>P1242

AUUACCUCUUAAAAGGUUUCAGAAGACGUUUACUAUUUAAUUCUUAGCUAC

>P1243

UGAAGUUUGUCCGUCCCGGUUACAGACUAUUAAACUCUGUAGACCGUCGAU

>P1244

UAAGACAGCACAUGUUGGCAAAGAGACACUGUAAAAUUCCCUACGAAAGCG

>P1245

UUCGAACAGGCACUGCGUGCGAUGGACUAUGUCUUAGAUGAUAAUGCUGAC

>P1246

UAGAAACUUGAACAAAAUCACGAAGACUUUGAACUAUUUGAGAGCCAGAGA

>P1247

UUUGUUAACGUACAUAAGGUGAAGGACUAUUAUACGGUCACCGAUAUCAAA

>P1248

ACCAAAUCAGUACCAGUAAAAUUGGACGAAUUUUAUUCCUCCCAUAAAUGU

>P1249

UGGGUCAAGAUAUAGACAAUCAAGGACGUUAAAAACCAAAUUUCGUCGACU

>P1250

GUCACCAACCCAUCUGCUUACACAGACGCUGUUUCUGUUUGUCCAAAUCUA

>P1251

CUCUAGUCAACAAAAAAUUGAAAAGACUUUAAAGAUUGCUUACACAGAGUA

>P1252

UAUGCCAUAAAAAAUUUACCACAGGACGUUGGUUUUGCUGAAAAAAGUUUA

>P1253

GUGAAAAGACUGGGCACAUAUAUAGACCUUUUACAAAUUCACAGAUUAGAU

>P1254

GAUAACCUGUUAGCCGCUGUAAAGGACGCUACCUACACGGGUAUUAAAGAA

>P1255

GCGGGAUGCAGUCUUGAUGAAUGGGACAGUGGUAUACGAUUCAAACGGCGC

>P1256

CCCUCGGCCACCCCAAAUAUGGAGGACACUUUACUGACUUUUAGUAUGGGU

>P1257

UUAAACGAUCAAGUAUCUAUUAAGGACAAUGAACUACAUAUUCUCGAUGAG

>P1258

CUAUUUACAUACUUUCAUACAAUGGACAGCAGAGAAUCCCAAAAAUUUGCC

>P1259

GGAGGGAAAUAUAGAAGACGAAUGGACUUUACCUCAAGAAAAUUCUAUGCU

>P1260

CGAAGAAUACAUACAUCAAUCGAGGACAUUUUUUACAUUUUAUUUCAUAAU

>P1261

UUGGGUGACGACGUUGAAGUUAAGGACGAAAUCUACAUCAACGGUGGUAAA

>P1262

ACCAAAAAAGGGUACCAUUACAUGGACGAGAAAUGGGACUUGGUCGUUAAU

>P1263

AAAUGCCUACUUGUACUAUCUAAAGACAAAAUUACACACGUAAUAAGAAGG

>P1264

GCGGCAAAUUUGCAUGGCAGAGUGGACAUUGCUGAAGUGUUUGACACGUAU

>P1265

GGAGCAGCAAAUGUGGAUGACGGGGACGACAAUGAAAGCAAUUACUACUGC

>P1266

GAAGGGGAGGAAUCAUUCGAGUCGGACCAAGCAGAACGUAACUCCAUCCAU

>P1267

GUUGAAACUAGUAAUAUUUUACCAGACGUUAAGAAAGGGUUUUCUUUGCAU

>P1268

GUUUGAAGUGCUGUAUUUCUUAUGGACAUAUUCGUACAUAUUCCGAGAACG

>P1269

AACUUUGGUUUCGAAUCUGAUGAAGACGUUAACUUUGAAUUUGGUAACGCU

>P1270

GUAUCAUACACGUCGGAAACAUUAGACAUUGUAUCCGACUAUGUACAAUCU

>P1271

CCCGUUCUUUCGGAAUCCAUGUGGGACACUAAGAAAGAAGUUAAGGAGGCG

>P1272

AGUUCCUGAUCAGAAACUUUUCAAGACCAGAUUGGACUAUUUAUCCGGUAA

>P1273

GCUAUGCAGUUAGGUAUGCCAUUAGACUUUAACGCUAAGGCACAGGAUACA

>P1274

UGCAGGAUCGAAGCAGAUUUUAAAGACAGAAGCAGCCAACGUACUCGAUCC

>P1275

UUAGAGCUACUGAAUCAAAAGAUAGACCCUACUAAUAAACAUCAUUUUUUA

>P1276

GGAGGAAACAUCUUCUCACGCAAGGACGGUUUCUAUUGACGAUAUUCUGAA

>P1277

AAAUUUUGGUUAAAUGGUCUAGAAGACGACGAAACGACGAUGAUGAACAUU

>P1278

GAGAAAUUGUCAGACAGUACGGUGGACACCACAAGGGAGCUGUAUGUGUCG

>P1279

UCUCAAAUUAGAAUCUGGGAAUUGGACAAUAUAGAGGCUCCUGAGGAUGUC

>P1280

GGAAAUUUAUUUCUUUGGUUAUGGGACUAUUACCAGAAGUUGCACCCAAAA

>P1281

AUGGUCAAGCUUCGUGUUUUCAAAGACGAUCUAAUAUUCAAAUCACAACGA

>P1282

UCCUUCUUGGAAUCCGUCAUCAGAGACUCUGUUACUUACACCGAACACGCC

>P1283

UCCGGGCUGUUGCGUUUCAACGUGGACGCCAAGUUUCCCCUAGAGGGCAAG

>P1284

CGUAAGUGUAUACUAAACUUUAAGGACAGCUGGUAGGACAAUUUUAUGUUU

>P1285

AAGAUGCCACCAAGAAACAAAGAAGACCUAUUCCAAAGGGUCAUUGUACCG

>P1286

CAAAGAUAAUUUGCUUGGAUUUGAGACGGAUUUCAUGAAAAAUCUCGGCGC

>P1287

UUCAUAAAUCUUUCCUACAUAAAGGACAACAAUAAUCAUGCUACUAGUAAU

>P1288

AUUAAAUCGUUAGAAAUCAAUAAGGACAUAGAAAGUGGCAAGAGAAAAUUG

>P1289

GGUGAUUAUGAACAGCUAGAUAUGGACAAACUAAUGGAACUGCCAAAUAAU

>P1290

GGUUUAGAUUCUCAACUUCUUAAGGACGAUUCUGAAGAAAUCCUAGAAAUA

>P1291

AAAUUUGAAUUUGACAGUUUUAUGGACUCUAUAGUGCAGUCCCAACAGACU

>P1292

UUAACAAAUAGUUUGGUUUCUAUGGACUUAAACGCGAGGUGCAAGGGGCCC

>P1293

UCAAAAACUCAAAUGAAGUAUAAGGACAUGAGAAUCAAGACUAUUACAGAG

>P1294

ACAAGAAAUCAACUCCCAAUUAUGGACAUUAUUAUUGCAAUAUUUAAAAAU

>P1295

GAUGAUUCCAUACUUCCUGAAAUGGACCACUCAAAGGGACAAAAAUGACAA

>P1296

ACAAGGUAGUAUCAGCUAUAACAGGACUAUUCUUACCGGCUUAUGGAAAUU

>P1297

AUAUCGAGUAUAUGAAAUUAACGGGACACUGUGUGAAAAAUUUGUAGUUGU

>P1298

AGAAAAGAUUGGCCUUUCCCUGGAGACCCUAGUGGAAGCCGUGUUGUCAGA

>P1299

UAUGCCGCAGCAAGUGAAGAUAUGGACACACUCUGUUAUAGAACACCCUUC

>P1300

AGUAAUCAGAUUGCGGGAGAAAUAGACAAUAAUUCGUUUCCUCCUCACUUU

>P1301

CGGAUCACUUGGAGCUAUUGCAUGGACAUUUUCGCAGAUAUUGUAGAAUAA

>P1302

UAACAUUAAUGACGUCCAUGUAAGGACCCCUAAUUUUGAAUCGAGAUUGAA

>P1303

ACAAGAACAAUAACAACUUUUCUAGACAAGAACAUCAAGGAAAUGGACUAU

>P1304

ACAAAGAACAAGAACUUACUGAUAGACUAUUAAAAGCUAAGCAAUUUGUAU

>P1305

CAACCAAUUUCAGUCGUUGAUGAAGACACUUAUUAUCAAUGGCAGGGUAAA

>P1306

UAUUAACAUCCACGACGAAGAGAAGACAUUUCAAAAACUAACGUUCAAUUC

>P1307

GCAACCCACAGCACCUAUGAAAAAGACUUUGCUAAAACAGUUAAUUGAGCA

**II. List of 1,307 RNA samples in the negative subset**

>N1

GAGUUGAAGUAAAAAUAAAGAAAGGACCAAAUGAGAAUGGGUAUGCUUGGU

>N2

AGAAAAAAAAAAAAUGUACACCCAGACAUCGGGCUUCCACAAUUUCGGCUC

>N3

AUUCUCUCUUGUUUCUAUUUACAAGACACCAAUCAAAACAAAUAAAACAUC

>N4

CAUCAUUGGUACCAUCGGUCCAAAGACCAACAACCCAGAAACCUUGGUUGC

>N5

GGUAGACCAUUGGCCAUUGCUUUGGACACCAAGGGUCCAGAAAUCAGAACU

>N6

GAUUUGCCAGCUUUGUCUGAAAAGGACAAGGAAGAUUUGAGAUUCGGUGUC

>N7

GAAGUCUUGGGUGAACAAGGUAAGGACGUCAAGAUCAUUGUCAAGAUUGAA

>N8

UGGAAUCCAUGACUUACAACCCAAGACCAACCAGAGCUGAAGUUUCCGAUG

>N9

CCCCAAGAUUGGUUUCCAAGUACAGACCAAACUGUCCAAUCAUCUUGGUUA

>N10

CGAAAAGGAACCUGUCUCUGACUGGACUGAUGAUGUUGAAGCCCGUAUCAA

>N11

UAUAUUUUUUAAAUUUUAUAUAAAGACAUGGUUUUUCUUUUCAACUCAAAU

>N12

CCAGUGAACUAUGUUCAGCUUCUGGACUUCGUUUGGUGCUUUAAUCUAUUU

>N13

AACUGGAUACCAAUGUUUAUUUUGGACCCUGUGAGAUAUUGACACAACCUA

>N14

CAAUACUACUACAGAAAUUCGUUGGACAGUACUUGCAGAUGGGCAAAAAGA

>N15

CCAGGAAAUUCUAAAACGUAGGCAGACGAGCUCAGUCAAGAGGAGAUGUGU

>N16

UCGUCCAAGUUCCAAUCUUGACCAGACGAAAUAGGCACGGCAUUAGGAAUU

>N17

AGAUAUCUGGCGGAGUUUUAUUCAGACGAAUCCCCACACCUUCCAGUUCCU

>N18

UCAGACUCAGUACCAGUCAACUUGGACAGUAAUGUAGAUUUCCCCACCGAC

>N19

GCUUUGAUUUUUUCAACUGUAGUAGACAUUUUGCUCAAUCAACAACUCUAC

>N20

UACUGGGAUGAGGAAUUCGAAGAAGACGCCGCCCAGGGCGAAGAAAUCAGU

>N21

UACCUGCCGGUUUGGCUGCUUUGAGACGUCAAUUAGAAUUGAAGAAACAAC

>N22

AAGAAAAGAAAUUAUUGGAAAGAAGACGUGCCGCUUUAUUGUCUUCCGGUA

>N23

GCCAUUAAAUCUGACUCUAAGAAAGACUCGGAAGUUGUACCUGAUGACGAA

>N24

UCCACCGCAAGCCAUGAAAAUGAAGACCAAAAUCAAGGCGAAGAAGAAGAA

>N25

AGACUAAAUUGUUAGACAAAAUCAGACAAACCAACGUUCAAGGUGGUGAAG

>N26

CAUUAUGCAUGGUUUGGAACAACAGACUAUUGAAUCUAUCAAACUGUUAAG

>N27

GCCAUUCCAAACAAUUCAUUCAGAGACUCCUUUGCAAAGCAAUCAAGAGCU

>N28

GAAGCUUUGUUGGAUUUCUUGAAAGACAUGAAAAUCCCUGUGAUGUCUAUC

>N29

GAAGGAAUCAAGAUCUUUAAUGCAGACAUCAUCUAUCAUUUAUUUGAUUCA

>N30

AAACCGACCCUACUACAAAGGAAAGACAAACUUUGAUAUUAGGUAAAGUCA

>N31

ACUUUGAAGGAUAAAGCUUUUAGGGACCAAGUUGCUAGAUCCGAUUGGCUG

>N32

AAGAACACGUUGAAACUGGGUUAAGACGAUCAAAACAACCAUGUCUGCUCC

>N33

GUUUUUAGAUUGGAGAACCAAAAGGACCUGGUCAUUGUAGAUGAGAAUGAG

>N34

CUAAACGAGUUACUGAAAUGCAAAGACGAUGUACAAUUGCAGAAAAUUUUG

>N35

ACAGUCUGCCAAGCCGGAUGCGAGGACGCAAGCAGGGGAUAUAGUUAGUAU

>N36

GUAAUCUGGUAAUUUUUUCCAUGAGACGUUUGGUAUAUAUUGAGGAGCAUC

>N37

GUGAGAACUUCUUGGCGCGAUGUGGACUGGUAGUCAUCGUUCUUCUUUGUG

>N38

AAAUUGCUGAAAAUUUUGUAUUGAGACAGAAACCCGUAGGUGGCGUAGCGG

>N39

CCAAUGUAAUUCAUAUUUAAUUGAGACUUUUCAGAUUCAGGAGAAUAUAAU

>N40

UUAGGAAGAAGGGGCUUCUUGAUAGACCCCUGCACCUUAUCUAGCGGAGGU

>N41

UGCACCUGUGGGUGAAGGAAGAAAGACGAUAUUUGUGAGGGAACUGAAUUG

>N42

GUUGAGGAAUCUGUGUCGAAAGCAGACAUCUUGCUUUCGCAUCACAGAGCA

>N43

CGUGAACCCCGUCAAGGUAAGUGGGACCAUCAAGAAAAUAGAGCAGUUUGC

>N44

AGGGAAAACGAAAACGAAAACGAGGACGAUUAGAAUAUAUUAAUAUAUAGA

>N45

GUAUAUGCUUUCAAUAUCUUGUUGGACGAACAGUGGGUUGUCAUAAACCUG

>N46

GCAAGCCGCUUCAAUUCCAUUUUGGACCUGAUCUCUUGUCUUGCCUUCUUG

>N47

GGCAACAAUGGAUCCAUUUGCUUGGACACUACCUCAAUAUGGUGGCUGUUG

>N48

AAAGGAACCGAUAGUUCGAAAUUAGACUGUCUCUUUGGAAUAAAAUCAUCG

>N49

AUGUAUUCUUUUUUUUUUUCCAGGGACAUAAAGAGUUGUUUUUAUAAGGUG

>N50

UCUUGUCUCAGCGUGUUAUAUUUGGACUUAUGCUGUAGCGGCUUGAUCAUU

>N51

CCAGCAAAUCUCACCCUUUUGAUGGACAAUUCACUGGAGUUUGACUCCGGA

>N52

AUGGUUGAGGACUGCAAAGAAUUGGACCUUUGAUUGUUGAGCUUUGAAGGU

>N53

GUCCUUACAGGCGAUACUGAGGGAGACCUAGAAGAAUUUGAGGCUAAAUGC

>N54

CAAUUGGAUAAUAAUCCCAUAAUGGACCUCAAUUUCGGUAUAAUCUUGCAG

>N55

AGAGAAACUGAUAAUAACACACAAGACAGAAAAAACCCGCAAGUGGAAAGU

>N56

UGCAAUUUGUAAAAGGAAUGGGUGGACUUAUCCCUUACUGGAUCAUUAAUC

>N57

UUUAUAGCAUUUUGAGCAAUGCAAGACAGCGAAUUUAAAAUCUUCCAUUGU

>N58

UUAACGAGGGGAAUAAUUUUGUAGGACUCGUCAUUGUCUCUUUUCUUCUUC

>N59

UAAACAUUGAAAAUUUGCGCCAAAGACAUAGCAAGCGCAACGUAUUCAUUG

>N60

CCAGAAUGUACAGUCUAAGUCAAGGACUGCGGAACUACAAGCUGUAAGUAC

>N61

AUAAUUUCCAUCUUCCCUGGCAAAGACAGGUUUUCUCUUUUGAUCACGUUA

>N62

ACCCAUACUUCCAAUUUAACGCGGGACAUUUGGUUAUUAGGUCAUUAAAUA

>N63

GUCAACAACUUGAAAUUUACACAAGACAGCAUCAAAUUCAUUUAAGAAUGU

>N64

CCACUGUAGUAAUGACGUGUAUAGGACCCAGAACAUUGUCUCCAUUCACCU

>N65

ACCACAAGCUCUUAAUUGCGAUAAGACCAUCAAAUUAUCGAACUCCCAUGG

>N66

UGGUUGUCGCUGAACUCAAUGAAAGACCACUCAAUUUCCUCCUUUACAUAU

>N67

GUCUAAAAUACCAAUAAAGGAAAAGACAUGAUCUUGUUGAUCCAGCUCAGG

>N68

UUCAAAUUAGUGACAAUUUUUUCAGACCUUGUGACGAUUUGUUUCUUGACA

>N69

UCCAAGAGAUAGGUUCUAAUUUUGGACCCCCUGAUGGUAGUAUUCUCAUCA

>N70

AACAGUCUGGUUAGCCUUUUCAUGGACCAUGAACCUGUACGCCUCCUCUGC

>N71

AUCAAAGGGAUUAGCGGCAAUAAGGACAAUACCAGAGUAAGUAUAUAUCUG

>N72

GAUAGGGUGGUUAAGUCAUCAGUAGACUCUAAAAUAGGUGGGUUUCGUAGU

>N73

CAAUUGCAGGUUUUCGAUAAAAGAGACCCUAUUCUCAUCUACUACUGCCAA

>N74

AACCCGGAGAGUGAGUUUCUUAAAGACCUAGUUUUAUUUUAAGGGUUUUAA

>N75

AUAGCAAAGAUGGUGUAGAAAAGAGACCCCUGGAAGAUGUAAAGCAAAUGA

>N76

UCACCUUCCCGGUCCUUCGUGAAGGACAAGAAAAGGUUGGUUCACCGGUUU

>N77

AAUCCCAUUGGGCUUUCGCCAAAAGACGCCUGCAAUGCAUCGUUUUCGCAG

>N78

UCAUAUCGGAUUGCAGUUACAGCAGACAGAAGCUUUACUAAAGCACAGUUU

>N79

AGAGCUAAGGGACUUAAGAUCCAGGACCAUUGGAUGGAAAGAGCUUGUUGA

>N80

GAGAAAAGAAUGGCUUCUUGCAAAGACAGGUUGGCCUCUAGGAAGGAAGUA

>N81

GCAGAUGCUUCAAACCAAUGAUAAGACUAGCAAUAAGUCGAAUGCCACUAU

>N82

AAUCUUUGUGACGAGGUCAAGGAGGACCUGUUAUUGAUUGUUUACGAACUA

>N83

CCUAAUUUGUUAAUAGAAAAUGGGGACGCUGAUGCCCUGGAUAUACCGGUG

>N84

UACAUGUCUUGCAUAGGCCUAGAAGACAUAAGGUACGUACCCAAUCGAAGC

>N85

GUAGUGUAGACAGUUACACCCCAAGACCAAUGAUCAGCUAGUUCGCCGUGC

>N86

UCUUUGAUAAGACCAGGAGCCAUGGACAAGUAAUAGUUUUUUUAAAAAUUU

>N87

UCGCCAAUGGCUAGCAGUAGUGAAGACGACUUUCUUUUUACCAUUUUAACA

>N88

GAUAAAUGUUUGGCAUCUGCAGUAGACAUAAUAACAUUACCAAUUGAAGAA

>N89

AGUUGGCAGUUUCAAUGUAGCAAAGACCCUCCGGUUCCGAAGAUGACAAAA

>N90

UGUGGUGUAUCUAUUAGUUGGCGAGACGUGAGGCACCUGUUGAAUGGCAGA

>N91

GUUUGAACGUAAAGGCAUUUUUGAGACCAUUACCAAACCUAGCAAAUAAAC

>N92

AUUGCUCAUAAAUGGAUUUUCAUGGACAUCAUUUUCAAUGUUAUCAUCAUC

>N93

CCUCAUAUUCGAUUUCGACUUUAGGACGUUUAGUUUUGGCACUAGUUCCCU

>N94

CAUUUGACCCAUUAUCUUCUUCCAGACCUUUUCGUCAACAUUUAAUGGUUU

>N95

AUCACUUGCCAAACAAUUUCGUCGGACAUAUCCUCGUUGUAUUCUAACCGU

>N96

UUUACAAGAGGAAUUUGGGAACUGGACGAAAGUGGCACAAUACCUCAUGUG

>N97

UUAACUUUGAAAGUGUAAAACUUGGACCAUUGAACCCUUUGAAUGAAAUUC

>N98

CGAAAGAUAUACGACGAUGAAAGGGACGCAACCCACCAGUGGAUUCACAGA

>N99

UCAGUAAGUCGCCUGGUUCAAUCAGACGCCAUGCAUCAGUGAAUUUUUGAA

>N100

AUCAAGGUCAUUUGUUGCGCUACAGACAGGGAAUCGUACAUUAGUAUAAAU

>N101

UGUUGUUUUUCGCAUGGGUACUUGGACUUUCUAGGUCAUUAUUGGAAUCGC

>N102

UGGUGUGGCACUGGUGAAUCCCUGGACGUUUCGAAAGUUUCAACAGUGCUA

>N103

CUCGUUGAGAUACUGAUAACGGUGGACGAUGUAUCAGAGUACAACGCGCUU

>N104

AUCCAGUUUUUGUUUUUAGAGUUGGACUAUCUGUAUUAUUCACGAUGUUUC

>N105

UCUUCCCUACUAUCGUUUAAGGGAGACGAUUCUUUAAGAUCAGGUAGAACA

>N106

UUUUAUUGUAAUAUGCUGUCGUGAGACUUUUUACUUUCCUUCGUUGCAGUG

>N107

AGCUGGUUUUGUAAAUGCAGUAGGGACUCCACUUCAUCUAUGGUUCUUGCA

>N108

CCAAUAGCGCUGCGAUGUAACACAGACAUUGAAGAUGUAUUAGAUCUACUA

>N109

AAACGCUUUGGUUACGAAAAUCAGGACUGGCAAAACUUUGCCUGGCGUAAA

>N110

AUUCAUUAUAUAGUGGUUUAUAUAGACCACUUCUUCUGCUGGUUGAUAUAG

>N111

UAAUGAUGAACGUAGGUGACUCUAGACAUGAUAACGAAUGGCAUGUAGUGU

>N112

AACGACCGUGGCCAUGAAUGCGAGGACGGCAACACUAGAAGCCCACGUGGA

>N113

CCUCUUGAAUGGGUUUUUCAUGCAGACAACCUCUUGUUGUCUGAAACCCCA

>N114

AGGAACCUAGAGCAAUGUCACGGGGACCUUGUCCGACGUCAGAACCCACUA

>N115

AAAUGCAGAAGGGGACGAUAAUAAGACCAAAUAUUCUUGCCAACCAGUGGU

>N116

CACCACUUUCUAGAGAACGGCUUGGACCUCUGGUUGUGGAACAUAACAAAA

>N117

CAGCAUUUUUCCUAGGGGAGGAUGGACAUCGUGGUAAAAUUCGUGUCUCAA

>N118

AAACAGAAUGACAGUAGCGAUGUGGACUAAGCCGGUUCUCCUUGGUAAUGU

>N119

CCUGAUAAUAAAGUCAAUUAUGAAGACGAAUACGUAGCUUAUGAUGCUACC

>N120

CAGGUACUUUAGUUUGGCAAAUAAGACCUCGAAAGGCACUUUUAGUUGGAG

>N121

AUUUCCUCCUCAUUGGAGCGCAGAGACCCUAACAACACAUCAGUGAUAUGU

>N122

ACCUUGACUGUACUCGGAACCGAAGACGUUGGCUAUGGCCAUGAUACCAUU

>N123

UGUAAAGAAGCACAUGACAGUAAAGACAAUAAUCUCCCACACAAGCCCGUU

>N124

AGCAUUGACGAUAUGGUAGAAAAGGACAUCAUAGAGCUUGUGAAGAUCUUU

>N125

GGAUACAGUUCCACGGCCAUAAAAGACCUAUUCCUAUGGCGAAAAAUGUAA

>N126

GAUACUUAAGUAAGAAAGAAACAGGACGAAAAAGAUACGCAAAAGAGAGAG

>N127

ACCAUUUCCCUCUUGAUGUAUUUGGACACGGCCUGUACUUAUCAAUUCGUA

>N128

CAGCGUUUCCAGAUGAUCUAACAGGACACCUACUUGGAAUGUCUUGACAUC

>N129

ACCCAAUACUCUUCAAAAGUCUUAGACUCCACUUCUUGUAAACACAACAGA

>N130

UUGACAUAAGGUGUUGUAGGAUAAGACGGUGAACGUCCUCUUGGCUAAAUC

>N131

AGGUCCGGGGCCAAAUGGGCCCGGGACCUGCUGCUGUGCCUGUUGCUGCGC

>N132

UUGGGUUGCCCCAGAGAUUGUGCGGACACUGCAGCCAGAUGCAAUUGAAGC

>N133

CACGAUGAGAAUUAAUGCUGAAUGGACAUCUAUGUAUAUGAUAGUGGGUAU

>N134

CAGCAGUAUACAUUGUAACAGUGAGACUUGCCUUCUUGAUUAGCAGUAGUU

>N135

AUGGCCACGUAGUCUACGGCCACAGACCCGGUAUCGUACACCAAUACGGGC

>N136

AGUCCCAGUUGCCUUUGCCCAUUAGACCCAAACGCAUACACACAACUCAUC

>N137

UAUUCGAGUUUGUUGCUACUGGUGGACACCCGACUAUCUACAGUAAGGAAC

>N138

GGAUGACGUAGUGCAAGUGCCCGAGACGUCCUCUCCCACCAAGGUAGCAUC

>N139

AUUGAGGCAGCAAAGACAAAAUUGGACAUGGAACAAGAACGCAUCUCCCAA

>N140

GAGCUGCCAACUAACUUGAAUAUAGACUCGGAGGCACUGAGCAAGUUGCCC

>N141

GAGGAGGAAGAGAGUAUGAUGACGGACGAUGACGAUGCAAGUGGCGACGAC

>N142

AGAACGCAUCGAACAAGAGAAAAAGACGUGCGGCUGCAUCCGCCAAUGAGA

>N143

AACAAUAACAAUACCGCUGGCGCAGACGCCACUAGCAAGGAAAAAGAAGAU

>N144

GAUGACUAUGAAGAGGAGGACGAAGACUAUAACGAUGAGGAAGAAGAUGUG

>N145

AACAAGAUGUCAUGUAUCCUUGCAGACGACAUGGGUCUAGGUAAAACAUGU

>N146

UGAUGAAGCCAUUUAUUUUGAGAAGACGUAAGGAUCAAGUGUUGAAACAUU

>N147

AUGGUUCCACGCAAGUGAAUGAUAGACAACUAUUAAUAGAUAAGUUUUAUG

>N148

CUGCUGAUAGGGCACAUCGUGUGGGACAAACAAAGGAAGUUAAUAUAACCA

>N149

AAAAAGAGAGUUGAUAGAACAAAGGACAAAGAAAUUUAGAACGGUAAAAAU

>N150

GCAAAGCCCGCUAAAAUGGGCACGGACUCAAUCAAACCGGCCAAGAAACCA

>N151

GAGCAGUUCUGUAGCAUCUUUGCAGACACCACCGAGCCCUGAUCAAGAGAA

>N152

GUAUCAUCGCCCUUAACGGAUGAAGACAUAAAGGAGUUAGAGUUUCUUCCG

>N153

GGGCCGUAGGAGCAGUUUACCAAGGACAUCGGCUUCUGCAAACCAUUUAAU

>N154

AUGAAACUUUUUUACCGCACCAUGGACCCUCUAUGGAUCUAUUGAAUGAAC

>N155

GCAAUUUUUACAUGUAGUCAGGAAGACCCUUGGCAAUUCAGAGCUGCGAAU

>N156

ACAAAUGUGAGUUAUUAUCAGAUGGACCCACAUUGUCUUCCUCUUCUACAU

>N157

GUAAAGUUCGCAAAUGAUAUUAAAGACGUCAAGAGUAUAAGCCAAUCGUUA

>N158

UUGCUUUGGGUUACACAUUCGAGAGACGUGGUAUUUGAAGAAUAUAAUACA

>N159

AGGAUAUCCACCGGGACAUUAAAGGACAUGAGUAAUCUGUCAACAUAUGAG

>N160

GACAGUGGCGAGAUGAUGCUUGCAGACCCCGAAAUGAAGCACAAGUUAGAA

>N161

UUAGCGGGAUCACCUAAAGACGAAGACGGAAUCAUAAUGACUAACAAGCGA

>N162

CAAAAGCAUGUUAAGAAGUUUUCGGACUUCGUAAGUCUGCAAAAAAUGGGU

>N163

UUACGGUUACUGGAUUUUUUUGAAGACGACGAUUACUAUUAUAUCGAAACU

>N164

CGGACCAUUUGAUGUUUUUGUUGGGACAAUAGAUUAUGCUGCCCCUGAAGU

>N165

ACAGCAUCCAAAGCAGUUAAUCAAGACUUAAAUAUAAAAAAUUUACAUAUU

>N166

UAUUACCAUUAUUAAAUCACUACAGACGAUAAUACCCGGAAUGCCCUUUUU

>N167

ACAACGUCAAACUCUAAGGACUCAGACUCUAACGAGUCAUUAUAUCCCUUG

>N168

ACUCAAAAUUUGGGAUUAUCUACAGACGAAGAUUGGGAUUACAUUUCUAAC

>N169

CAACCAUUUUUGAACCUUUGUGAGGACAACGAGGGAGAUGUUAGGGAAGCU

>N170

CCUGCUGUGCAGAAUUUGAGUAUGGACGAAAGUGAAACAGUGAGAUCUGCU

>N171

CCUGCCAUAACAGAAUUAGCCAAGGACGUGAAUUGGAGAGUUAGAAUGGCU

>N172

GGUGUCGUGAUGAAAUUAUUUCAAGACUGCUCAAAUUUGAUCUACAAUUAC

>N173

CCCUCAUUGCAAACGCUGUGUCAAGACGAAGAUGUUGAUGUAAAAUACUUC

>N174

UGCUGGAUUUCCUUGUACAAUUGAGACUGCAAAUCCCAAUCUAAGGAGCUU

>N175

CGAUGAUAGCAUCACCCCAAGAAGGACCUGUAAUCUGUAGUCCCUCGGUGA

>N176

GUAUGUUUCUUGAAACUUGUAAGGGACUUUCGUCGAGGCCGGAGUGACAAG

>N177

CCAGUCGAUAUCCACGGGUUGAGGGACAACCUCUUCUUGUUUGAUUUUGGU

>N178

CUAGAAUUGUUAAAGCCAUGCAUGGACCGAGAGGCCCUGUUCAAAUUCCUA

>N179

UCAUGAGUCCUCCCAAUAGACUGAGACAGUGCGCCCAAGUGGGAAUCUUGC

>N180

CGCACUUUUUUCAAAGACUCUUUGGACACCUCAUCAGUAUUGCUUGCCACC

>N181

GAACAGACCACAGCGAAAGAACAGGACCUUGAUCAAGAGAGCGUGUUGAGC

>N182

ACCGACGCCAGGGACGAGCAAGGGGACGAAGGUGAUAAUGAGGAGGAAAAC

>N183

GUGGGCCCAGUGACCGCCACACUGGACCCCAUACCACUUCUUUUUGUUAUU

>N184

GCCAUUCAUGCCGGUGAACAUGUGGACGUUCACGGUUCCGUGAUCGAACCC

>N185

ACAAAGUACAUCAACGGUCACUCAGACGUUGUGCUCGGUGUCCUGGCCACU

>N186

CGCAGUCAACUACCCAGGUUUGAAGACACACCCUAACUACGACGUAGUGUU

>N187

CUUCCAAGUUCGCCUCCUCCACAAGACUGUUCACAUUGGCCGAAUCCCUUG

>N188

GUGCCACGUCUCUGCCUUCGACCGGACCUUUUUAAGUACGAUAAAUAUCCU

>N189

GAUUUGGAAGUAACGCUCGCCGUAGACAAGUAAGAAUGCCUGCUGUCUUGA

>N190

AUAGGCCCUUCGAAAUAGUCGCGGGACUGCCUGCUAGUGUGGAGCUGCCCA

>N191

AGAUGUUUGAACUGUAUUGGCGAAGACCUAAGAAAAUUGUUAGUGAAUCUA

>N192

GAGGUCCUCACACGUUCAAAGUUAGACUUUUCAUACUGAAGAAUGACAAAA

>N193

CCUGCACCUCCCUCUAAAGCUAAAGACGUAGCCGAAGAUCACCGGUUAAAC

>N194

GAAGCAACAGUCCAAAGAGGCAAAGACAACUGCCGAAUCGACUCAAGUAGA

>N195

GGGAGCACGCCAUGAGAAGCAGAAGACACAAAAGAUAAGGUGUUCGGUUAC

>N196

CCUUAAGCAUAAAUUGGGUUUGUGGACUUAGUCGUUUUAUUAUCAUUGCUU

>N197

UAACUCAGAGAACCAUUUAUCCUGGACCUCGUAGAAAAAUCUAAAGAAUUG

>N198

UGUUGAUACUGGAGGAUGAUUUAAGACGCCAAGCUCACGAACAAAAGAUAC

>N199

CUGUAGUUCUUUUUCAUAUCAGUGGACAAUAUAGAAGAACUAUCGUCAUUC

>N200

UUGAAAAAAUUUCUGUGGCUUAAAGACGAUAAUGCCAUUGUGAAAUUUUGG

>N201

UUUGGGCCAGGGAAGGUGCUAGAAGACGCAAACAAGCGCACGAACUCCGAC

>N202

UCUGUAAUUGUUUUGAAAGAUGUAGACUUGAAGAAACUGCUUAUUGAUGGG

>N203

GUUUAAAUGCUAACAAAUGAGCAAGACAAAUGACCAGAUAUAAACGAGGGU

>N204

AACCUAAUUGUGUUCUAUAUUGCGGACAUAUAUUUUUCGUAGAUUGAAAAG

>N205

AAAAUGUACUUUUUCGUGUUUCGGGACACGUCGCUGAAAAGGAUUGAAAUA

>N206

AACAUACCAGAAAUUUUCCUCGAAGACCAGCAGAAGGGAACCAAUAUCACG

>N207

GGCGACUUCCUGAUUAAACAGGAAGACAAAGCAUGCGAGAGGCCCUGGGUU

>N208

GCACCACCAGCUUGGUACAACUUAGACAUGAUUGGGUUGGCAAUGUCUUGC

>N209

UCAGCAUAAGUGGAAAAGAUCUCGGACUUCUUUGUUGGAAUGGUAGAGUUU

>N210

UUUGUCCAAUUUAGCAUCUCUCAAGACCUUUUCAACUGGGUCCAAAGUAGA

>N211

CUUCUCAAAGCUCUUUGGUUGGUAGACAAGUCCUUCUUGUUCUUUCUCUUG

>N212

GGCAGUUUCCUUCAUCUUACCCAAGACCAUGGAGGAGAUUUGUUCUGGGGU

>N213

GUCCGUGUUUAUGAUGGGAAGUAAGACCCCCGAUAUGAGUGACAAAAGAGA

>N214

UCCUUUUUUGUUCAAAAAAAGAAAGACAGAGUCUAAAGAUUGCAUUACAAG

>N215

CAACAAAGCAUUGAAGAAGACGAAGACCACGUCCAAUCUACCGAUAUUGCU

>N216

UACGACUAUUAAUGACACUCAAAAGACUUUCCUAGAAUUUAGAUCGUAUAC

>N217

AGGCUCCGAAUCCGUAGUCUCAUGGACAACUUUAACACACGUAUAUUCCAU

>N218

UACUCAUGUAGCUGCCUCAUACGAGACCGGAAAUAUAUGCAUUUGGAACUU

>N219

UACAGCCCUGAUUGUUAGUGAUAGGACAGGUAAAGUAUCACUCUAUAACGG

>N220

UCUGUUAUUUCCAUAUCUUCAUCAGACUUCAAUGUUCAGUCCGCUAGCCAU

>N221

AUAUCGCACCAAUUUUUGGUAUUGGACCCCCAACAUGACUUCAAGAUCCUG

>N222

UGUCUUGGUCAGAUAUUACUUUAAGACAUAUUUUGAAAGGCGACUACUUGG

>N223

GCUAGAUAAUAAUACGGAAGAGAGGACUAAGCAACUUAUGGAACCAUUUUA

>N224

GGUCUGGAGUUCUUUGAAUUGAAGGACAACGCGGUAUAUUUUGAAGUUGUA

>N225

GAGGAGAAUUUAAAAGUAAUUGAAGACUUAAUCAUCAUGUUAAAUCCUACU

>N226

GAUGAUUAUCAAACCCCAGUGGUGGACUUGAUAUACAGGAUUUCUAACCAA

>N227

GCUAUGAAAGAUACGGGAAAUUCAGACAACAUCAGGGUACUUGUUGCAAUU

>N228

UGGCAAAGUUACUGAAGUUAUCGAGACGAACUUUGAUCUUCUUCUCUCAAG

>N229

AAUCCUGGAAAUAAAGAAUGAGGAGACUCAGCAAAAGUAUUUGGAUAAGCU

>N230

CAAAUUCUGGACUUAUUGAAUCAAGACUCUAAUUUUGAAGCUGCAGCUAUA

>N231

AUGUCUUCUGUUCUUGAGCACCAAGACGUUAUUUUAAUGAAAGUUCAGGAU

>N232

AAAAAAAUAUGGGGAGCUGGUCUGGACCCAUUACUUUUUCUAGCUUGGGAA

>N233

UGAAUAUUCUUGUUUAAUUUGCCAGACUGAAUCUAACCCAAAAAUAGUAUA

>N234

UUGCCCAGUGGAACUUUGACAGCAGACUUCCUUGCUGUAUUCAAUUUUGUC

>N235

AUUAAAACCCAAGAGGAUAUAUCGGACGGCUCUUGAUUGAUAACAAUAGCG

>N236

GUCGAAGGACUUUCUAUUAAUAUGGACCGGAUCACUGUGCGAAUAUAAUCG

>N237

GAGAUCUCUCUUUGAAUCAUAGAAGACAUGGCCAGAUCGUUGCCAGAAUGU

>N238

ACGACCACCGUUGUCCUACCAGCAGACACUUUUUUGUCUCUCCUUUUGAUC

>N239

CACCAUCAAAUCAACAUCACCACGGACAGUCUUUUUAUCCAAUGUCGUUGU

>N240

CAACAGUGCCUCCGUUUGAACUUGGACUAUCUUCAGCAGAUUUCGGCUCCU

>N241

AUAGUUGAAAUUCCUUUAAGGCCAGACUUAUCUGCAAUGUCAUAAGUCUGA

>N242

ACGAACUGUUUAACUUUUUUAUCAGACAAAUCAAAAUAUUUACCAGAUAUA

>N243

CCCUUAUUUGAAGCAAUUUUAUCAGACACUAUUUGUACGAGUUCGUCAGGA

>N244

AGAACUCUUAAGCACUAGGCGGUGGACCUUAUUAGCUCAAUCUUGAGUCAG

>N245

CCAAAUAUCAUGUUCUACUUCGAAGACUUAUAGCUAAUUAAUUUUUUCAUA

>N246

UGUACUUUUAAACAUCAAAUAACAGACCUUUACAUCAAAUAAGCACCGCGA

>N247

AAUUUCGCCGAUCUUGGAGUUUAGGACGUUAACCUUGGCAUGUAGAAUGUC

>N248

CAGUAACUUGAGCUGGUAAAACUAGACAGCAGGCAAAAACCUGUAAAAGAG

>N249

CUUCCCCUGGGGUUCAAGAGUCGAGACCGAGUCCUUUUAGUUUGUGUAUAU

>N250

AGAUUGUUGCUUACAAGCUCUAGAGACUGGUCAAUAAAACUGUGGGAUCUU

>N251

GUCGCAAGCCUUCUCAGUAAAUCAGACGAAAAACAAUUGAGUUCGACACCU

>N252

GUUGGAACUUCAAAAGGUUGGCUAGACUUCUAUAAAUUCCAUUCUCUAUAU

>N253

UUAACUGCUCCGAUAGAACAAUAAGACAAUACGAAAUAAGUAUUGAUGAUG

>N254

GAGGAGUUGAUAGAUAUAAAUUGGGACUUCUAUAGUAUGAGUAUAGUGAGU

>N255

GGAAGAAAUAGCUAUCGAUCUUCGGACGAGAGAGCAAUAUGAUGUUAGAGG

>N256

UCGUUGGGGUCUUCUUCCUUCAAAGACAUUAAUGUAUUAGCAUCAACACCC

>N257

AUCAGGGUACUAGAAAAUCCCAUAGACAAAGAUUUGCCACCAAAAUCCGUC

>N258

GUUUAUAGUUUGGAUAAUACCGAGGACGUCUACGUUGGAAUUUACUUCCUG

>N259

AUAGUCCAAACGUUUUGGUAUGGAGACAGUUGUUCGAUGGCAAAAAUUGGU

>N260

UGAGUUUGAAUGCAGCAUAUCAGGGACACCAGCAUUGCUGGCGUUUGUUUG

>N261

GUUGACCAUAUCAGCACGCGACUGGACCAACUCAAAGUCAUCUACUAAAAG

>N262

AUCUUGCCGAUGUGAAAUUAAUAAGACAAGAGGGAGAUGAGAUUAUGGAGG

>N263

GGUUAUAGGCGGUGUUGCGGAAGAGACAAAGGAAACGCAUUUCUUCUCAAU

>N264

CGGGAAAGUAGUUGUUUAUCACUAGACAUAUAAUUAUGUUUAUUUAUAUUU

>N265

UGCUACCGAAUAGAUGACAUUUUAGACUUUCAUUGAGAAAUGUGUGGGUAA

>N266

UUUCUAAAUUUCUCUUCAUUUGUAGACUUAAUUAUACUGAUCGUUGAUCUA

>N267

CUAGUUCAUCCUGGUCUAUAUAAAGACCUGGUUGACCUGGAGCGCUAAGUU

>N268

GACGACUCAUGUGUGUUAGAUUGGGACAUGGGAGCAAGUAAAGGAACAUUU

>N269

GGUUGGACUCACAGCUUUUGAAAGGACAUUUCUCGGUUGCUCAGGAUAUAG

>N270

AUGAUAUAUCCAUAAGAGUUUCGAGACGGAUGUAGAGCGUAGCCUGGGAUG

>N271

ACCACGGUCCAUUUGUAUAACCAAGACACUGGCCUGAAACUGGUUUUUAAU

>N272

CACUUUUUGGUAGGUUGUGAACUGGACCAAAUAUGUCAGUAUGUAGGUAUU

>N273

GUCUAACGGAUCUUGAUUUGUGUGGACUUCCUUAGAAGUAACCGAAGCACA

>N274

AAAAAGACCAGGCUUUAUUGUAGGGACAAUAUCAUUUACGAAUAAUUUCAU

>N275

GCCGGAUCGGACAUAUUGGUAUUAGACAAUGCCCUUGAUCCGGAAAGUGAA

>N276

GUUUGAUUUGCCAUUCCUCCACUAGACUCCAAGCUGGCUUGAACUUGCCCC

>N277

UCGUAAUCACUGUCAACCGGUUCAGACUUUUCUUGCCCCACAUACCGAUUU

>N278

UGUAUUCAAAGAUGUACUAAAGGAGACGUCGCUCACUACAUCGCUCGUAUC

>N279

UCGGAGGAAGCAACCGAUUGAGAAGACAUGUUUUCAUUUUUCCAGCAAUUC

>N280

UCUUACCCAGAUUCUUUGAGGUAAGACGGUUGGGUUUUAUCUUUUGCAGUU

>N281

AAAGUUAGAGACAUAUAUGAGGUAGACGCUGGUACGUUGCUGUUUGUUGCU

>N282

CCAAAGUACAAAACGCAACUAGAAGACCGCUCUCUAUUGGUUCACAAACAU

>N283

UGCAUGGUUUGAAACAACCUCAAGGACUUAAAGAAUCUCAAGAGUUCCCAG

>N284

AAUGCCCCAAGACAUUGUCGACAGGACAAGGGCCAAAUAUAUAGAGGCUUA

>N285

AAGCCCUCUAGAGUAAUACUCAGGGACGGUGUCACAUUUCCCGUUUUUAAU

>N286

GUGGGUGUCCCUGCUGCUGCUGAGGACUACGGUAUUCUUGGAAAAACUGUC

>N287

AAACUUGGCUGUAACCUAUCGGAAGACUGUGCCACUGCAAUCAUGUCAGAU

>N288

UUAUGUUUGAAAUGUUGUAAUGGGGACGGAAAAGCCGUCACUUUUAUCUUU

>N289

AACUUGAUUUGAAUUCCUUUUUGGGACAUCCUUGUUCCAGUUGUUGUUUAA

>N290

ACUUUUAUGAGAAAAACACUAUGAGACUCUGAUGAGCCCACUGUAGGAGCC

>N291

AAAGAGGGCAAAAUUUUCUCAAAAGACCAUGGUGCAUAUGACGAUAGCUUU

>N292

GGAUGUUAAAUCGUAGAACCUAUAGACGAGUUCUAAAAUAUACUUUGGGGU

>N293

GUUUAUCAACAGUUGUGCGCGACAGACACUCGAUAAUUGUAUCAGCGAUGU

>N294

CCCAGGCAGCGGGAGCCGCUGCAAGACUGAAUUUAGAGGGUGAUAUAUUUA

>N295

AUUUCAACUACAGUGGCACCUAGAGACCAAAUGUCGCUGAGCGUAGAAGCU

>N296

UCUUUUCCAUACACGACGCCAGAGGACAUUAUUACGUUACAGUAGUUCGCC

>N297

UACCGAGAAGAAAAAUAUCUAUGAGACACGAUAAGGCCCCUUCUGAAUCCA

>N298

UCAGAAGGCCAAAUAACGAUCAAGGACACUCACUCAUGUUUUCCAAAGGCG

>N299

AACACAUAUUUAUGAUGAUAACAAGACGAACGUGUAUUAAGCUCCCAGUAC

>N300

UACAGGUUUCAUUCGUAAAGCAGGGACUCUAGUUUGCGAUAGUGUAGAUAC

>N301

CGACUAAGUCAUUAUUACUAUGGGGACGGGUUGUUCUUGAACGAUGCUAUA

>N302

AAACGGUUAGUAGGAGGGAAAUCGGACUUUUCCCAAAUUAGAAACAAUGAA

>N303

AGAUAUGAUGGGGGUUCUUGUUUGGACAACACAAGUCUCAGAGCCAGCGUA

>N304

ACGACGUCCUUGUAUUAAGAGCCAGACCUCCUGUUAGCGUCACUAUAAGAG

>N305

AAGAUUAGUAGCGCUGCAGGGGGAGACAAUGAGAGAAAUUUCCCGCCACAU

>N306

AUCCUUGUUGAAAAAUGAUUGAUAGACUGGAUUGAGCGGAAAAACAUGGGU

>N307

ACUAUUUCCCCACUGUUGCUUCGGGACGACCCAGUUAUUCAAUAUCUUGCA

>N308

UCUACUGAAUGACCAUAUCUGAAGGACUACCAUAGAGCCACCUAAACAUAU

>N309

GGACGAUAUCAGCUGAGAUUAAAGGACUUUCAGGGUUGUCAGGAGAUCCUU

>N310

UAUCCUGAUAGCUUAGGAGAACUAGACUUGAAUGUGUCGAACAUUUCAAAC

>N311

UUUAUUAAAAGUAAAUCAAAAGCAGACUCGGAAGUUUUGUCGUAGGGAAUU

>N312

UUAUUGUUAGUUAGUUACUGUUAGGACGCUUCGGCGAGCUGAUGUCUGACU

>N313

CAAAAGUAAAACUUGUCCGCCGCAGACUCUAUCACUUAGUCAACACGUUGG

>N314

UGAUAAUAUAACUAUUAGUUGAUAGACGAUAGUGGAUUUUUAUUCCAACAU

>N315

CUAGUUCAUCCUGGUCUAUAUAAAGACCUGGUUGACCUGGAGCGCUAAGUU

>N316

CACUUCUCGGUAAGUGAUGUACAGGACCAAAUAUAUCGGUAUGCAAGUACU

>N317

UAUGUAACUGCAUUCUUCUUAAGAGACUUCUGAAUACUUCGGAAGUUAGCA

>N318

AGAUUCUUUCUGUUGCUGGCCUAAGACUAAGUUCGUUGUCAUCGCUUAAGU

>N319

UGAUAAUGUGAACACGUCAUCAUAGACGGUUGUUGGUAAUGUGCCCAGUUA

>N320

UGGUACUGAAGCAGGUUGAGGAGAGACAUGAUGAUGGUUCUCUGGAACAGC

>N321

GGCUAACGGAUCUUGAUUUGAUGGGACUUCCUUAGAAGUAACCGAAGCAUA

>N322

GUACAAUGCACGAUCCCACUGACAGACAAAACAGCUUCACAGAAUUUGAUC

>N323

AAGUCAGACAUAUGGAGACUCUCGGACUGAAAGCACUAAGGGAUGAUAGCU

>N324

AUGUCUGUCGGCCUGCUAUUUAGAGACAUUUUUUAUUGCAACAACCUACUC

>N325

AAAAAAAAACAACGACAACUGCAGGACUCGAACCUGCGCGGGCAAAGCCCA

>N326

UAGAUUCCAUUAUGGGGAUUCCUAGACCCUCGAGGAGAACUUCAAGUAUAU

>N327

AAACCAUAUAAUAACCUUACACAAGACAAGAUAUCAAUUCAACAUGCAAAC

>N328

CGGUAUAACUGCCUUCGUGUCUGAGACCUUGAAUAAGGGUUCCAUAAUUAA

>N329

AUUUUUCAAAAGCCUUAUCGAACAGACAAGGUCGAAUGCACAUUUGAGUUC

>N330

UUCGAUCACAUCGAAUCCCUUCGGGACGUUGCAACAAUACGUGAAAAAUGC

>N331

GAAGAGAAUGUAGCUCUUCCCGAAGACACAUUCAGUUCACAUCUGAGUUAU

>N332

UGAACCAAUAUCUAUUCAUGAAGAGACUAUGGUAUACCCCGUACUAUUUCU

>N333

AACAACUUUGAUAGUAUUAGAUCAGACCCUAUUUUGAUGGCAUAUGUUUUG

>N334

GAGGGAAAUGUGGUCCUUUCCAAAGACAUGUUCAAUUCGUACUUAAGUUAU

>N335

UCCUCUUUUUUCAUCUCCGGGACAGACCGACAAUAAAGCAUCUAAUAUUAG

>N336

AGAUUUUCAAAAUUGCUUUUCCAAGACAGCGGAUAUUGUAGAACAAUCUCA

>N337

ACACCAUAACACAGCUAUAGAGAAGACAAGAUAUAAACUGGGCAUGCAAAC

>N338

GAGAAAAAUGUGGCUCUUCCCAAGGACAUAUUCGGUUCGUACUUAAGUUAU

>N339

GGCGGUAAAGGGGAAAGAAUGGAGGACAAUUACAUAUAAUAUGAAUCGGUA

>N340

AGUUUUUUUAGAUAGAAGACGGGAGACACUAGCACACAACUUUACCGGGCA

>N341

GAGAAAAAUGUGGCCCUUCCUAAGGACAUAUUCCGUUCGUACUUGAGUUAU

>N342

GCUAGUCAGUCGUCGAAAUUUUAGGACCGAGCUUUUAGUGGAUGUCAUCAC

>N343

UCGAGUCAGCUUCUCCUAGUUCCAGACUGAAUUAUCAAAACUUUUUGCUCA

>N344

CUUAAAAAGACGGAAUAGCUUAGAGACACUACCAUACGUAAAGCGAACAUA

>N345

GCCUCGAAAAGUCGAUUCGUGUUGGACCCAUUUGCUGAACGAAGUGGUUCA

>N346

AGCGGUAAAGGGAAAAGAACGGAGGACGAUUACAUACAAGAUGAACGAAUA

>N347

AUGAUUGAAAGGACUCCCCAUCUGGACUCUAUAUGUCAUCAGCGGCUAAAA

>N348

ACUGUGCUCUCCGCAGAAAACCUGGACGCAUUGAACACGCUGCACGAGCGG

>N349

CCCAACCAUCAAAGACACACCAGAGACGGCGGCACAGGGCCCCUAUGGCGC

>N350

CGGCUCGCCACAGACAUCUACACAGACUCGAUCCUGAGCUUCGCACGCGGU

>N351

CUUCGCGUUGUACGGCCGCUUCGAGACCGUGUACGAGCCUGCCAUGACCAA

>N352

ACCCAUCUUCCGCGACAAGUCCUGGACUACCAUGCAGAACAACGUCUUGAG

>N353

GGUGGUGUCCUCAAGGCAUCGCCAGACUGCUCGGUUUGCGUCGCUCAUGGA

>N354

GCUAGCGCCAACACCAAAUCGGAAGACAUGAAAUACCUGUUGUCCGGCUAC

>N355

CUAAAGAAACAAUUCCAGCCGCUAGACGACCCAAACGUCCAACAAGUGCUC

>N356

GGUGAUAUCGUAGCCUUCCUCCUGGACCAACCAACCAUCAACGACUGCGUG

>N357

CACGGUGCUGACCCCUUCAAGAGAGACCGCAAGGGCAAACUGCCCAUCGAG

>N358

UUACAAAGGCUACCUGAAAAAAUGGACCAACUUCGCUCAAGGCUACAAAUU

>N359

CAGGGCGCCAUAAGAUACGCAAAGGACAGAGAAAUUUUGCUGCACAAUGGC

>N360

AUUAUGAUGAUGAUGAUGAAAGUAGACCCCUCAUAGAACCAUUACCGUUGA

>N361

UCAGACGACGAGGGUUACUCUGAGGACGAUUCUGAUGACGACGGUAACUCC

>N362

CUGGAAAUGGAACUGGUUGAUAAAGACGAAAAAUUGGUUGCCUUGGAUAAA

>N363

GUUUUAAAGUCUAUGGUCGGUCAAGACUUAACCAAACUAACUCUACCGGUA

>N364

CUUGAUCAAGCUGCCACUUUUGAAGACUCCUCUUUAAGAAUGCUAUAUGUA

>N365

UCACCCACCUAUCUCUGCUACUUGGACAGAAUCGCCCAAAUGGGAUUUUUA

>N366

AAACGGCAAAUUCUGCCACGGGAGGACCAAAACUAGAUGGGUCUAAGUUUC

>N367

GGGAACAAAAAGGAGAAGAAUACAGACCUAAGUGGUUUGUCCAGGAGGAGC

>N368

CCAGUACAUCCGAAAACAGGAUAGGACCUAUUUAAUUAUAUAGUAUAAAGU

>N369

CCUCUAGUUUUGGUGGUGAAGCAAGACAAUAACUAGCAAGGGCUCUCACUA

>N370

GUAAUUUUGACAUAUACUGAUGUGGACCUCUUGUUUCGUUUAAAUCGCUAU

>N371

AGAGACUCAAACUAAGAUCAUGGAGACUUCGGCAAGCAAUUGAUUGUUAGU

>N372

UGGCUUUUUAUAUACAGAACAUCAGACGACGGGAAGAGAAAAACGUCAGUA

>N373

GUGAAACAAUGAUGAGGAGAGUAAGACGAUGUAAUUUUCAUAAAGCAAUAU

>N374

UUAAAAAUGCGUAAAAAGAAAGAAGACGUCUGCUACAACCCUCUCAAGUCU

>N375

GCAUCCUCUGAAAAGAAAAAGUUAGACUACUUUAUCUAAUACUUGUUUUUC

>N376

GAAUAUCUGUUUUCAAUCUACGAAGACAUAAGUUUGAAAAAUAUAACCAGA

>N377

UGAAAAAACGGCGGCUCUUUAGUAGACUGGUCAAGCGGCAUCGGAAACAGU

>N378

AUAAGCCCAAUAAGAUACCAAGUAGACAUGUUACACCGUGAGUAGUAAACG

>N379

GUUAAAAGUGAAAAUACCUAUAUGGACUCUUUUGAAGAAUUAAAAGAAAUA

>N380

UGGCUUCGCAGACGAAUGUUUUCAGACACAUGACACUUAUCACCGAAAAAC

>N381

ACCGUGACGGAGAAUACGUAGGCAGACUUUUUCGUCAGUGUGUUGUCCGUU

>N382

UGUUCCUUGUGUUACAACCUCAGGGACAUAUCAGUGCCCUCAAGAAGAUUU

>N383

GACAGGUUAUUUUUUACCACCACAGACGGGUUCUUACACAUUCAAGUUUGC

>N384

CUGGAAACUCGAGAUACUACGCUAGACGAGAUACCUAUUAAUUGUUUUCCU

>N385

CGCCGAUAAAGAAGUAGUACAACAGACAACGUCAAAAUGAUCCUCUUGUGA

>N386

UACAACUGCUGCAGGACUCUUUAGGACUGCAUCAAGGUAAGCCUCGCUGCA

>N387

UCCCAAAACAAUGACAUAUGAAGAGACCAGCAUCAAAAUUUUCAUCAUUAA

>N388

GGAAUUAGGUUCCGUUGGCGGACAGACGGAUUUCUCAAUUGACUACGAUCU

>N389

AUCGUCAAGAAACAAAAUUAUACAGACAGGCGUAAAUGUAGCUCGUAAGCG

>N390

CAUUUUGGGUGCGCCUGGAAGCAAGACCUGAGAAACUGGGCCAAAAUAUUC

>N391

AAGAUUUCUUGUCUCUUCCAUUAGGACAUAGCUAUCUUUUUCUUUUCUGUU

>N392

UACACCAAUAGCACCUCCUCCGCAGACUUGAGUUCUAUCACUUCCGUCUCG

>N393

UACCGCGUUCGACGUUUCUGGUGAGACUUUUGAAGUUUCCGGUAACUUCAG

>N394

GUAAAUAACGGUGAAAUCAACCUAGACAAUGGAAGUACCUAUGUUAUCGUU

>N395

UCUUAACUGCAACCCAAGGUAACAGACAAUUCUCUUUUGAAAUUGGUACUG

>N396

UCUACGAUCUCUUCUAGCGCCCCAGACUCAAUAAUUCCUUCAUCUAGCGCC

>N397

UGACAACAAUGGCUGUAACACCAAGACUGUCACUUCUGAAUGUUCUAAAGA

>N398

UGACGACAACGGCUGUAACACCAAGACUGUCACUUCCGAGGCUUCCAAACA

>N399

AGCGAAGGCACCAUUAUGAAGAUAGACACAUUCUUCUUUUUUUUUUUUUUU

>N400

UGAAGCAAGUGCAAAUGGUUGGUAGACACGGUGAAAGAUACCCCACUGUCA

>N401

AUACUGGUGAGAUGAAUGCUAAGAGACACGCUCGUGAUUUCUUGGCGCAAU

>N402

GCCCAUCAUUCGUGUCCUGCUUGGGACGAUGAUGUCAACGAUGACAUUUUG

>N403

CUACUAAAGGAAAGUGAGGUCCAGGACCAAAAGGUUUGGUUGAGUUUCACC

>N404

UCUAUCAGUUCCACUGUGUCAGCAGACAGGUCUGUCCUGGAACCACAGCAU

>N405

GCCGAAAAGUUGCGUAUUCAACCAGACAGCCCUUCGCGGUAUUCAGAAACA

>N406

AUUACCCCGCCGAAUAAGAAAACAGACCCAUUCACCAACGACGUAUCAAGU

>N407

UUCAUCUUAGAUAUUCGUAGGACAGACUCUUUCCCGUGUAAAUAUUUGUGA

>N408

CACUACUUAAACGAUUCGUUAACAGACGCUCAUUUAGCACCUCACAUAUCC

>N409

UGAACGAAUCAUACAUCUUUCAUAGACUUCGUAUGUGGAGUACUGUUUUCU

>N410

AAGAAUAUCCGAAUUUUAGAUUUGGACCCUCGUACAGAAGCCUAUUGUCUA

>N411

UACUGUUGACGUCUGCAAAUUUGAGACCUCUCCGCGAUUAUCUUGGUGCAA

>N412

CAAAGUCCCACGUUUAAUAACCCAGACAACAUAAAUGUCCUUGAAUAUAAA

>N413

UACGAUGUUCCUCAUAUCCUGGAAGACAUCAAAUAUGGUAAGAACUCUGGU

>N414

UAUGCACUCAUUUCCAUUGGCAAAGACUUGGCGAAAUUUAAUACACUGUAA

>N415

CUCCUUUUUCCCAGGCGAUAAACAGACUAAGCGGAAACUACGAUAUAAUGC

>N416

AGUUAUGUAGGCGUAUUUCAAUAAGACGUAUAUGAUUUAGUCUAAGAAACC

>N417

UUGGGUCGCAACGCAGGGUCUCGAGACCUGAAAAAAGCUUCAUAGCGAUAU

>N418

AACACUUUGAUCAGAGCAGAAGAAGACUCUGUCGAGAUAAGUUUUGAUGCU

>N419

AUUGAAAGUAUAGGCUCAACUAUGGACAAGUCUGAAUUUAUUUUAAGUACC

>N420

AAGUGUUAAUAUCAGAUUCUGAAGGACUAAAAUUUUUGCCUAUAAAGUGGA

>N421

UUAUUAUUCCUUGGAUCAAGGUAAGACAUGGGGUGAAUAUGAUCUAAUCAU

>N422

UACAAGUAUAGAAGGAGGAAACAAGACGCCCAGUGUUUGGUAAAAAAAGCA

>N423

GAAUGUUCGUUUGAAUUUGUUAGGGACGCAAACGGCCUGUGUAUACCAGAU

>N424

ACGCCUAUAUAUCCCACGAUGGUGGACAAACGAUAAAAAGGUUUGACACUG

>N425

GAACAAGGAAGACGAACAAUUAAAGACGAAAAUCACCUUCAAUGACGGUUC

>N426

AACCACUGAUGGAGGAGAGACGUGGACUGAGGUUAAAAAAGGCCCCCAUCA

>N427

AUAGAGUUGUCAACAUCAUUGUAAGACUAGGAUUAUGCAUUUCUUUAAUCA

>N428

GAUUUAGCAGCUGCACGCAGCGAAGACAAGUAGGUAGGUCUUUUUAAACGA

>N429

CAAUACCUCAAACUAAAUAAUGAAGACCCCAAGAAUCCUUGCAAUCGGCAG

>N430

UCCCAUGGCUUCAAUGUUCUGGAAGACGCAUCAACUUCACUUCUUGGGUAG

>N431

AAACCAGCAUUUGGCUCCUCACGAGACCAUUGCAUAGGUGUUCUAGCAUGG

>N432

CCGCUCAGUGACACCAACAACACAGACAACAACUUACCAGAAAUAACACGG

>N433

CGUCAAUCACAGGAGCGUCUGGUAGACCAGCAACCUUGGAGUACAAGCUUC

>N434

GUCCAUGCAGAACCGCCGAAGUAAGACCUCCAAUUGUUUGGAGGAAUUGGC

>N435

CUCGUUCGUACCGUAGGUUGGCCAGACCUUUUCGUAGUUGGCAAUAUCGUA

>N436

AUAUAUGUCUUUUACGUGCCUAUGGACGUCUUACGAAUGAUCGAAAAUAAG

>N437

AGCAGAUCAUACCAUCUGCAAGCAGACCCCAUUAUUGCAUUGCCUCCGCAG

>N438

UUCAAAGUAUCAGUAGCAGUUCGAGACGCUCUAAAGCACUUAAUUCUUAAG

>N439

CGGUAUACACCAUUGAGAGGUUUGGACGCCGUACGUGUCUAUUGUGGGGUG

>N440

UGGCCUCAAGGAAGCAGUCACCAAGACAUUACUUCUCAGGGCGCCGGUAAC

>N441

CGGCUGUUACGUUAUUGUCUCAGAGACGUUUCCUCUUAGGGUCAAAUCAAG

>N442

CUGACGCCAUAGAUCAUGACGAUAGACCAAUCUACAAGAGGUUCUUUUCCA

>N443

UUUUUUACGCCUUAAUCCUUAACGGACUAUAUGUUAUGCCUUAUAGAACUA

>N444

UCCAUUCAAGAAACCAAGCAUUGAGACAGAACAGUACAGAGUUCAGUUGUU

>N445

CGCUAUUUAGCAUUACUUCGAAAAGACGCAAGUAUAAAGAGUGAGAUCUCA

>N446

AGAUCUUUUGAAAAUUAGAAAAGAGACUAAAAUAAAAGUGCACGAAUACAA

>N447

UACGGAAGUAACAUCUAUAUCGGAGACAACUUUUACGCCAAUCACAAUCUU

>N448

UAUGCCAUGCCUGUGACCAUUGGAGACAAUGUAUGGAUUGGAGGUGGAGUG

>N449

UAAGGCCUUUUUCCAGAAAAAGAAGACUGAAUCUUUCUCUUUUAACUCAAC

>N450

GGAUAAGCUCAUACUUACAUUCAGGACUUUCGCCUACUGAGGUGUGUUUGA

>N451

AAAUGCAGUUCCAUCCUGGUAAGGGACAGUUGGAUCGGUGAUAGGAGCAUC

>N452

GAAACUAGCAGGAUAAAUUUGGUAGACGGUGGCUUCUUUCCACCAUUUAGG

>N453

AGUGUCCUAAUUUAUAAUUCGUCAGACAUUUAUGAACUCACUCCUCAAAAC

>N454

UCUAGGAUUGUGACUAUUUCAAUGGACAAUGUUGCAAAUAGUUUGAAAUAU

>N455

AACUGGCAUGGUCGUAAUCUUUUGGACGACGGAAUAUCAUCGCUAACCAUU

>N456

GUACCCACAGUGGAGGAAAACAAGGACUUUUCCGAGUAUAAUGGGGAAGAG

>N457

UACGAUUUCUGGUUUCGUCAAUCAGACUGAUUUUUUAAGAAGAUUUGGUAA

>N458

UUAUUGUACCAAUUAUGGUACCAAGACUUACUCGAAUUCUGUCCAGUGGCG

>N459

AUGGAAGGAAUUAUUUUCGAGAAAGACUAAAGUUUUUCAACGUUUAACGAU

>N460

UGUCGGUAUGAAUGACUCUUUUGAGACUUCAAUUGUCUUGGGUAUUGUGAA

>N461

UUUACGCCUCCGUUGGCGUCACAAGACUAUAUCCGAACGGUAAAAGUGAAC

>N462

UUUUCUGCUUUUCCUGCACCUGGGGACCUGUAUGUUAUGUGAUUAUUUCUG

>N463

GAAGAGGUUGAUGAGAUGUGGAUGGACGGUGUAUUACCUUGGAAAUCUGAA

>N464

AUUUGGUAAUAUGAUACUUAGAUGGACUUAACAACAAAGUGUUUUCUUGAU

>N465

UUUCUUUAUUUUGAAACCCGUCAGGACUACCAAAAUAUAACAAAGUAUAGA

>N466

AUGAGUUGACUUAAUUUUUAAAAGGACGAUGUUGGCGCGUUAGUGCAUAAA

>N467

CACGAACGUCAUUAUUUGUAACUAGACAACUGGGAAUUUUAAAAAAGGUUG

>N468

AAAAUAAUUCUCCUACAAGCUUGAGACCUUUAUUACAAUUCCUACCUAAAU

>N469

GAAGUAGCUGGUCUUCAAAUCACAGACAUUGUGAUGGCAAAGCAGUGAUAA

>N470

ACCGUCUCUGUGAGACCAGUUAAAGACCUCUCUCAGGAAAAAUAUAUAAAU

>N471

CCAUUCUUUGUGAAAACCUACACAGACUACCAACAGAAAUUUUUUUGGUCA

>N472

UAAAAUCGUAUUAUGCAUAAUAUAGACAUUAAUGAGCAAUCUGUUCAUUAC

>N473

AAUUUUCUAACAUUUCUCAAUAAAGACCCUUCGAUAAACAAGAUUCUUGAG

>N474

CGUAAACGACGUAACUUGACGGAGGACGGAGAACUUGUUGAAUAGAGAAGA

>N475

GUAGCUUUUCUUUCCAAUUAAGGGGACAUCGAAACAGUAUAUUAACCAAGA

>N476

AUGGUCCCAGAUUAAUUACGUGUGGACAUGCCAUUUCCCAAACUGGUGGUC

>N477

AGAGAAGAGUUUAGAAGAGGUGCAGACUUUAUUAAGAUUAUGGGUGGUGGA

>N478

CUGAUCAAUUUAGUGCAUUCUUGGGACCUGAAAAUAGUAGAAAAAAUACAG

>N479

UGUAAGGUAGAUAUGGCCCUUUAGGACCCUUAAUUUUACAAGUUACAGAAU

>N480

GAUGAGAAAAGAUACUUUGGCAAAGACCAGAUAAUGGAAGCAUUGAAAACC

>N481

GAUAUUAUGCAUCAUCCAUUCCUGGACACCAACGUUAACCAUACCUGAUAU

>N482

GCAAAAGUAAAUACCACCAAUCGGGACAAUCUUUAUAGUUCUUGGAGUAGA

>N483

UUUACCGUGAUAUAAGAUGCAGUGGACAAAAACGGCGAUAACAGCGGCAAA

>N484

GGUUGACGACCCAUCUUCUGGUAAGACCCGCAGCGCCAUAACCAAUCAUUU

>N485

CGGAUGGUGGGGUCAUCCUCGAUGGACACCGCCGAUCUCACUUCAGGAUAU

>N486

UGCCGAAAACCUUGGUGCCUUGCAGACGGCUAUUGUCCUGGAUUUUGUGUG

>N487

UAGCGCUUGAUAAAGAAUUGUAUGGACAGUUUUGGUCCGAGUUCAAUGCAG

>N488

GUCUCGUUCUACGACUACUUAUAAGACAGUUUGCCCCCGUUGCGGCGGUUU

>N489

GCGAUGCCUGUGGAUCCUCUGGGAGACUGACCGCCUCACCAGUGUACUAAA

>N490

AUAUCAUCAUGGGACGCUGUACGGGACGAUGUGGGAAGCAACCUCUGAAAU

>N491

GUUUAUAAAAAUGGUACCUACCAGGACCUUGCGACGAAUCAACCACAGCAG

>N492

CGGAAAUAUUGGUCUGACAUGGCGGACUACAAAAGUCUUCGGAAACAAGAA

>N493

UUAUUUACAGAUGAUAAAAAGCUAGACGAACAAUAUGCUAAGAAAAUAAUA

>N494

AAAGUUAACGAGGUUUCAAUUAGAGACUCUUUCUAAUAAGGCCCUCGGCUU

>N495

AUUAUUGACUUCAUUACAAAGCAAGACUCUUUAACAAUGGCCAAGGAACUC

>N496

AAUUACUCGAUACUAUACCCCCCGGACAAGCAAUGUUAGCCUAUGGGUCAA

>N497

UGCAAAGAGAAGUAAAACAUACUGGACAAAAUCGAACUGUAACCCAUUCAU

>N498

AUGACGAUUGAGGAAUUGAAGUUAGACUGGAAUUUCAUCUUAAAACAUCAA

>N499

UAUUUUUUUAUUUUAUAUGCGUUAGACUUGUUAUAUUCUUUCAUGCUAUUC

>N500

GAUCUUUUUUGGGUAGAUACAAUGGACCAGUCACGAUUCUUACACUCUUAU

>N501

UAUUAAAACCCUCUCCUACUUGAGGACACAUAUUGGAUAAGUAGAAUGUAU

>N502

CGCGGUUCUGCAGAUCAUGAAUCGGACCAGGAAAACCAUACUUGAAGAACC

>N503

CCAGCACCCAGUCCGACUAAACCGGACAAGAGUAUCCUACUGCACAUGAUA

>N504

AACCUAUAAUUGCUUUUUGUUGAGGACUUAUAGUGCUAGAAAUGGCUUCUU

>N505

UAUGAUAUGCGGCAAAGAAAGAAAGACAUAAAGCAAUUGACAAUGAUGCCU

>N506

AAACUUAGCUUAGAGUAUCCAUUAGACAGCAAGAUCAAAAUGGUUGCAACU

>N507

ACGCAAUAAAUGUUUUUUUCAACAGACUAAUAUCUGUGGGAGAGAUUUUUA

>N508

ACGUAAAGAAAUGAUCCAUAAUGGGACUAAAAUGCUCCAAUAUUUCCUGGU

>N509

UCAGCAGCAACAGUAAUAGCAAAGGACAUUACAGUAGGUGAGCUUCCUUUA

>N510

UCCAUUGCAGGUUCUCAGAGUCGAGACCACCAGCAUCUGUGUCAGUCAGUU

>N511

CAAAGUUUUCAAAGGCGCCUUUCAGACUUGAAGUUCGAGAAAUGAGUUCAU

>N512

UCCGUAAAUACCAUACUUAUCCUGGACCAAAAGCUCUUCCAUAGAACUUAG

>N513

UUUAAAAAUUGUAGCCAAUGAUAAGACAUGAAACAUCCUUAAGUAUGGUUC

>N514

GGUGUAGUCAUUUUGGAGAACAUGGACAAAUUUAAAGUUUCCAUAAUGGAA

>N515

GCGUAGCGGUCAAUUUGGGAAUAAGACCCAGAUCCAUAUAGUAGAAUCAGU

>N516

GAUUCAUCAUACUUGUAAGCACUGGACAUCGAUAAAUUCACGAUACGGUCC

>N517

CUAUCAAUAAUAUCCAGCAUAAAAGACCCUUGAAGUACAAAUUAGAAGAGG

>N518

CAAUGGUCUCCGAAAGAUAUUCUGGACAGUUAUUCAAAAAAACCUUCAAAA

>N519

AUUAUUCACUUCGUAGCUGCUGAAGACGCCGCUUAAAGAGAGAACUAUAUC

>N520

UUUAAGAAUAUCUUAACGUAGAUGGACCAUACUUCUUGUAGAGUUAAAAUA

>N521

GCUGUCCAGCAUUCAAUUAGGACAGACACAAAAGUAACUGAAAAGUCCUUC

>N522

CAUAUGGAAAUCGUAUAGAGCUGAGACAAACUGAUAAACACUGAAUGCAGU

>N523

GUGGAAGAUUUAGAAUGGAACGAAGACUCGUGGCUGGAGUUUGGUGAUGCU

>N524

UAUCAGGCCCUUUUCUGUUUGGUAGACAUAAAAUUUGAGGCAAGCAACACU

>N525

GAGUACAUUAGCUUGUAGAUCCGAGACAUGAUUAAAAUAAGCUUUGUAUGC

>N526

AUUGGAGUAGGACCAACUAUGGAAGACCGCCUGUGUAAACCUUCCCUUAAC

>N527

UAACAUUUAAUUGUCCUUGAAGUAGACACGUUUUAAUAGUGUCCAAAAAAU

>N528

UGUAUGUAUUACUCUUUAAAUUGAGACAAUCAAGGAAAUCACACAUAUAAA

>N529

ACGGAUCAGAAAAUCUGCAAUAGAGACCAGCUUGGGAAAGAGCACAUCGAC

>N530

GAAAUGAUGUAUAACUGGCCAAUAGACUUAAGCCCAACAAAAUAGGGAACA

>N531

GUAAAAUUUUGUAACAUAAAUCGAGACUUAUUAACGAUUUAAUGUUUUGCA

>N532

ACUUGGUUUUGAAUCCGCUUCUGGGACAGUUUUGGAGGUUUCAGUAAUUAU

>N533

UCUAAUAAAAUAUCUUGGAUAAAGGACUCAAUGAACUGUAAUAUUACAGGG

>N534

UCGGCCCAAAAUAAUUCAUCAUUAGACGGCAAAACCGUUAAACUGGGAGGC

>N535

CGAGCGACGGCAGCUAAAUUAUUAGACUGGUAAUUCAAUUCUGACUCUUUG

>N536

AUAACAACGGUAAAAUUUCACAAAGACUUUUUUAGAAUUUUGAGCAGUGAA

>N537

CUUCAUUUGAAUGCACCUGUUGGAGACCCAUUCUAGUCAAUUUCAAUGACC

>N538

CCAGAAGAUACCCAGCUGAAAGAGGACAUUAAAACCACAGUAAAUUACAUA

>N539

UACGAUACUGGAAUAUCCCGAAGAGACAUGGAAGUAAUCAAAUUGACAGCA

>N540

GAACAGCUCACAUCCGCUACAUAAGACAUUCACUGAUUUUGUCGCCCAGUA

>N541

GUGGCAAAAUUUUCUAUCCCUGAAGACACAGAAAUCUUCGAAGGCUCUCUA

>N542

GCGUAAAAUUAGAGCUGUGGGAGAGACAAGGUUAAAGAAAAGUAAAAAAUA

>N543

UUUCCAAGUUUAGUUCAUGUCUUGGACCUGACUUCUUUAUCCGAACUAAUA

>N544

ACAGAUUGACCCCUCUGGCAAUAAGACAGUAUAGGCAGUUAGUAUUCGAUA

>N545

UGAAUCGACUAAACAUGAUUCAAGGACCCUAAGUAAAGCUUUAACUCCGGC

>N546

UCGAGAUAUAUUAAAAAUUUACUGGACUUUUUUCCCGAAAUCGCGAAGGAA

>N547

GAUAAUAUCAAAGAAAUGGUUAAGGACGAGCCAGCAAAGGAUAAAAAUUCU

>N548

GAAGAAAAAUGAAACUACUUCAAAGACAGCAGAUAAAUUCUCUCAAAAGGG

>N549

AACUAGCUUAUGACUAUUAAAUCGGACUCUUAAUUUUUAGCUAAUAUCUCU

>N550

UUGAAAAAAUCGAUUUGAUCUUUAGACAUAGUAUGUUUUGCCUUGAUGGUG

>N551

UUUGUCUCAUGAAUUCUUGCAAAAGACUUAACUAAAAGAAUCUCUCCGCCU

>N552

UAGAUGGCCUUUAUAUUUCAACCAGACGCCCGCAGCGGAAAUAUGAUCUGU

>N553

GGGUUGAAAGGUCUGGUGUAAAUGGACCAUUAACGUGAGGUUCAAUUGCGG

>N554

UUUGUGAUUACAUCUUUAGGAGUGGACCAUCCGUUUAACUUUCCGGUCAAU

>N555

GGGAAUCAGUACCUAGCAUCAUUAGACCUGGAGCUGAGAAAUUUUCCAAAA

>N556

AUGCUGGGACAGAUGUUUGAUUUAGACCGGUGGUCAUGAAUUGUAGAAGCG

>N557

AAGUAUUUAUUACCACGGAUGGUGGACAGAUCAGAAGAAGUAAUCGAUUCC

>N558

AACGUAAAGGAUACAAUGUUAUUGGACUAAGAAUCGUCUAUCAUCUUAAGA

>N559

UAGAUUCCAUUUUGAGGAUUCCUAGACCCUCGAGGAGAACUUCUAGUAUAU

>N560

CCCAUUUCUCAGGGUGGAUGAUUGGACAAUUAUUGAGAAGGUUUCAUUGUG

>N561

CUUAUAUAUUGUUAAUGCAAUCGGGACAGCUCACUGACUGAACGGUGAAUA

>N562

CCGCUGGAUACUCUUCUCUAUCAAGACGUAAAGCGUACUUGUAAUCUCUAA

>N563

CCAGAAUGGCAAAACCAUUAUAUGGACUACAGCGAGCUGAAAAAUCUUAUU

>N564

GUUGCGAGAGUUGCCUACUGCUCAGACGGUCGCUGCCAAACCUUCUCCUUU

>N565

CCACUGGACUUGGAUGAUGACGAAGACGACGACGAAUUUUACGAUGAUCAA

>N566

AUCGAAUCGGAACAGUUUUUCAAAGACACAUAUGCAUUCCAGGCAGAAACG

>N567

AUGUUGGGACUGCUAUCGCAAGCAGACGAGUUGACACCAAAGGAAACAGAA

>N568

CACUGGACUCUUGUUAGGAAUAAAGACCUUCAACGACGCUGCUCAGCACCG

>N569

AUUGGAAAAAUUUAAGCCUAUUAAGACGAAAUUCACAGUUAAGCAGUAUUA

>N570

CAUUAUUGUCAUCCUGUGGUAUGGGACUAGCUUCUUCGGGGUUCCCCAAUG

>N571

ACACCAUGUGAUCGGUUGGGAGUGGACUUGAUGAAUGUUCUAGAUGACAAG

>N572

AACAACAAAGAGACGUGGUUGCUAGACUAAUAGAAGAAAACAAGGAAACGC

>N573

AUUAAUACACGCAUGAUUUGCAGAGACUUUGAAAAUUUUGUGCUUGAGGAU

>N574

UUUUUUGAGAAAGCAGAUCAUCUAGACGUGAAUGCGGUGGAUUUUAAAAUU

>N575

CCAGACAUGUUUGAAAACCGUUUAGACAAGAUAACUUCAAAUCCGAGUGAC

>N576

UAUUUCCUUUAUGAUGGUUAUGAAGACGAAAUCAAUGAAGAAAAUCCUCUU

>N577

CAAGAAUUUUUAGCCUUCCUGUUAGACAGUUUACAUGAAGAUUUGAACAGG

>N578

UGGAAUGUAGUCAAGAAACUGGCAGACGAUACUUGGGAGAUGCAUUUAAAG

>N579

UUAAGCAAAUCGUCCACUUAUAUGGACUUGAAGAAUUAUGUUGGUAAAAUG

>N580

AACCUUGACAAAGCUACAGAUAUAGACGAUAAACUUGAAGAUGUGGUGAAG

>N581

GCUAAGGAAAGAACCAUAACCUUGGACGAUUGUCUCCAAUUAUUUUCCAAA

>N582

UUCGCCGUCAUAAAGAUGGCAAUGGACUAGGCAGCUCUAAACUACAGGAAA

>N583

AGAUGACAAUGAUGAUGGUGAGAGGACAAAUUCGGGUAGGAGAAAGUUAAG

>N584

AAACAGAAAAAAACAAUACAGAAGGACUGGUUAAUUGUUUUUGUUGAUCUU

>N585

CUUUCUAUGUUUCAACACCAUCAAGACAGUGUCUGCGAACUCCACAAACUU

>N586

ACAUGGCAAUAAAAAGCAGUACCGGACGAGGUUCACUCAAAGGCUGCUUCC

>N587

GUAAAGCCGACGCGGGUAAGCCAAGACCCUGAGUUUAGAACUACUAUUUCG

>N588

CGCCACGCUCGAAGGACGUACUGAGACAUACGAAUCCGAACUUGCAGAAUU

>N589

GCUAUUGCAUGCCAACACGAGCGAGACACAAUCAGUUAGAACUAUUCUUGA

>N590

AGGUUUCUUUCCAGGUUAAAUUUGGACAGAGGGUUGUUACCGCAAACUAUA

>N591

UUGUGGGCUGGUUUCAAUCAAAAAGACAAAGUGUAAAGGGAAAACCAAAAG

>N592

UGCAUUCAAAAAAAUUGCAAGAAAGACACAAUAAAACGUAAUUUGUACAAA

>N593

UGUCUUGCAAAAUAUAUAUGGCAAGACUGUGUUCACGUACAGAUCUCAAAC

>N594

AGUCUCUUUAUUUUUACUGAACGGGACAUUCCACUUCAUUCAGGCAAUGAU

>N595

CCUGAAAUCCUCGAUCAUUUUCGAGACUGUUUUUCGAGCUUGAUUUUAUAU

>N596

UUUCAAACUUUUUAAUGGGUGAUAGACUUGAAAAACAAAACGGAAUAAAAG

>N597

CAUUUUCAUCGCAAGAAAGAAAAAGACUGAAAUCAACAACUCCCAAUAACA

>N598

UAGUUAUUCUUUUUUUCAGAUGGAGACUGUGAUCGGGUAGCGCAUGAUGUU

>N599

CAACUAUACCUCUUAUCAUGUAUAGACCUUGUUCAAGCUCGCGAUAACUCU

>N600

GGUAUGAAAGUCAAGGCCGACAGAGACGAAUCAUCUCCAUACGCUGCUAUG

>N601

GAAGACGUUACUCCAGUCCCAUCAGACUCUACCAGAAAGAAGGGUGGUAGA

>N602

UUAUUUCACAUCUUCCAGCGAGUAGACAUGCCGCUGGUAAUCGCGCGUCCU

>N603

AGGCAAACAGUACGCCUUAGCGGGGACGCCGAAGCGACUCCUUCUGUUCCA

>N604

AUAUUAUACUGAAAAUUCGAAAAAGACAAGCAAAUAAACACAGAUAGAUCA

>N605

UGGCUAAGGCUAAGAAGCAAAACAGACCAUUGCCACAAUGGAUCAGAUUGA

>N606

AGGUCUUACAUGGUAUUACUACCGGACUGCAAUUACACUCUUUUUCGUUAU

>N607

UUUUAGAACUUUUAUGCAAAAUAAGACGAUUCUUGGUAGCGGCAGUAUUGA

>N608

UCGCUGCAAUUUCAACAAUCAUCAGACCCAGGGAAAAAAUAUCUGCCUUGU

>N609

CUGAUAACUUAUUUGAAGAGAUUAGACGCCUUUUGGAAUUUAACGGCGAAG

>N610

UUGGUAACCAAGCUAUUAGUGGGAGACAGGAUGUUUGUCUGGUUAUUGUGA

>N611

GACAUUUUAGAUACGAGGCCCGAAGACGUAAAGGCUGAUUGGUUAGGUUUA

>N612

ACACUUUCAUUUUCAUGGAACGGAGACCACCUUUUAAUACGAGAAUCCACG

>N613

AUACAUACAAAAAAGAGCCUCUUGGACAACCUUUACUUAAUACACAGGGGC

>N614

GGUGCCACAUUUCUUUCCUUAGAGGACGAUGGUUGCAAGAUCAGCACAAAU

>N615

UCUUUUGGUUGCCAAGUCGGAAUAGACGACAGAUGCAUGUUUCGGUGCAGA

>N616

CGAGAAGUGCACAGUAAGUUUACAGACACCUUAUCAGGGAAACUGAAUUUU

>N617

AUUGAAGUCAUCAUUCCGCCUCAGGACGAAGGCGAGGAUGAUUUUUGUAAA

>N618

UGAUAUUGUGGCCCGAUUUUUGGAGACGUGUCACCCCUCCCGCCUUUUACA

>N619

CAUGAUCUGGACGGCACGAUGCCAGACUUGAGCUCUGAAUCAGGCCAGAUG

>N620

GCAUAUGCCAUACUAUCAAGUGAGGACUAAAUUUGAAUUUGAUGGUUUCUU

>N621

CAGUUUCUACAAGGCAGAUCCAUGGACUCUUUAUCAUGAAAACAGAUUUAU

>N622

GAAAAAACUAACUGAUGGUAUUCGGACAACGUUUUAGAAUGAGGGUAUCUA

>N623

CAAGAUGUGUUUUCCAACGACGAAGACGCUGCGCUUGGUGAGCGGUUUAAU

>N624

UCAUCGAUCCGGUCAGAGUUACUAGACGAUCGAAUGUUACGAAGAGCGGGC

>N625

UUAAUUCUGGGUUGAAUGCGAACAGACCUUGACCCGCAAUCAACCUGGUAA

>N626

CGCACGCAGCUGACGGGGCUACAGGACGAUAUAAACGAGUUUUUGACAGGA

>N627

CUAUUAGUGAAGCACUCGUUCUUAGACCAUGGUAAAUUAGUGUAUCGGAAG

>N628

UGUUGUGGCGAAUUCAAACAUCAAGACAUACAAUCAUUUUGAAUAUAGUUC

>N629

GACUUUUAUGGUAAAGCUCUGAUAGACUACUUGAAUGAUCCGUUACUGAGA

>N630

GUGGCAUCCUACAUUACUGGGUAAGACGGCAAUCGUCAUACCAAAAGUUUU

>N631

GAUGUUUACCAUUAUAAUGAAGGGGACUUGGCUGCCUCCUUCAAGGGAUGU

>N632

AUUUUGGUUAUUAUACCCUUCCAGGACAGUACGCGCAAACAAUUAUGGAGA

>N633

AAGUUUAAUGUUUCCAUUGUUGAGGACUGGAGAUUUUGAAAUCUGUCGUAU

>N634

AAUAAUGGGCUAGGACUAAUUGUAGACAGAAAUAUUCCAACAGAUGAUUUU

>N635

CCUCAUUAUUUUUCAGAACACAUAGACAAUAACUCGAGAGAUGUGGUAAUA

>N636

GGUAUCUUGAAUAUUAGGACGAAGGACGCUGACAAGUUGCUUCGGGUAUUA

>N637

CAGGACGUCAUCGGUUCAAAAUCAGACCUAAUCAGUAAUAUUCGUCAAAAA

>N638

AAUAAUAGCACGCCGCAAAUGAAGGACUUUGUGGUUAAUACAGUUUUUUCA

>N639

UCUAAAAUUUGUAGUCGAAUAUUAGACUCAGAUGAAAAGUUGCGUAAGAAC

>N640

UUCAAACUCAUUCAUAGGGGAAAAGACCAAUAAAGUAUCGGUAUCUAAAUA

>N641

AGUUUUAAUAGGUAUGCUAAGAGAGACUUUGCAUUAUCAACUAUUAUUCCA

>N642

AAGUAACAGUGAAGAAAUUUCUCAGACGAGCAGAUGGGAAUUAAAGAACUA

>N643

AAAGAACUAGAGUCUUAUCCAACGGACAAAGUAUGGAGAUCUUGCGGUAAA

>N644

GAAACUACUGUUGAAAAAACAAUAGACAAUCUAAAGGCAUUGAUGAAGAAU

>N645

UUCAAUACAUCAUGCUUCUCGCAGGACAAGGCCUGUACCACUGUAAUUAUA

>N646

GGCAUCCUUUUCAAUUUGCACCUGGACAUUCUCACCCAUACAUGGCCUUGU

>N647

CAAAAAUACUUGGACCAAGUUUUGGACCACCAAAGAGCCAUCCCAUUCAGA

>N648

AGAAAAGGAAUAAAGAAGAAAUGGGACAUGCAAAAAGGAACUAUGGAUACC

>N649

AGAAGGUUGUCAGAUUGACUUCUAGACAAAGAGGUAGAAUUGCUGCCCAAA

>N650

UACUACUAUAUACUAAUUUGUUUAGACCAAUACACAGAAUCAAAAUCAAAC

>N651

UUUUGUGAUUUUAUUGAUUCUACGGACUGAAUGGCUCUCUGGGUCAUCGAC

>N652

CUAGAAUUUUGACUUUUUUCAGAGGACGUGUAGUUUCGCCUUCUUCCAAUA

>N653

GCAUCAUGCCUUGUGGAAUAUUGAGACGUUAGUGGGGGUUCGAUGUUCUUA

>N654

GAAUAAUGCAGCAGCAUCACCAGAGACGUUUCUCCUCGCCGUAGUGACACU

>N655

UGUCUCCAAAAAUGGAAUUAUCAGGACUAAUCAUCGGUUGAUCGUCAUCCU

>N656

ACUAUCCUCAUUAUCUUGGCCAAAGACGUUCUGGGCCCUGCUUUCUUCUGU

>N657

GGAACAGUUGCUUCAUCCUUCAGAGACUCAUCUUGUUGCUGCGAUUCUUCA

>N658

ACGUCCACGGAAGUGUUUACCGUGGACCCCUCACCCAGUGUAUUCUCCAUG

>N659

UACAUUCAAGUUUUUGCCCAGGUGGACGGUCAAGGUUACACUAUCCAUUAU

>N660

UACUAAAGUGACCGCUACCACAUGGACAAGGAAAUUUGCCACUAGUGCUGU

>N661

AACAUGAGAAAAGUAUGAAAAAUAGACGGCUUCUACUAUCAUCAUUACAGU

>N662

CCAUUUUUACUUGUGUUACUGGUAGACGAUGUUCUACAAGAAUGGUGAAGG

>N663

ACAUAUAUAUAUAUUUAUGUAAUGGACCUCUGAAUAUUUUACUAUGUAACU

>N664

AUCUUGAUGUUGCAAAGAAGAAGAGACUUGCUGUUCCCUAUUGAUCACUUA

>N665

GUAAGAUAAGAGUUUACUGAUAAAGACAAAUAAUUGGGAUGACCUAGUCUA

>N666

GUAAGAUAAGAGUUUACUGAUAAAGACAAAUAAUUGGGAUGACCUAGUCUA

>N667

UGAAUUACACGACUAUUUUGAGAAGACUUUCCCUAAUAUUCACAAGCAUUU

>N668

GCACCAUUCUGUCCUAGAAGGAAGGACAAGCUUGUUGAGUACAUUUCCAAC

>N669

UCGCCAAGUUCAGGCCCUGUUUGGGACAUUUUGGCAGGUACUAUUCAAGAU

>N670

UAUCAUUGAUAUUGAUUUACUGAAGACACUGCAUUCGGUUAAUGAACACGU

>N671

UGGAGUGGCAUCAGAUAUGAAAGAGACGACAUUUCCACUAGAAUCAAAAAC

>N672

AUCAUAACCUGAUUUUGGACAUGAGACAUUCGAAUAGAUUAUGUAUUCUUG

>N673

UGGAACUGGACUCAUAGUAGGCAAGACGGUUCCAAUCAGAAGAACUUCUGC

>N674

CAACAUAAACAUGCACACGGAAAGGACUAUACUCUGUAACAUUUGUCUUAU

>N675

GACAAAAGAAAAGGGCGGAGCCAAGACUUUGAAAAAAAAAAUGGAAAAAGG

>N676

GAAUCGAUGAGGAGGCCAAGCAAAGACCGUAAAUUCCAGUCGCGGAAGGUC

>N677

CUGCAUGACGCGAAUCUCUUGGAGGACUUCUUCCUUUAUGGGUAUCACCGU

>N678

AAAAAACCUUGGCAGCCAAGGUUGGACAGGCACACUCAUUUUACAUGAUUU

>N679

UGUACAAGCACAAUAUAAAUGACAGACUAGUCAACGACGCUGACCCUUUAU

>N680

UUCUAUUGACAGGAAAAAAAUAAAGACACUUGAAACGCACGAUUUUUUCAA

>N681

GAUGAUGUUGAAGGUGGAGGAGAAGACAUCCUUGUUGAUGCUGAUGAUAAU

>N682

GUGCUCUAGUCUUUGGGACUGGGAGACCGUUUUUCUUUUCAACCGCUUAGC

>N683

UUGUGUAUUUGACCUUGAACUAAAGACCUUAGAUGAUCCCGAAGGUGGAUA

>N684

UAAACGAAACGCCUUGUUGAAAAAGACCGAUAAAUAGAGGAAGCAACGGCA

>N685

UAAAAACUCAACCAACAGGUAUUGGACUGACAUAGGCACAAUAAACUCAAA

>N686

CACGUAUGCUAUUCUCUCCAACAAGACCGUUGAACAAUUGGGGCAAGAAGA

>N687

UUCUUGGUCGCCGAUGAUAUGAUGGACAAGUCCAUUACCAGAAGAGGCCAA

>N688

GCACUCCUUCAUAGUUACUUUCAAGACUGCUUACUAUUCUUUCUACUUGCC

>N689

UUCCAAAUUCAAGAUGACUACUUAGACUGCUUCGGUACCCCAGAACAGAUC

>N690

UUAGACGAAAAUUACGGUAAGAAGGACUCAGUCGCAGAAGCCAAAUGCAAA

>N691

ACAUUAGAUUUCAAAUUAGAUAAGGACCAUGUAUAAGAACUAUAUACUUCC

>N692

ACCCAUGGCUCCACUCUGGAAGUGGACGUGUUUCUCCCAGGCAGUCUGAAA

>N693

AGUCUGCUUCUAAUGGGUGCAAUGGACUCCCAGAACCUUCUUUAACAGUUU

>N694

UUUCAAAAUGUUUGGGGCAUUGAAGACAUGUUUUGGUGGAGUGGUUCUUGA

>N695

AUUAGAGUAGGUAAUGUAUGGUAGGACAUCUUUAGCAGUCUUGCACCUUGC

>N696

AUGUUCGUGGUAUCCGUUUUCAAAGACUUUGAAAUGACCUAAAUCCAAAAU

>N697

GAUUGCUAUGAUUACGUACGCCCAGACUGUUUCUCCUUCUAAGUUUUGAUU

>N698

CUGAUAUCCCUGAAGGCUUAAAUAGACCCUUAAUGGAUUCGGAGAGCGAUC

>N699

AUGGAAAGAAUGGAUCUUUUCGUAGACUAUAAUGCUUCCUAAAAUAUACAC

>N700

CGGCGAGCAAAGUAGAAAAUCAAAGACAGAAGCGUAGAAUAAAAGCCCUUU

>N701

GUUUCUAGAUAAUAAUUUUUCCCAGACAACUUCUUUGAACCAGGGGUGGUU

>N702

AUUAUGUCUUGAUCUAAUCAAAUGGACCCUACCAAAAGAACCCGUACCCAG

>N703

UCCUCACCUUGUCUAUCAUCUAGAGACUUUUGACCACCUCCAUUUUGUUCU

>N704

UCAUGUCAUAAGCAAAGUCGUUAGGACACUGGCUUUGCAAGAACGAAUAGC

>N705

CGCCAAAUCGUAUCAUGACAAAGAGACUCAGUGUUAUAACAAUCAUAGAAA

>N706

AUUACGGUAUGCCGGGGUACUAAAGACCCUGCACUGUCGGGCUUCAGCCAU

>N707

CCAUGAGUCCACUUCACCAGAAAGGACCCAAACAUUGGGCCUACGCCCAUA

>N708

CUUCAUUUUCGACGAUAGGGUAAAGACGUGCGUAAGAAGCCCUAUGACCCU

>N709

GGUAUUGUAGCAGUCAUAGAAAUAGACUCCGCAAUACAGAUAAUCAUAACA

>N710

AAUAAAUAGUCCAUCUCAUCUGAAGACAUUAAACGAGUUAUUUCAUCCUCG

>N711

UUUGUAUUCCUUAUAAGUCUAUUAGACUUCUUGCAGAUCAGCCCUUCAUUU

>N712

AAAUUAUUUCCAUCCUGGCGUAAGGACGACGGGUAUAUGCUCAUUAAGAAG

>N713

UUUUUUUCUUCAUCAGGACUGCUAGACUUCGACGUCAUUCCAUAAUGUUGC

>N714

CGUUGGUAGCUUUUUAAAUUGAAGGACUUGAAUAUUGGAACCUUACUCUUA

>N715

AAGCUUCUGAUUGACCAAGUGUGGGACUAGCCCCUUGUGAUUCCUUUGCCU

>N716

ACCACAGCUUUGAAUAAUUCCUCAGACUUAUCAGAUUUUGUUUUAUCUGGG

>N717

AGCACAAUGAUCAACUGAAUUGAGGACAAACAGAUAAUUUUCGUGUGAUUU

>N718

GAUCAUCUUUCCCAUAUGAUAAUAGACACCGGCUCUAAACUCAACUGUUCU

>N719

AAUUGGAAUCUUAACAGCAACAGAGACUCUCUCAAAACCGAUACCAGAACU

>N720

CAUUACAUGCCAUAUCUUCUUCUAGACGACUACACCUCUAAUUACAUCAAC

>N721

CCGAUAUUCGCAGUCCACCAUUCAGACCUCUGGUGAGAUAGUUUGCCUGCU

>N722

GAAGAUCCCCAUGCCAUCGCAGAAGACGACAUUGUAAAUAUAGUCCAUGAC

>N723

AAAUAGCGCCCGCCUGUGGAGGUGGACCAUCAAAUUGGAACUGUCUGUUUG

>N724

GUUUUACUCGGAACAGCAAUUGUGGACUUUGUGACAACAUUUUUAGCUGUA

>N725

UAACGGUGAAAGGUAUAACAGCGAGACCGAAUGAAGUCCGGUACUCUGUUG

>N726

AGUUGUAUAGAACUAAAAAAUCGAGACUAAACAGAAAUUCCGGAACAAGAA

>N727

UUUCCUAUUUCGGAAAUUAUUAAAGACAAAAAAGCUCAUUUAUGGCUUUCC

>N728

UAAUAAUAUAAUGCAAUACAAAAAGACUUUGGUUGCCUCUGCUUUGGCCGC

>N729

UACCACCAAGACUACCUCUGCUAAGACUACCGCCGCUGCUGUCUCUCAAAU

>N730

UGGUCAAGUUCAAGCUACUACCAAGACUACCGCUGCUGCUGUCUCUCAAAU

>N731

GCUGCUGGUUGGUCUAUAACUCCAGACGGUAACUUGGCUAUUGGUGACAAU

>N732

CACUUGGAAGCUAUCGAUUUGAUAGACUGUUAAGCAGAAAACUAUUAGUUC

>N733

AAUAAUCUUAUAAAAUGUUAAAAAGACUUGGAAAGCAACGAGUGAUCGUGA

>N734

AGAUGGCGCCGGCUUGUGGUGGUGGACCGUCAAAUUGGAAUUGUCUGUUAG

>N735

AGCAGUAGCAGCAGAAGUUGCUUGGACUUGACCGUCACCAAUUUGAGAGAU

>N736

AUGGAGCCGGUUGGGGUUAAGGUGGACCAUGGUUCACCUGGAGUGUAACCU

>N737

UGCUAAAAAGAGAAGGUUAGGAUAGACCCACCACAAGAUAGAUUGGCUCUC

>N738

UUCUUAUUUUUGUUUUUGUUUGUGGACGUUUAGCUUAAAUCGGCGCGAAUU

>N739

ACCAUGUUGGUAUAAUUGUGUACAGACACGUCCAUUUUUGGUACGAAGAGG

>N740

UAUAUUCAGUCAUUGCGUAAUAUAGACGUGGAGAAACGAAAAAAAAAAAGG

>N741

CUUGCUCGAAUGUAGCUUGUGGUAGACGAAGAGUUACAUCUAGAUAACGCU

>N742

GUCAACAACGUCACUCCCGUUGAGGACCAAGAUUUGCUUGGCGUAUCCAGU

>N743

AUACUGUUGCUUUUCAUCGCGGAAGACGUUUGGAUUAACCACUUCUAAAUU

>N744

CACUGUGCAAUCUUUUCCACGGUAGACGAGAAUCAUGGGAUUGUGCUGAUA

>N745

GUGUCUCCAGUGAAGAAUUUUCUAGACAAUACUUCCAUUUGGGAAGCAACC

>N746

GUCCACAUGAUUCACAUCUUGCAAGACCCCUAAAUCAUCACUGGUCCGCAU

>N747

AUUGCUGUUGCAUAUUGGAUGACGGACCAUCAUCAUGAGCGCCUGCGCCCG

>N748

AUUUCUCACCUAUGUACCUUUUUGGACUGAAUUGUGAAUUGCAUUUUUUUA

>N749

GGUUUAGGAAUAUUGCCUCUUUUGGACAUCAUUAGGCCAAGAUUUGGAGUA

>N750

AGAUAAGAUAAUAUACACUCUCAAGACAGAGUGAACAAAAAGGAUAGUUUC

>N751

ACAUACCAACAAGCCCCAAACCUAGACGAUCUUCUAACUUGGUCAAGAUCC

>N752

UUAGCGCUUGUCUCAGUGGAUAAAGACAUAGAAAUUGGCAAUUCAAGUGGA

>N753

GUAUCGAUAGUUGCUGGGGUAUGAGACGGAAUACUGCCUACUGCUGAUGCA

>N754

GUUGCAAUGGCUGCCAAUACAAAAGACUGAUAAGCAUGGUAAAACGCCCGA

>N755

AUUUUAUUAAUUUUUCGUAAUGAAGACGAUUUUUCUAAACUUAUCCUGUAC

>N756

UUUCCUGAAGUGGCUAAUGUUGAGGACGUUGAAGAGCUGACGUCUAAAGAA

>N757

UCUUCAUUUCCAGAUACGUUGCUAGACGACGUUUCUGCUGCGUUUUGUUCA

>N758

GCGUAUCAGCCACAAGUAAGCUUGGACUUAAGAUACACAAAAAUAAAAAGU

>N759

AGAGCCAGAUAUAUUUUGGUUUAGGACGAAUAUUCAUAACAUAAAAUACAA

>N760

CUGAUAAUUUUUUGGGAAAUAAAAGACUUUCCUCUAGCUGGUAGUCCAACC

>N761

UAUUUUAUCGAUAUAUGAGUUUCGGACUCAUCCCCGUGAAUAAAAUAGUAG

>N762

CCGCCACACGGAGAGACCUUUGUAGACAUUCUAACACCCGUUUACCAUCUC

>N763

AACUGAGUCUUUGAGUCACUCAGAGACUCUUCAAAUAUUGUGAAGGCCUGU

>N764

UUUCAUUGACAACUUUUUUUUGUAGACUAAUGCUCUGCAAAGCCAGGAUGU

>N765

ACCAUAAUCCUUAGUUUUUUGUAAGACUAAUUCAAAGAGUUUGUUCAGAUU

>N766

AUCUGCGAAAGACGUACUAGUUGAGACCCCACUAAAAUCUGCAGCUCUUUC

>N767

AUUCCAUUAGUUUGUGUUGAGAUAGACACCGGUUCAUUAGUGCGGUUCGCU

>N768

UUAACUGGGUCCACCUUCUUAUAAGACACUCUUGUACAAAACUCAGUCAUG

>N769

UCCACUAAGAACUGGGCCAGAACAGACUUCAACUUUGUUUGUCCCGACUUG

>N770

AUCUCUUCAUGGUCAUUCUUAAUAGACUGCAAGAGGUUUUCCAUGGUGUCA

>N771

UGCGUUAUUGAUGUCCCAACCAGAGACGACAAAGUCGUUUGGGCUAACCAU

>N772

GGGAAUGAAACAGAAUAAAUCAAGGACAAAACUAGCAUUAGGAACCCGACA

>N773

GGAAAGAAGAAUGAAGAUAAAAUAGACAAUACUUUUCACAUGCCGCAUUUA

>N774

CAUCGGCACGUAUUUCAUAAAAAGGACGUGAAGAACAAAAGGAACGAAGAA

>N775

GUUGAAUAUACCCUUACGCUUCCAGACGGCCACUGGGGGAAUGAAAACCGC

>N776

UCUCUUUCCUUUAUAUACUAGUGAGACCUUUUCCCCCGUUCCCAAAAAGAA

>N777

CUGAUUUCCGUGCAAAAUAUCCAGGACGUCUAUACACAGUGUUUACAACUC

>N778

GUAUUCUCUCAUUUGCCGCAACCAGACCGCUUGCAGCUUUGCCUUGUGAAC

>N779

CUUCAUGCAUCUCGCAAUUAAUUGGACCCUACUGAACUUGCCGUCUUCGUU

>N780

GCAAUCAGGGCAAGUCCUUGCAAAGACUAGAAAACGUCACUGACCCGGCUU

>N781

GAAUGACACUUUGUUAAAACGCGAGACAGAAAUGCUGAAAGAUUUUAAAGA

>N782

UUAGUUCGUUAACCAAUUUGAAAAGACUAAAAAUGGAUUUCUUAGAGGAGU

>N783

GACUACUAUAUUCACGGUCUUCAAGACAUUGCUGCAAAGGAUAUUUUUAAA

>N784

GAUACUACAGUAUAUGAUCACUCAGACAUCCUAGGAAGGUCUCUAAAGAAG

>N785

GUCGAAUCGGACGAGGAAAGAAGAGACACCAUUAUCAUCAGGGCCAAUCAA

>N786

UCGAAAGAUUACGUAUCAGAUUCAGACUCUGAUGAUGAAGUGAUAUCAAAC

>N787

GAUAAACACGAUUAUAAAAUCAUGGACGAUACAGAUAUUGAGUCCUCGUUA

>N788

UUGUAUUGGUCCAUUAUUGGUGUGGACGGUUUCUUCGGUGACUCCAGCAUU

>N789

ACCAUCAUCUUUAUUGUGGUUCAGGACCGUUAAAUCAGGUAAUAUAUGUGA

>N790

GGAAACGGUAGAUCACUGUAAUCGGACAACCAACGAGGCUCGUAGUCUUUU

>N791

AGCAACCUAUCAUAUAGAUCCUCGGACGAUGAACCUUGUAAUUCCUUCGAA

>N792

UGAACAGCCACUUUUUGUUUCUUGGACUCUACUUCCGGUAGAUUUGAAUUC

>N793

ACAAAGGCCCCAACAACUUCACUGGACCUUUGGUCAAGUAUUUUAGAGUUG

>N794

AUUAUUGUAUGACAGUUGAUAUCGGACCAUCAUUUUCGACACUUUCAUUGA

>N795

CGGAAAUUUCCCCAUGCGCUGCAGGACAGAAACACAAGCAUGAUUGAAGCA

>N796

ACAUCAUUAUUCUUUGGGACAGGAGACGAGAGAGUCUGUUUCAGAUAUCCU

>N797

CUAUACAGGACGUUCAGUGGAUAAGACAUUUACUGAACCCAAGAAGUUCGU

>N798

UCAAUUGGCCCAGGUAGGAAUCAAGACGCUGAUAAUAGAUGAAUAUAUCAA

>N799

GAAGAUCUCGUUAGCGUGAACCAAGACCAAUCUUCCGCUGAAAAUUAUGAU

>N800

AACAAGUGGCUAGAUCUUUGGCGAGACUUUUUGAACCAAGCAAACUAAAUA

>N801

UUCUGACAUUAAAAAUUGUAGCAAGACGGUCAUUGGAAUCUUUCAAAAAUA

>N802

UCGAUCAGAUCCGCCUAUAUAAAAGACAACGCACCGAAGGUGAACAAGAUC

>N803

UAUACUCAGGAGCCAGCGGGACCAGACUAGAGUGGAUUCCUUGGUAGAGGA

>N804

GGCCACGGCAGCAGUCUGGGUGGAGACGUUCACUCUCGCCGUUGUUCAUGA

>N805

AGUGGCGCCACGGAAGGAUAGCGGGACUUUAUCAAAUCAUUAAUUUCUGUG

>N806

GGUGUAUACAACGAACAUUCAUCGGACGCCUUUUUUACUGCUAUUGUUCAA

>N807

GUGAAGGCCGUUAGAAAGAGAGAGGACCCAUGGAAUGCUAUCAUUGCAGGG

>N808

AACCUCUGCAAGCUUAGGCAUGUAGACAUUAUAUGAGCCAUUUUUUCAUCG

>N809

GAAAUGGGUAGUUAAGAAAAGUCAGACAAAUAACGAUUUAUUCUUCGACAA

>N810

UGUUUUUCUGAAGGACCCGGUAAAGACGAUCCUGGAGGAAACCACUCACCU

>N811

AUUUCAGGAGCACGAUAAAACCUGGACUGGAUGUAUGUAUAAACGGUUCUU

>N812

GUAAGUACUCUCUUGGGGUUUUUGGACGUUUGGUAGGUAAAUUCUGGAGAA

>N813

GCUUGAAAGGGGGUUGUAGUGGGGGACUUGUACUAGGAGGAUAUGCGCUAA

>N814

UGGACGAAUAAUUAGUUGCAGCCAGACUCUGCCUUCUUUGGAAAUCUUGAC

>N815

AUUUACGUCGUCUGUACGCAUCUAGACUUUCGUUACUGUUUAAGUCAUCGU

>N816

UGAUUCUGAUGCUGCUGCUGUGGAGACCUUUGAGAGCCUUGAUUUACGUAC

>N817

UUAUUCAUGUUGCUAUUGGAGCUGGACGAGUCGUUAUUAUUUGAUGAGUUC

>N818

CGGUGAGACAAAAGUAAAGAAAGAGACUUGAAUGGGUUGCUGCAUCUUAAU

>N819

UUUCUACAUCAACCUUUCAAACAAGACGGAGAAGAUUGAAGAAAGUGGAGG

>N820

UUAACAAAUUUCUCCCGAUUUAGAGACCAAGAAACCGUCGGGGCAGUUAUA

>N821

CCAAGCUUAAACAAUAAAAUAUCAGACGAUGAGUUGGAAAGGAUACUAAAG

>N822

UUAGGCCUAUUUAACAGGAAAAAGGACAGGAUUUUAAAUCUCGUCUUGCUC

>N823

UUCGUCAGGUCUCAGCAGUAGGGAGACACCAAUUGAAUGUACAGGUGCGUC

>N824

AUUAUGACAUAUCCCUAACAAAAAGACAAUAAAAGGAUAAACAAUAAUAAA

>N825

GUUAAUAACCAAAGAAACUUGUUGGACAUCGAUACCUCUAGCCAACAAAUC

>N826

UAGAUUUGUUCCUUGAAACCAGAAGACAACAUUUCAUCAGCUUCAUCUAAG

>N827

GGAAGUACCACCGAUACAAGCGUGGACCUUGAUGUCCAUGUGGAAAGCCAA

>N828

AGUACCAGAUUGAGCUUGAGCCAAGACAUCGUGACCUUCAAUAAUAGGCAU

>N829

UCUUCAAUAUCAGUAAUACCUUCAGACAUAUUGCAAUUACUAUUUAUUAGU

>N830

UGGACGCGGUCUAAAUAAUCCGUAGACUCCCAAUCAAAUUUAAAAGCAGAU

>N831

UACCAUGAUUCAACAGGUCUUGAAGACAAGGAGAUUCGUAGUAAAAAUUUG

>N832

AGGCGUAUCCAUUCCAUGAGUGGAGACACCUUAUGCAAAACCUCUUUGCUG

>N833

CAAUAAUGAUAAUCUCUUUAAAAAGACUUCUAAUUAAAGCUAUUUCCUGAG

>N834

UCACCUCUGGAUCUAACGUAACCGGACAAAGCGUAAGUGAUGUAUUCACCU

>N835

ACAUUUCUUAGAGAUGUGUACUUAGACCACACAUGAUAAAGUAUCAGGUAA

>N836

CAGGACCCAGAUUCGCUUCAAACGGACCCACUAGCCAGCUGGUAUGGUUCC

>N837

CCUGCCCACCAGCUCCCGGCGAGAGACGCGGCGAAACAUAAAGCAUUUAUU

>N838

UUGGUUUUUCAAGCCUACGUUUUGGACUCUCAGUCGCUGAUGCGGAUAAGU

>N839

CUCGAACAUUAGGAACAGAGCCAGGACUCUCUCUAACCCCAAUGACUUUCA

>N840

UUGUUUAAACAUGUUAAGCCCGUAGACGAAUGUCCUUGCUUCCAAGAUAGU

>N841

ACAGAAACGUUGCGGCAAGAAAAAGACUGGAGGCCACGCACAAAGAAGGUG

>N842

GGAACAAAAAAAAAAAAAAAAAUAGACAAAAUGUAGUGUAAAUGUAUCCGU

>N843

UAAAAAUUUGAGGCACCGUACCAGGACCCCAAAAGAAGAAUAGGUUAAAGU

>N844

UGGUAGCUAGUGAGGCACUCAAGGGACCAUAUGUCUUGGCAUUUUCUCUAU

>N845

ACCCGCGGAGGCGGCUGAAGAAGAGACGGCGGCGGCUAAUCUAGUACUUUG

>N846

CUUAGGGUUUUGUUUUAGGUCUGAGACAUUUUAGGGUUUUUUUUCAUCAAG

>N847

AAACUGGGCGUGUGUGCUAACCAAGACUGGAUCACCUUUACAUAGCACUUA

>N848

GAUGUUCCCGAUAGAUGCCCUUAAGACGCGAAUACAAUCAGCCAACGCGAA

>N849

UACGCAAACGCACCAUCCUUUUAAGACAGCUAUUAGUGGUGCCUGUGCCAC

>N850

CACAACACCUUUAGACUGCAUAAAGACAGUACUGCAGAUAAGGGGCAGUCA

>N851

CAUGCCGGCUACUGCUAUAUCAUGGACAGCUUAUGAAUGUGCAAAACAUUU

>N852

UUAGUAUACCCAAAGGGUAUUUCGGACGCAGGCAAGCUGCCAUUAUUUAUA

>N853

AUUGAAACUGCUCAUACGUUUUUAGACGUUAUGGGUGAAUUCCUCACCUUA

>N854

UAAUGGAAAUUAUAAGGAAAGCUGGACUUUCAAAGGAGAAGUUGUCCUACG

>N855

CCAAAGAUGCGUGCCUUUCUUCGAGACUUUGUUUCAUGAAGAAAAUACUAA

>N856

CUCGCUGUAAGUGCACCACUUGAAGACACAGUUGGUGCAAUCUAUAUAGUU

>N857

UGCCAUGUACGGUUCAAAGGUCGGGACAUACAAGGCAAGUGAUGUUGACUA

>N858

AAAAAUACUGACAAUCAAGGAUGAGACCACCGAAGAAGCACAUCAGUUACA

>N859

AUAAUAAGCUAUUUGACCAAUUCAGACAAAUAUCAAGUAGUUGAUAUCUCA

>N860

AAAAAUGGCGUCAUUCCAGCAAAGGACAGUGACCACGUUGAGUGGCAUAUA

>N861

CUUAAAACUGGUUCUAAAGAUUAAGACAAAAUCAGAUUCCAUCCCGGAUGA

>N862

AACUAUCUCUUUCAAUGUCUCCAAGACAGCAGCUCUUUUUUGGAAAACUGA

>N863

UUAUCAGUCACCAAAUUCUUGUAAGACUUAUUCAGUACUCCCAGUUCCAUC

>N864

UUCAAGAGCUCAGGAUGAAAACUAGACAAAAGGAACGAGAUUACCUUCAAC

>N865

AGGGCUAUUGAAUCUUUCUUCUUGGACCCUUGUACAAUACAGAACAUCGGU

>N866

AGGGUUUCACCUUUCUUGACAGAAGACACCAAUGGAUUAACAUUUACAAUU

>N867

ACAAGUUCUCUUGGUGGCCUUGAAGACAUCAAUUUUUGUUCCAAAGGCUGU

>N868

CAAAACUAAUGUUUCGCCAUUAUAGACAACACGCUCAAUACCACCGUUCAU

>N869

AGCGGGAUUGUCAUGCAGACGAAGGACGAUAUCAUCAAUGGUUAAUUUACC

>N870

AUGUAAAACCGGAUUCCAAAAAUAGACGUGUGGUCUCCUUCAGUUCAGCUG

>N871

ACCCCUUUGAAACAUAGGAUGCAGGACGACGGAACCUGUUGGCAGAAGGCA

>N872

CUUGUUUAAAACCUCCAGAUAUUGGACGGCAAUACCAUGUUCAGAUAAAAA

>N873

AGUAGAGGCCAUUUCAACACCUAAGACUGGAUCAGCUCCUGCUAAACGUGG

>N874

UUGCAAUGAAUUGGAUGUUGUAUGGACCUGUAAUUUUCAAAGCCUUACCAA

>N875

ACCUGCAUUUUCAACAUGCUCAGAGACAACAUGCAUAACCAAUUCACCAUU

>N876

CGGCACCGGAUAACACAUAAGAUGGACGUACCAAAACUGGAUAACCCACCU

>N877

GAUACGGUAAACACCAGAACCCAAGACCAUAACACCGUGGUCAUCAAAGGA

>N878

AGCAAAAGCAUUUGCAAUGGCGAAGACACGCAUAUCCGUAGGGUUGUUCAA

>N879

UCAAAUUCACCAGCUUGACCGAUGGACAAACCACCAGAACCGAGAACUAGA

>N880

AGGUGUGGAUUCCGGAUGGAAUUGGACGGAAAAAUAAGGUAAUUCAGAAUG

>N881

CACCUGGACCAUUUGAAAUAAACAGACCAUCAUAAUCUUCUUUAGUGAAAU

>N882

GUACAAUUUAGGUUCAUCGAUGGAGACCUUAGAAACUAGGUUUUGAACAUU

>N883

CUGUCAGAGCCGCUUUUUUCCAAAGACAACCUACCCAACAUUGAACCUGCA

>N884

UUCAUCUCUCAAGUGCAUAUCUGGGACACCAUAAUUGCCUACCAAUGGGUA

>N885

CGUCCUUAAGUUCCAACGUAACCAGACGGUCACCCGUAGAUUCCAUUGGAG

>N886

UAAAAAUACAAGACCAUUAGGAAAGACGAUAAUUUGAAAGAAAAUUGGUUU

>N887

CCGAAAAAAAGAAAGAAAUCUUAGGACACCAGAAAUGUACGAAGGCAAAUG

>N888

AACAGAUUGAAACAGGCCGACAAGGACUCUAUAGCCUUUCGAUAAUGAUUU

>N889

AACAAUAUGCCAUCCAACGUAGUAGACAACCAAUAUGGUGCAUAAAAGUUU

>N890

CGUUGUAAGUAGGAUAGUUGUUAAGACCAUUGUGGUCGCUUUCUCCAUUCG

>N891

AUGAUGGGCUUUCCAAUUUUGAUAGACCAUUUUUUGAAAAUCAGGCGUCCU

>N892

AGUUCUGAAUCAGAAUGUGGUUCAGACUGCUGUACUCGUUCAUCUUCAUCG

>N893

CAGGGGACCCGAUGACUAAAGGGGGACCUUCAUCUUCAGCAGUUGCUUCAU

>N894

AAACUUUCGUCUGCAACUGUGUUAGACGAACUACCUACCGUCGAGUAGGAG

>N895

GUUUUAGCUUGUUUCCUAGGAUAAGACGUUCUUUGGGUACCUGUUCUAUUC

>N896

CCGUAAAGAGCGGAAAAACCAUCAGACAAUUCCAGAGCAUUUUUUCUUUCU

>N897

UUUAGGUUCGACAAAAGGAAGUCAGACGAUAUAUAUGUGAAUAUAUAUAUA

>N898

GUCUUCUUUCCGGAAUCUUUUGUAGACAUAAAGAAACAAAAUCUUGUGCGU

>N899

AUUUCCAGAAUGCUUAAACCUAAAGACCAGAUGUCUGACUGCACGGUAUAG

>N900

AUCCAUGUAUUCCAUACACAUGUAGACGGCGCCCUCAAUAAAGAAUGCACC

>N901

CGUAUUUCGAGAAAUUUGCGAAAAGACCACCACCACCACUACUACCAGAAU

>N902

ACGUUCUUAAGAGUGCUCUUGGUGGACGCUUGCACGACUUGGCUACUCAUU

>N903

AUGACUUACCAGUUUUCUCAUGGAGACUGAGGUUAGCAAACUUGUCUUCCA

>N904

GUAUGACGUUGAUGUCUGGAGAAGGACAAGGAAAAAGUAGUUCUGUGAAAC

>N905

GGUAAAUGCAGAUGAAAGGACGAAGACAGAUGCGAUAAGAACAGCGUUUGA

>N906

GACCUUUGCGGAUAACCUAUAUAAGACGGGGUUUCCUUGCUUUGAAAAGUU

>N907

UAUAUAUCACCUCAUUAAGACACAGACGAUCUUGACAGUUGCAGCAGAAAC

>N908

CCAUUUCCUUAAAUCGUUCUCGUGGACGAGAUUAAAAAUAGAAAUGAUGUA

>N909

CAUUUUCCGCAUCGGAAAACCUAAGACAAAUUUUGAUGAGUAUACUUGGUA

>N910

CUUGUCAACCCUGUUCCAAAAGUGGACGCAUCAUUAAAUGUACCUUUUUUC

>N911

UAUCUUCUGUUGUUGUUCCUUUUGGACCAUUUCUAGAGCGGCCAUGACGUU

>N912

CGUUGGCUUCAGCUGUUGUUGCGGGACUAUUCGUGGCGUUCUGGAAUGUGG

>N913

ACCAUACAUGAAAGAAUCUACUGGGACAUCAAAGCCCUUGAGAAUCCCGGC

>N914

CCGUAAAUUAACGGGAAAAAGGAGGACGUAAAUCCUAAUUUCUUUGACCAU

>N915

CUGGUUUGAGGUCAAAUAUGCCUAGACAUUGCUUCUCCCACUGUAUACUUA

>N916

AACGUGACCAUCAUUAUGAAACCAGACAUUUUUGAAUUCUUCUAGUGAUAA

>N917

UAAAGGAGUCAAAAGUUGAUCUGGGACAGCUGCCUCAUUUCUCACUUCUGA

>N918

UGUUUGACGUAGACGUGCCAAAUGGACCGAUAUUGAAAGAAUCAAAAUCUG

>N919

AUGCCCUUAUGGAACCAGAGAAGAGACGAUUUAUUUCAUUGAGGCAAAUAA

>N920

UUCAGGAGUUGGUUGCAGAACAUGGACGAAUGCAAACUUAUUUGAGCCCUU

>N921

UGAUUUCUGUCCUUGCUAUUUGCAGACAUUUUUGGUUAAUUAAUUCGAUUC

>N922

AUGUUAUGUAUUAAGUGUAUCAAAGACCCAUACGGUCAAUGACAUAAUUCU

>N923

UGAGGGAUAGUUAACCAAAUCAUGGACUAGAUGAAUUCGCAGCACCUCUUC

>N924

GAUACCUUUACAGAUCCAGAGGAGGACCACCAGUUUGAAAUAGAAGCAAUU

>N925

UCAGCCUCAGAGUUCUUAUUUUCAGACAUCUUGCCCUGGUAGUAUGUAUGU

>N926

UGCCUUCUAAAAACUCCAUCAACAGACCUAAGCUGACUUCAAAUUUGCACC

>N927

GUUGACGCAAGCGCUAAGCUAGAAGACGAAAUGGAUAUUGAUCUAGAUGGC

>N928

GCACUUACGUAUCUUGUAUAGUAGGACUGGCUCGGUUUAUGUAUAUUAGAA

>N929

AUCUCUAGAACGCAAUUCCUUCGAGACUUCUUCUUUCAUGAAGGAGAUAAC

>N930

GCGCAUGCAGCUUUGAUGCCCUUAGACUUAAUCAACUUAACUAAAUGCAAA

>N931

GAUUAGAAAUGCAGAGAAUAAAUAGACAUGAUACCUCUCUUUUUAUCCUCU

>N932

CACACAGGUUUUACGUGCGGCCAAGACCGUUGUCAAGUGGUUCAUUAUUGU

>N933

UCAAGAGGAAGCGGAGGCGUCGAAGACCACAUCGCAUCGAGCGUCCACUCU

>N934

UGAAUUUGUCCGCGGAUUUCAUCAGACAAGAACAGGCAUUGGCUAAUAAGU

>N935

UCAAUCUGAAUGAUUUGGUGGCAAGACAUGAAACAAAGGAUCCAGUGGUGA

>N936

UAGAAGCAGCGGCAAAUACGGGGAGACCAUGAAACUCUUGAUUGAUGUCUA

>N937

GGAAUUGAGGGUAUCAAAAUACAAGACAUUCUUUUACCGAAAAGAAGAAUG

>N938

ACAAGUAACCAUUUUUACAGUAAAGACCACCAACAGAUUUACCAUCGCUUG

>N939

CAGUAGUAGCCAUCCUUACAUUCGGACGAAGUGUUGGCAUCCAUGUCCAUG

>N940

UCAGAGGUAUCCUUCUCAGAUGAGGACAAUGUAGUGGUAGUAGCAGCAGAU

>N941

CUGUUAGUUGGAUAUCAGUAAUGAGACGAAAAAGCUCGAAAUGAAUGGAUA

>N942

GUUAAUUGUCGCUGUUAUUGUCUAGACUUUUUCUCGGAGAUGGCGCAUCUA

>N943

AGGCAAAUUGAGUUAUGACAAGUAGACAUGAUGCCGCAGCCUUGCCUGACU

>N944

UAUGUUGGGUCCUCUCGUUCUCUAGACCAUGACCAGGAAUUGGAUUCCAUU

>N945

GGAGAAGAAGAGGUGGGCUCUGUAGACAAGAAUGAAGAUGGCAAUGAUAAG

>N946

AAAUGUAUAAAUAAAAAAAUAACAGACAUAAGAUAAAGUCGGGUUAGGGCA

>N947

GGACGAGUCCGGAAUCGAACCGGAGACCUCUCCCAUGCUAAGGGAGCGCGC

>N948

UGAGGAUUCCUAUAUCCUCGAGGAGACCUUCUAGUAUAUUCUGUAUACCUA

>N949

UUCCACUAAGGCUAACUCUCAACAGACAACAACACCUGCUUCAUCAGCUGU

>N950

AUCCAUCUGGUUGGUCAUUUUACGGACACCCAUCUAUGAUUCCGUAUACAC

>N951

ACAUCUAAUAACUCUCCCAGCACGGACAACGAUUCCAUCAGUAAAUCAACU

>N952

AUCAUUCUGAUGAUGAACUCCCUGGACACCUCCUUCUCGAUUCAGGAGCAU

>N953

GGUGACCUACAAUUUCACUUCCAGGACAACACCAAAACAUCAAUAAAGGUA

>N954

GAAACUGAAAUUGAGGUAUCACGAGACACAUGGAAUAGUAAGAAUAUGCGU

>N955

AAAUAUUAUGACAGAAAAGAAAUAGACCCUAAAAGAGUAAUAAACUCAAUG

>N956

UAAGAUACCGAUGAUAUAUAUUCAGACUAUUUCUACGACACAGAAAAACAA

>N957

UUUCACAUCGAUUUUCUCUGGAAAGACGGCGUAAACAAGAGAAGAAAUUAA

>N958

GAUGUAUUCAGGAACAAGUUCACAGACAACAAAACAUGCUUCAGAAUGCUC

>N959

CUCAAACAGAUUGAUGAUAGGUUGGACUUUUUAGAAGAGUACGGAUUGGAG

>N960

CCUUUUAUAGCAAAUCCAAAAAAAGACACAGAAAAAGCUUCUCAACAUUCC

>N961

AAAACAAAAUUGGAGAACUAGCAGGACAUCUUGCUACAAUUAAUUGUAUGC

>N962

CAGGAUAGAACAAUUAGGCAAUGGGACUUACGUUCCGGGAAGUGCUUGCAA

>N963

AUGGUUGAGGGUCGUGAAAAUGGGGACGUAAAUAUUUGGGCCGUAUGAUAG

>N964

AUUGUGGUUCUAAAAGAGGGAACAGACGCAUCUCAAGGUAAAGGUCAAAUC

>N965

UUUAAUUGUGACAUCGAAUCAAAAGACUACUAUUUCUAACGAUGGUGCCAC

>N966

CAUCAACGGUGUCGCCUUUAAGAAGACAUUUUCGUAUGCUGGAUUUGAACA

>N967

GGCAAUUAAUCUUUGAAAAGCUAAGACAAGUGGAAGAAACAGGUGCCAAUA

>N968

UAACUUGAUUUUAUCUGUUGACGAGACGAUCACUAAUAAAGGUAGUGAAAG

>N969

UUAGCAGAUAUUAUAUCUGGGGAAGACAUCGUCGAUCUAAUUUGAUUCGGU

>N970

GAGGCAAUACCAUUAUUGCAAUUGGACACUAUUUCACUUCCGUUAUUUGAU

>N971

AGGUGAGAAUCGCUUCUUUCCUUGGACUCCGUUUUUGGCUUCUUGGAUAUC

>N972

GUUUCCCAUGAAGCUUUAAGAAUAGACCACACGCAUUACAAAGCAUAGCUC

>N973

CUUCCAGCAGCAGGUGUGGAAAAAGACUUGUCAUCCGCCUUAAAAUUCAUA

>N974

AAAUACUUUGUCAUUCAUCUUCAGGACGUAAGAGUUGGAAAUUUCGUGAAC

>N975

UCAUUGGACGUUGCUAUUUCAGUGGACCAAAUUCUGAAUAAAACUUUCAAA

>N976

AUCAAAUAAUUCAAUAGAAGGUGGGACUAUUUUUGGAUAGAACGCAAUUGA

>N977

AGGGUGAGGGCUUGAGUUAAUAUGGACCCCUCAAGCUUCUUACCAUAUUUG

>N978

UCAGAGCCUUCUAACUCGAAUUUGGACCCUAUUACCAAAGUUAGGUGGUAU

>N979

AUUUAAGACACCAUUUGAAAACAAGACACGGGAUUCUAGUGAUUUCUUCUU

>N980

AAUUACAAGUAAUAGUUUGUUUUGGACUUGCGGAGAUGCAGAAUUUCUAAU

>N981

UCUGAUAAUAAGGAAAGUAAAGUGGACAAAAGCUUUUCGGAUCCAACAUUU

>N982

ACUCGAAGAAUCCACGAUAUUUUGGACGAUUUUUAGUUUUUGAGCAUUAUU

>N983

UUGAUUAGCCCAAAACAUCAAAAAGACUUUUUCAUUUCUCUUUGGCGUUAA

>N984

ACGUUCUCUCCGUAAAGUUUAUCAGACAAAAAGUAGUGCUUAGUGGAUGGU

>N985

CUUUCUCCAGUUUCUGUUAGCAUGGACACAUUCGUUGUGUUUAUCAAUUCU

>N986

UUCUUAUCUAAGAAGGUUAAUAUAGACACAGUGUCGAACUUGCUAUCAAAU

>N987

GGUGUUUGGUAGUAGUUGAGAGUGGACAAAAGCAGCAUCUCUGUGUUUUUA

>N988

ACAUUUCUAUCAAGAGAAAUGGAAGACUCUGAAAAGAGUGUUCUUGAGAAA

>N989

UGAGCUAAUUGGUCACUCAACGAAGACAUUUUCAAGGUUAUUCGUAAUUUG

>N990

AUUGUAUUUGCACUUUGCAACCCAGACAACAACGAAUUUUGUGAAGCAAUA

>N991

GACAUAACCGGUGCAAGAAAUAAAGACCAUAACUGGCAGUUGUGAGAUGUG

>N992

UGAACCACGGAGAAAUCAAUUGAGGACAUGAAAUUUCGUUAGUGGCAUUGU

>N993

AUAAAUAAUUGCGUAGAACAUACGGACCGCGCCAGCAACUAAGCUUCGACU

>N994

CUUGAUGCAGCUUCCAAAGGUUUAGACCUUGAGUACACCUAACCAAUUGAA

>N995

CAAAAACUGACCAUCUAUUUCAAGGACUCUAGAAGUCAUAACCAUAUAUUC

>N996

CCCUCAUGAACGUCUACGAUUUUAGACGCAUCAGCUAGAUCGGAUAAGGGA

>N997

UGAAGGUAUUGGUGCCAAGCCAGGGACCAAACCCCCUUGAUUUCCAGUCAG

>N998

UCGCUGGUUUCUGUAGAUCUUGAAGACAAAUCAUUUUCUAGAGCAUCCGUA

>N999

UUCUCGAGGAGUUGAAACAAGCGGGACAACUGCUACGUCAUAAAAAUGGGA

>N1000

UUCUCUCAGUAGCUGUUUCACCAAGACUCCGCACUCAUGGUAAGUAGAAAA

>N1001

AGACUUCGGCGGCAAAAAGCCACAGACAAGUGCUGGUUGCAAAGAGGUAAA

>N1002

UAUUUUUUGGGUAAAGAUAUAAAGGACACUCCAGUCGGUUAAGGUGGCUGU

>N1003

UCUAUCCCUUUGAAGACUUUAUCAGACAGAUAUCAGCUGAUCGAAAAACUA

>N1004

GAAAACACCGUGGCAUCCCAAGUAGACGCAAGAGGCAAUGUGACUAAUACA

>N1005

CUCUUCAACAAAAUUGCCCGCAGAGACAGAGUCUAAUGAUAUUGAUAUCAG

>N1006

UACCCUUUCAGAUUCUAUUGAUGAGACUGAAUUAUCAAAAGAAAUUCGUAA

>N1007

AAUGAACAUGCAGAUAUUGAUGAAGACAUCGUUCUACCAUAUGUGAACAAC

>N1008

CAGUUAUAUAGUUAUAUACACAUAGACAUACAUAUGUUCGCAUAUAUAUUA

>N1009

UGAUGGCUUUAAUUUAACUUAUUAGACAUUACUUUGCGUUUAUUCCCCUCC

>N1010

AAAUUAAAACGGCAUUAAUGCUAGGACAACCAGAACUGUUACUACUGUAUG

>N1011

CGGAUAUUGAUAUGCUAGUAAAAGGACGAGCUCAAGAGCGAAAAUAUAAGU

>N1012

AAUUACUUGUUUGCUUCUCUUUAAGACAAAAGCAUAGAUAAUUUCAGCGUG

>N1013

CAAGGAAGGACAUACACAACCACAGACAAGGUCAUAUCGCGGUCGUCGUCG

>N1014

GCAAUAACAAUAUCAGAGUAUAAGGACAAAUAUGUUAAAAUGUUUAUUGAU

>N1015

CCAACGUGGAACUAGUAACGGUAAGACCGCUUGACGAAAAACCAAGAGGAG

>N1016

GUUCUGGUUGAUGGGGUCAAUAAAGACUAUUUUAACAAAUUGUGAUUGUGC

>N1017

GAAAAGCAUUCGCCGAGGCGUAUAGACAAGCGGCUUCACAAUCAGUGAAAC

>N1018

GAAUCCAAGGGCGAUUUAAACAUGGACAAGAUUAAUAAGAGGUUUAACUAU

>N1019

AAAAAUGCGAAGGCGAAAGCAGGGGACGCUUCAACAGCGAAACCUCCUCCG

>N1020

GAACGUACUGUCAGUUGGUACUAAGACAAGUCCAUCCCCGUAGGCACUACA

>N1021

AAGGGAAGAAGUUUUUGCCAAUCGGACAACACUAGCUCUAUUUAUACUUUU

>N1022

AUAAGCGAUAAAAUCAAAGAAACGGACAGAAUGCAUAUAAAAACUGUAAUC

>N1023

AACAACAGGCACACUUCAAAUAAAGACCGUUACUUAAUAAUAUAUUGCACU

>N1024

UAUAUUAUUGGAAGUAUUUUCUAAGACGGUCUGGCCGUUAUCCGUAUUGAA

>N1025

CAGAAUUCACAAGCUGUCUGGAUAGACUUUCUCGUCUGUUUCAACUCUGAA

>N1026

GCAGGAAUCUCAAAGCAUAUUGGAGACGUCGACACUGGCGAUACGAUAACC

>N1027

CGCUGUUGCCGGCGUAGAGGCACAGACAGAAAAGGUUUGGAAGCAAAGUAA

>N1028

UCGUUGUAGGGAAUCGAUGAUAGAGACUUUGACUGAAUACGACGAAGAUUU

>N1029

UCUUUCAAUUUCUCAAAAUGAUGAGACUGGCCAAACGGUUUUAAACGGUAU

>N1030

CUAUUAUUGCAAGUUGCAUUGUGGGACUUCAAAGAGGCGGAAAAAUAGCUA

>N1031

AAGGCUCAAAUUCUGUCUAUUGAAGACGAAUCAAGUGUAUCAAAUUCUGGC

>N1032

CAAAGAUAAAAAAAACACUCAAGAGACCAGUUCUAAUGUAAAAAAAAUUAU

>N1033

AAACUCGCAACAAUUGUUCUGCAAGACCCCUCAUUUUUUUUUGCUAUAUAA

>N1034

AUCUUCAUAUUUUCCCAUACUUUAGACAUAUGAAUAUAUGCGUUAAUAUUA

>N1035

AUAUCUUGACUUUGGUAUUUCAAGGACUUUAUCUUGUACGUCUUUGGCAAU

>N1036

AAGACAGUCAGCGUCAAAGGGACAGACCUGGAGCCCACAUGCAAGUUAUUU

>N1037

CUCACAUCGUUAGCCUCACAAAGGGACGAAUCCUCCAUAUUAAGCUCAGUG

>N1038

CUUAUGUGUUCAUUGUACGUCCUAGACUCAAACCACUGCAAAGGCGUGCCC

>N1039

UUUCAGCUUCUACAAGUGACUCGAGACCACGUGGAAAGAUCCAACUACUCC

>N1040

CGGCUCACUGAAAAAACCGGGGAGGACGAAAAGGUUUCCAGCCACAGUUGU

>N1041

UAAGAAGUAUAUAUCACUGACAUAGACAUAUAGAUAUACAGCUAGUUUUCU

>N1042

CAUAACCGGGCAUAAAGUGAACUAGACACUUUCAAGAAGCCAACCAAAGCA

>N1043

GUUGGACUGCUUCAUAAUGCAGAGGACAAAGCGUCAGGGCAAGAAGAAGAA

>N1044

GUAUUGAGGCUGUAGAGACACUUGGACGCGAACUCGAGAGAAUCCAAACUG

>N1045

AUAUGGCCCAUUUUCUCCCAAAUGGACCAAAUGGGCACACCGCACGUUUUU

>N1046

UCAAACUUCCAAACAAUAAGUGGAGACUCAAGUUAGCCGCCAUCGACAAUG

>N1047

GUCUCUAUUUUCAAAAACGCCAUAGACGAACCUAUUGGAUCAUAUUCCACU

>N1048

CUCUACUUUAGUAUAUAGGAAAUAGACUCUUUCGAAAAAAACUCUUCCUGU

>N1049

AAGGGUUAUCAAUUCCUCAAUCGAGACAUAUUUAAAAGUUGUCCACGAAUU

>N1050

AUUUAAUUUCUUCUGGCAAUGUUGGACUAGGUCCCAUUGAGAUUGUUCACA

>N1051

AUACGAUGCUGACGAUACUUUCCAGACAACGGCAAUUGACUGUAACAAGGA

>N1052

AGACAUGAUAACUAUUUUAAAUGAGACGCUCGACCCCCUUUUAUCGCUGCU

>N1053

AACACAUCAACCGAACUAGCCUUAGACUCUUUUGAAUCUUGGUACAACCUA

>N1054

UGCCUCGUUUGCGCAAAAAUGAUGGACACCUGGAAACAAUGUACAGUAGGU

>N1055

GAUUUAUUUUUAGAUUUAUAUCAAGACUUGAAAUUGAGCAAGAUUAGCCUA

>N1056

AUCCUUCUUCAACCUUCCAUAGGAGACGAAAUCAUGGUCAUGAUCGAUGCC

>N1057

UUAUAUAACCACUACGGUUGGAUGGACUGAGGGUAAGGGUAACCGUAUACC

>N1058

GCAUCGCUCGAAGUCAUCGAACGGGACUAUUUUUGUUUAAAAUUUCACCAG

>N1059

UGUCAGGGUCCUUUUGGAAGUUUGGACAAGAUUUUGGUUCACAGUCUCCAU

>N1060

UAUGAGCUGCCUAACAGAGAAGAAGACUACAAAGCUUAUAAGCCAAACCUG

>N1061

GAUUUAGUACGUGGUGAAGACAAGGACACAACGGAGGAUUUUGAGAAUGCU

>N1062

CAUCUGCUUUGAUUGAAAACGAAGGACUUCUCGCUAAGCUUUGGUCGAUCC

>N1063

GAACGCCUUUUGGAUAUGAAUAUGGACGGAAUAAUUGAAUUCAUAUUAAAA

>N1064

UGAGAAGAAGAUUCCCCUUUUACAGACGUCCUAUGGAACGAUUGAACCGUU

>N1065

CGUAGUGAAUUAAAAGAAAAUGAAGACGACAAUACAGGUGAUGCUGAUGAU

>N1066

AAUCCAUUUGAACCCCAAUAUUCAGACGUCAUUUUGGAUUCUUCUGAUAUA

>N1067

AUGAAUGCAUAGAAUAUGAGAAAGGACAUGACACCAGGCUUGGAUAUAUGG

>N1068

UACAUUAAUGACAUAAUGCAAAUGGACAAUGUACGGUGUCAAGAGGAGGAA

>N1069

AAAUUUAAUUCAGAGCAUUACAGGGACUAGGAAAUAAUACUAAUUAAAUAA

>N1070

UAUUUCCUAACUCACAAUCGUUUGGACUACAUAUGCUGUCUAGUGCCUUAU

>N1071

UCAUCUCCUUGUACCCAAAAGUUGGACAGCCAGCAUUCUUCUACCAAACUA

>N1072

CCACUGUUGCACAAGUGUCUUCAAGACUACUAGUUGUCCUCGGCAUCUUCC

>N1073

AGCCUACUGAAACAAGUGUGCCGAGACAAUCUUAAGUGUGUUGCUCUAAUU

>N1074

UAGUACAAAAGCUAAGACUACUAAGACAGAUACAACUCCACUAAAAUUAUC

>N1075

GGGAGGUAACGAAGAGAAGAAAUAGACGGGUCCGUCAUGCAAAGAGUAAAG

>N1076

GGCCCCAAUGGCCAUUCAAUUCCAGACCGACGCGUCUUUUAAGUUAUAAUG

>N1077

GCCCUUUUUGAGGAAAAUAGCGGGGACAGCACAUACACAUUCUAGGUCUGA

>N1078

GGAUCCUACAGACCCUGAUGAAUGGACAAUGCAUCGCGUCACCUCAUGGUU

>N1079

UGUUUAUGAAAAGUAUUUGCCGCAGACUAAAACUGCUUCAUAUACCAGGUU

>N1080

CCCAACAAAAUCGGGCCCUUCCAAGACCGAUGAAAAGAAUUUUUUACAUUC

>N1081

CUUCGUAUCCUAGCAUAUUUAGAAGACAUCACAAAAGUAGUUCAUCUGAGU

>N1082

UAUAGUGUAAAGAACUUUUUAUUGGACCAAAAAUUUUAUCCUAUGAAGAAA

>N1083

ACUUCUGGAAAAAUUUAUAUCAAAGACCAAAUGAAGCUUCAACAAAAACCG

>N1084

UCAAGUGCUAAGAAAAGAUGAGGGGACUGAAAUUGAUUUCAAUCAUCGUAG

>N1085

UCCCGCAAAUACUUCUCCUCAGAGGACCUUAUCAACUUCUAAACAGAAUAA

>N1086

AUGCGUAGGUUGAAAACAGACCAGGACUCGACGAGUACUUCCCCAUCUUUG

>N1087

AAAUGGAGAGAAAAAUGACCUUUAGACCAUCUCCGGAAGUCGUUUAUCAAA

>N1088

UCACCAACAUCUCCGAAAUCCUUAGACAGCCUACUUUCACCAAAGAAUGUG

>N1089

GAAAAAACUCUGUAAAACUAAAAAGACAGAACACCAAAAUGUGGGGUACAA

>N1090

UUCGGAAUUUCAAGAAAAUCAAAGGACAUAUACUCUAAUUCGGAUAUGACC

>N1091

UCGCAAAUCGGACGAAGUUUUCUGGACGCAUGCUUCGAGAUAAAUCCAGAG

>N1092

AAACAUUCAAUUUCAAAUCUACCAGACUCGCGAAGUUUAUAAAGUCAAAUG

>N1093

ACAUCAAGCACAGUCUCUAUAAAGGACGGCUUUUCAAUUGCUUCUUCUGGA

>N1094

GUUUGCGUAUUUUUCAUCAAUGAAGACUGGGCUCCCUCUAUACUCAUACGA

>N1095

GUAUCCACAUCGUCAACGCUUUUAGACCAGAUAUGAACCAUCUUUUUUAUU

>N1096

UUAGGCUUGGGAGAUACCCCUAAAGACAGGUAAUUUAGCAGCUUUAAAGAU

>N1097

UCUUGUCCAUACCCUCAGAAUCGAGACCAUCUGCUGCACCAUCAUCCUCUG

>N1098

GCCCGCAUUCAAACCAACAGUUAAGACAACGAUUUCAACGAUACCUUUGCA

>N1099

AUCGUUAAUGAUACCCGCCGCAAGGACAACGAUACCUGCUCUAUCCUUGAU

>N1100

UGUGAAAUAAAGGAAUCGCCAGCAGACAACCAAAACCGAAUGGCACUGCUA

>N1101

UUAAAUUGCAUACCAGUAGUAUGAGACAUGCUUGAAAGAGAAAUAAUGUAA

>N1102

UGGAGCUCAGCCACAUUAAUGCGAGACGCAACGGGUGCAUAAUAUUGAAAG

>N1103

UAAGUGUGUAAUCUUUAUCCGCCAGACAUAUCCGAUGUAGUUUUUUGACGG

>N1104

CUUUGACGCCCAUUAAAAACGAUGGACUUGACUUUAUUAUUGAAUGAUUCA

>N1105

CAUAAACACUAUUAAACCCAUCAAGACAACACCAGAUAGCGAAAAGAUUAA

>N1106

CCCGCACCCCAAAUCAACCAUAAAGACAAUAGUACAGUGUAUGCCAUGAUA

>N1107

AAAUAAUGGUGCAUACCAAAUAUAGACCAGAACUGAUACAGGCAAACCAAA

>N1108

AUAGCAUGAAACCACAAACCAAAAGACGAACAGUGUUGAAGAAGGCUCUGC

>N1109

GUUAUACUGCUAGAAAGGAGUGAAGACUAUGCUCACGAUCUACGAGAUGCG

>N1110

GCUUAAAACAUGCUAGGGUAACGAGACGCGAAUGCGAAAAACUCCCAAAAA

>N1111

AAAAAUAUUUCUCUACACCUUUUAGACAGCUUCCUGUCCCUCUAGUUUCUU

>N1112

CAUUGCCGGUCCCGGCACAGGGAAGACUAAGGUUUUAACUUCAAGAGUAGC

>N1113

GGCCACUUGGUGGAUCUACAGAAAGACUGGAGAAUUAUUGAUGAGAAGGAA

>N1114

GCCGAGUAAAAAUGGAGAUGAAUGGACUAUUCAUCCGAAGCUGAUCAAGAA

>N1115

CAAGAUACUAACGGCAUUCAAUUGGACUUGAUGUUUCUUUUUGCUAAGGGA

>N1116

UUGCUAUCCUAGUAAGACAACGAAGACAAAUUAAAAGGAUCGAAAGUGCAU

>N1117

AUAAGUAGUAAAAAAAUAAUGCUAGACAUACCGACCAAAGGCCGUUCUGUG

>N1118

CCGAACCUAAUUUAUUAAACGCAAGACAUAAGAAUAUUGAACUUUUGAAAA

>N1119

CUGUAACUGUGGAAGAUGUCGAUAGACCAAGAAUUGCAAGCCGAUUUUUGA

>N1120

GUUAGUGAUUUCACGUCAGCUGCGGACCAGUUACGACUCGAAACGCAAAAC

>N1121

AUGAGACCAUCACCCACGAGGAAGGACAAAGUUACUCGUAAUAUUCAUUUC

>N1122

GAGAGAGAUUUUGGGCUCGAAAAAGACGAAAAAAAUAAAACCAAAAUCUCG

>N1123

UUUGGCUGACGCAGCAAUGAAAAAGACACAGAAAUUUUCCAAAAAGGUGAA

>N1124

AGACAUAUUGACUAAACCAGUAGAGACAUUUGCCAACAAAAAUAAAAACAA

>N1125

GAAGUCCAAUAGUCCCAAGUCGAAGACUUCUUUGAUGCUGUAACUACGUCU

>N1126

AAAUGAAUAUCUUAUAAAAUUACGGACUACAUUUCAAGAAUCUAAUUCCUU

>N1127

AGAAACAUCCAUAAUACUCGAUGAGACACUACGCUUUUUUUUUCCAGAAUC

>N1128

UCAUUAUUUUCUCCAGAUGCAGCGGACAAUAAUUUAAAAAUAAAUGCUGGA

>N1129

GUAAAAAGAUCGCUAAAUUCAUUAGACCAUAACGGAUGGGUUUUCGGGAUA

>N1130

UCAUGAUGCAAUAACUUUGAAGAAGACGAAACUUGAAACUCUAUUGUGCUG

>N1131

UGAUCGUAAAUGCCGCUCAAGUAAGACGUCUCAAUUUUACCCCCAUUUUGA

>N1132

UUUGUCUUACUAUGCCGGUGUAGAGACAUUGCCUUGUCCCAUAUCUUUUCA

>N1133

GAGUCCGGCAGUAUAUUUUCUCCAGACAGCCAGAGCUGAUAAAUGUCAUCA

>N1134

AUUGGUGGUGGACCCAACAACAAGGACAUUAACUUGUCUGGUCAAAUCUUU

>N1135

AUACCUGCCAUACUAGAAAUAGAAGACUUAUAUAUCACGUAAAAUUUAUCU

>N1136

UAAGUAACGAAACAAGGUUAUAUGGACUCUUCAAUUAAAGAAGGAAAAGGA

>N1137

AAUGCGAGGUGUGGGUGCAAUCAAGACUCCGAUCCAUUUUCGCCGUUCGAC

>N1138

UUUUUCUUCUCGCUACUCUGAAUAGACGAUGGCCAAGAGGAAAUAUGGCAG

>N1139

UUCAUACUCGAAAAGAAUUCGAAGGACGCUCACAAGAAAGAGGUUUUAGGC

>N1140

CGUGUCUCAACGGGUCCUGCUUAGGACCCCCAACUUCACCAGUCUUGGGGU

>N1141

GUCUCUAAAACACUUUAAUAAAAGGACAUCAAUUGACGGUGCGUACCUUGA

>N1142

UUGUUUGAAUGAGAAUGUCCUGUAGACAGUGAAGAUUGUAAACUGUUCACA

>N1143

UUGGAAGAAAAUAAACUAUCAUUAGACGUGGUGUUCCCCUCCAUUCGAUUA

>N1144

CCAUUGCUUGACUCUGUAGUUUAAGACUCGCCUAGCCACUUAUGCUUAUUG

>N1145

GGCUGGAACCCGAUCUGCUGAAAAGACUUUUAAGUUGGUAUACAGAAGAAU

>N1146

UAGGAGCGGGCACUGGUUUAGUAGGACUUUCAUGGGCCUUAAAAUGGAAAG

>N1147

CUCACGAUUUUAUAGAUAAAUUUGGACAUGAAAAUGAAUUUGACGUCAUAU

>N1148

AUCGAAGUUUUUGGCGGCAUCAGGGACCUGUCACCUAGAAAUACCUUUAAG

>N1149

GCCAAAAGGUUAGUUUUUGAUUCAGACAGGAAUCAAUACUGGUAGUACAUU

>N1150

UUGUUCUAAGUACGGUAGUCUUAAGACAAUCUCAGCACUAAACUUGUUUCU

>N1151

ACACCUCUGCGAAUAACUGUAAAAGACAAUUUGUAGAAAAGGCCCUGACAU

>N1152

AAAAUAUGAUAUCCUUGAACUCAGGACCGCUGAUGAAUUGAAACUCAAUAG

>N1153

UUUAACAUCCAUAUCUGGAAGGGAGACAACAGUCACAAGAUUCAAAGAACA

>N1154

AACAAAUUGGAAAACAUUUGGCGAGACAGAACGUGACGUAGUAAGGUUGGU

>N1155

UCUAGUGGUUUAAGUAAUAAUGCAGACCUUGGAGUUAGUUCUAGUGUCUCU

>N1156

GCCAUCAGUCAUCCUGAUUUGCAAGACGCAUUUGCUGCUGUGCAGUUUUUG

>N1157

GCCAAACCUGGAUUGGUUUCCCUGGACGAACUCUCACCAGCCUUUUUGUCU

>N1158

AUGUUCCGCUAAAGUAUCUAAUGAGACAUCAUCUCCUUUAAACCAACUUUU

>N1159

GUUCUAAGCAUCCUAAUGUUUUGAGACUUAUCGAUUGUAAUGUCUCUAAAG

>N1160

GUAGAAUGUGGAGUUGCUCACAGAGACAUCAAGCCUGAAAACAUCUUACUC

>N1161

AAAAUGAUGGAAAUUUAAACUGGGGACCCUGGUCAAAGAUAGAAUUUACUC

>N1162

AGGUAUGACACAAGAGGCUAAGUGGACGCAAUUCAUAAGCUAUGAUAUCGC

>N1163

UAAUUCCCAACUGAAAAAAAUGGAGACAAAAGAAGGGUUAAGAUGCACUGA

>N1164

CAGCAACUUCGGGUUGUUCUUCUAGACCUGUCUCUUUAGAUACCGCAUGUU

>N1165

CGUUGUUUUCACCCUCAUUAUUAGGACGGGCUUUCUUCACGCUCUUGUACU

>N1166

AAGCGGUUUAGUGAGAUAUAAUAAGACUAAUUCACGGAGCAUAUUUGUACU

>N1167

UCAGUAAUUUUAACAGUAGCUCUGGACUUAUUUUGAAAAGAACUUCGCUGG

>N1168

UAUUACGCCUCAAAAAUGGGAGCAGACCGGAAAACAGUUCAGGAUUCCCUG

>N1169

AUAAAAUUAUUGUUCGACCACAUAGACACAUGAAGUAAUGUAAACAAAUUA

>N1170

UAUUUUGCGUGACUUUUAAUAUAAGACUGCUAAAGGAUUCUAAAUGAGUUU

>N1171

GUAAUAAUUCUCCAAACUUUGAUGGACAUUAAGCGAGUCUUGGGGUGAUUA

>N1172

AAAUUUUUCGUCUCUCAAGAAAGAGACCUGUGCUGUUAUGAUUAUCUUAUU

>N1173

AGCACUGAAGAAUCGAACUAUUUGGACAUAUAGUCUAAUUACAAAUGGAUU

>N1174

UGUUUAUUCUCGCAAUCACUUGAAGACGCUAGCUUUCGCCUCUUUGGCGUU

>N1175

CCAUUGGAAUAAUUAUCAUGUUGAGACCUACGGAGGAUAUGCUGAUCAAUU

>N1176

ACGAAUUGCCUCAAAUCGCUCUAAGACUGCGUUAUUACUUUCGAUCUUCUU

>N1177

UAUACAAACUGUUGCAGAUCUGGAGACUCCGACCAUACAAUGCACCAGAAC

>N1178

GGGCCCUUCAAAUUAACGAAAUUAGACUCAAGAGGAAUCUUCAUAAAAAAU

>N1179

AUUCUGCGGACUAAACUCCCGCAGGACAGGUUGUAAAUGGCCUAAAAUUUG

>N1180

CAAACAUUCAAAAGAAUUUCCACAGACUGAAUGUUUAAGUUCCCCAAACCU

>N1181

GGAUUGUCCGUAUCAUAAAUUAGAGACUUUAAAUUUACUAUGCUUGUAUUC

>N1182

CCACUCCGUUUUAGCGUGGUAUUAGACCCAACCUUGUUUCUAGAAUACCUU

>N1183

UGAAAGGAAGUGCGAACUUUCCAGGACUGGAAGAUAUCAUUCACUUCGAGA

>N1184

AGCACAACUGAAUUCGAAAGGAAAGACAAGCCUACGAUUAAGCCUAAAUUC

>N1185

GGUGUAGAGGAAGAAGAAGAAGAAGACGAGGUAUCCGAAGAAGAAGAGCCC

>N1186

AGAUUCCAGUAUAAAUCCGCACUAGACGUAGGAGAACAUUCCGACUCUUCU

>N1187

ACCUUGAACUUACUAUUUUAAAAGGACAUCUCGAAAAAGCUUAGGGAUAAU

>N1188

AUUAUUGAUACGUUGUUAAUUAUAGACAGAAAUGUAUUCAGUACAAUAGAA

>N1189

CCCGAGUCUCCGGAGACGAAUGCAGACUCCUCCCAACUAAUCAAUUCACUA

>N1190

AUAUAUCAAAGGUUACCUUCUUGAGACGCACCGAAUAUGUCUCCAAUACAA

>N1191

CUAUUGCCCGACACAGCCUCAAUGGACCAGGUGUAUUUCAUCCUAAAAUUC

>N1192

GAUGAAGACGAGGAGCAACCAGAAGACGUUAAGAAAGAAUCAGAAGGCGAU

>N1193

AUAACAGUGAGUAUCGGUGAAGAGGACUAAUGAAGCAAAACAGGCGUUAAU

>N1194

UCCAUUAACAUCAUUCGUAUUCGGGACAAUCUCGUAUUUUGCAAAAGCAAA

>N1195

UAUUGAAGGCAUUGUAGUUCUAGGGACACCACUAUGGCUCCCUCCGGGAGA

>N1196

UAUAAUCAUGCUGUGAACCACUGGGACCUGGUCCUUUCAACCCUCCAGAAA

>N1197

UAUUGACACCAUAUUUGAACCGCGGACUUGGCCCGUUUAGUGAAGUGGCGA

>N1198

UUAUAAUUGAGAGUAUAUCCUUUGGACAAUAAAACUAUACUCCAAAAAAGG

>N1199

UAGCAAUACCUUCAAAAUAAUACGGACAUUAAAUACAAAUUAACUAAUUAU

>N1200

UUAGACAAAAUCUCAGUCAAAUUAGACCAAUUUUUGAUUCCGAGAUUUUCG

>N1201

CCGCUAAACUUGACUGUUGUCAUAGACAAGGAAGGCUUAGUCCAGUUGGUA

>N1202

AUGUCCAUCAGGCUUAUCGCUCCAGACUUUUAUUUGGGCAUUAAUAACACC

>N1203

GUAUUCCAGUUGAAAGUAUUUCCAGACGAUAUAACGUCAUAAUGCCCAUAC

>N1204

GUAUUAGUAGUGUCUUUGCUUUGAGACACUGUCUGGAAAGAAAUAAGUUCU

>N1205

UUUCCAAUAACUGAUUAAACCUGGGACACAAUUUAUUAAUAGAAUAAAUAA

>N1206

AUGAAUUUGAACUAUAUCCAGUCGGACCCAACGAAUCAAAGAACUUAUCAU

>N1207

UAAGCUUGCAUUUUGACAGCCAAAGACAAUAGUUUCCAACGAAUCCAAAUA

>N1208

UCUCUAAUAGUCUUUUCGCCAAUGGACCAAAUUCUCACCAAAGAAUCUGCG

>N1209

ACAGAGCCUAAUACUUGCGGGAAGGACUUUCAAUGUUGCUUCACUGCAGCU

>N1210

CUCACCCAUUUCUCAAUUUAGUAAGACCUCUAUUAAUGCUCUUACCAGGCC

>N1211

GGACAAGAGCUUCAGGGAUUGGUAGACAUACAAAAUUCGGUGGUUAUGUGA

>N1212

CAAAAUCAAAUAAAACAUGGCUUAGACAUUGCACCAUAUGUCAUCUUUCUU

>N1213

UUACAUAGCACCUGGAACACCCAAGACUUGAUUCAAAGCACUUUGACCGCC

>N1214

ACCGAUAACGGUGAUCAAACUUAAGACGGAUGCACCCGUAACAGCUGCGAU

>N1215

CAAAAGAGAUAAUGGGAAGGUUGGGACUGCUAAAAGAUUAUUAGCAUUCUC

>N1216

UGAUCUGAUUGGCAAUUCUACACGGACACUUUGCAAAUAGCACACAAUAAU

>N1217

ACGAUAUCAAUUAGACGAAAACCAGACAUACUAAAACAAUGGAGAGUGGCG

>N1218

GAAGGACCAAAUGACAUCUAUCAAGACUGUGGUAGCUAGCGAACAAAGGAU

>N1219

AGACAGAGGAAUGAUUAAACCUAAGACGAGGAUAAUCCUUAUGCAUUAUGA

>N1220

UAGUAAAUAGUACAGGAAAAGGAGGACUUAUCAGGGUAUCACACAAACAAU

>N1221

GAUCAGGGUUUAGCUUUCGACGGGGACGUAGGAAUUACAUCACAAGCUCGA

>N1222

UUUGAGCAAACACAUUUCCGCAUAGACCGCAAAUGCCUGUUGGAAAUGUCU

>N1223

ACUUACAUUCCAAGGAAGAAAUGAGACAGAGCAAAAAGGUGCCACACAAGG

>N1224

AUUAUUCAAAGUGAAUUUCGAGGAGACCGGUCAGUUCUUCAAUGGGUUGAA

>N1225

CUGCAUUACGAUAAUUCCGAUGUGGACAAUUGGUGGAUGGUCUUUGCCAAU

>N1226

AGUUAGUUGCUUGCCAAAAGAUUGGACAUCUGGCGGUGAUAAUCCAACCAU

>N1227

UAGAGCAUAACGUUCCGAUCAUCAGACGUCAAUAUCGCAGUAGCACUCUCG

>N1228

CCCACUGACGAUAGCAUGGAGGCGGACAUUUCAGAUAGAGAAAUGGCUACA

>N1229

AGCUACUUGAGAUACAAGAAAUAAGACAAUUGAGCUGCUUCAUUAUAUAUC

>N1230

UUGAAUGCUUUCGAUGACAUGCAGGACUACUAUGAUGUUUCUUUGCAAGAG

>N1231

AUUACGCCUCUGUAGUCCCACCAAGACACAACGAAAAGCAAAUUGCCCUUA

>N1232

ACAACGAACCUAAAUCUAAAGAAGGACUACACGGUACAUUAGGCGAACCUA

>N1233

AUUGUUGCCCUUGGUGCUCAUUCAGACUCUGUUGAGGAGGGCCCAGGUAUC

>N1234

AACUCCAAGAUCAGAGUAUUUAUGGACUAUGACAUGAUGGCUUCUCCAAAC

>N1235

GAAGAGUUGAAAAACCUGUACGUAGACUACUACAAGGCUCAUCACUUGAAC

>N1236

GAUGAUGUCUCUAACUUAUCCUGGGACGCAUUCAUUACCAACACCAAGUUG

>N1237

GAAACCCAAAAGCACAAAGAGGUGGACAUAUUGAAUGCUCAACAACCACAA

>N1238

AAAAUCAUUUCUCUGUUGAACGUGGACCUUUUUACACCAUGUCUUAUUUUU

>N1239

GGAAAGAUAAUGAGCCGUAUCCUAGACUUGGAUAAAGAUGAAACUAAUUUU

>N1240

ACUUGCUGAUCUUUCAACAAAUCGGACAAAUGCUGAGGUGGAGCUGGGGUU

>N1241

UCAAGUGCAAUCUUUGUUGGAAAGGACGCCCAACAUUGAUAACGAGGAACU

>N1242

UCCUUCUGCGGAAGUCUUCCCUAAGACGGUUCACCAUAGCAAGUUAUUGAU

>N1243

AAGUACAUAAACGUGAGUAUUUUGGACGAUCCAAUUUUCCUUGUUGUCGGU

>N1244

UUUUAUAUUCUUUUUUUAUUCCAAGACUUAUAUUCUCAUUAUAUUCUAUUU

>N1245

UCCUAUGUACGAAUAUUCUAAGGAGACUGUUUGUAAAUCAACAUUUUUUGU

>N1246

CCCAAUCCAUUUUGGAAGUGUAAAGACAUCAAGCCAAUAGAAUUCAUCCUU

>N1247

UCUUCGAGCAAUUGAGGACAUGUGGACUGAACGCGGGUCCACAGGUGCUUG

>N1248

AACGCCAAAUUGAGCAGCAAAGGGGACAGCAAACGGCACAAACUCAGCUAG

>N1249

UUCCUCAAGUUAGAUCCAUGAGUGGACAACCUCCCACCAAUGUUCAGCCAA

>N1250

CUACCAUAUCCAACCUAUUGGUCAGACAAAAAAGCAGAUACGGAUACUUUG

>N1251

UUAGUAAUAAAGAGUAUAUUAGUAGACUGUGGCAUACACUGAAGUAUUAUC

>N1252

CAGUUAAUCGAUCAAUUUGAGUGGGACAUCUCUAAUAGUGAUAACUGUCCA

>N1253

AAAGAAGACAAGGUAGAUCUAAUAGACGUGGUAUGCUCGCAUUGUCCGGCA

>N1254

UGCAGAUAUUCCAAGAACUUUCAGGACUCCAGUACCAAGCACUUUAAUGCC

>N1255

CCACUUAUAAAAGCAGGCCAGAUAGACCUAAGCCAGUUUCACCUCCUUGUU

>N1256

GAGACUGAAGGUAAUAACGAACAAGACAUACCACCUACAUACGACGAAGCU

>N1257

AGUGAAAACAAUACCACCACUCAAGACCAAUCUCCCAAGUCUAGGCGUAGC

>N1258

AGUAUCAAGGGGCAUGGUGGAAUAGACCGUUACAAUACCACUAAAUGCACC

>N1259

AAGACCAGAAAAUCCUUUAUCACGGACUAAUGAUGAAUAGUUUCGUACUAC

>N1260

CGGUGUUACCAAUAAUAAAUGCGGGACAUCCGACAUAAAUGGAACCAAUGC

>N1261

ACGUCACUUUUGGUAGCUUUACUGGACAUUGGUAAAAUGCUAAUUUAUAAU

>N1262

UCCAUAUUCUUGACUCUUCAGUAAGACUAACUGAAUGGAAAUAGAGAAAGU

>N1263

CAGCAAGCUUAGUAUCAAUAGGGAGACCCUGUCAUGUAAAAAAAUAAAUGA

>N1264

ACUGUAUAAGUCGAUAUUUUAAGGGACGCUAAAUAUAUCAUCAUAGCUCGU

>N1265

AAACAGAAUAUAAAAGAAAAUAGGGACGACGUACCAUGGCAUGAAGAAUUG

>N1266

AGUGAAGACUUAGAUGAGGACUUGGACAAAGGCGAAGAUAUAGCGCUUUCA

>N1267

ACACUUUCUACAAUCGUAAAUUCAGACGUUAUUCCAAGUUCGAAAAGAGGA

>N1268

GUGUUCUACUUCUAAAUUAUCAUGGACAAGCCCUUCUGUGCUACUGCUAUU

>N1269

UAUUUUUGAUACCUGCAAAUCUAGGACAAGGUAUCACUUUUCCUACGACCU

>N1270

GCCUUGAUACUCUGCAUCCUUAAAGACAAUCUGGCGAAACCUAAAACAAGA

>N1271

UGCACUGUGAAAAAAAGAAACAAAGACCAAUAUAAAUAGUGAAGUAAAAUG

>N1272

CUGAGGCGGCAGAAAACAGAGACGGACUUCACAAUGGUGAUGAAGAAAAUU

>N1273

UCUAAAAAAAUAUCCCGAAGACCAGACAGAAGUUACAGCUCCUAUCAUUGC

>N1274

AAACAUUUACCAAACACAAAGUUAGACGCCGUUUUUGGCUUGAUUCCGUUG

>N1275

GAAAUCAACCAAAGGUAGCAAAUAGACUGAAAUCCUUCUAUUUCUAUGCAC

>N1276

GAGGUGGGAGUUAUGAAAAUCCCAGACGGUCUGCUAUCUAAUAUGAGUUCA

>N1277

GAUUGGUGUGACAAACUUGAUAGGGACAUUUUUUCACUCAUAUCCAGCAAC

>N1278

CAAAACCACCUGGACCUUCUGGAAGACCAACCCGUUAGAUUGUAUUUCAUU

>N1279

CUUAUAACCUUAAGGAAAAAGUCAGACCGUCCAUGGAAUGAUCCUGGUGAA

>N1280

AAGCUGUGAAUAGGUAUGCGGAUAGACAAGUCGAAUUCCAUUUUGCCGGAA

>N1281

GAUAUACCCGAUUUUUCAAAAUGGGACGUUUAGUGUAGUAGAUAGUAAGUA

>N1282

UUCAGUAGUUUAAGGCGCUGAAAAGACAUGGUCUAGAAGUUAACUUGAGCU

>N1283

UGGUUUUUAGGCGGAAAGAGUUUGGACGACUUGUCUCUCAUAAUGUGGAAG

>N1284

UGCGGCGAUGCUCAAACCGGUGGAGACCACACCAAUGAAUCCUGUGUUGAU

>N1285

UGCAUCUCGUAGCUGUGAAAAGAGGACUUUUAAAGGUACCACCAAUGUUGG

>N1286

UUAACAAAGAAAAAGACCUAUGGAGACAAAGAAAGGUUGAGCAAUGACAAU

>N1287

GUGCUGUUACCUCAACUAUUUCUGGACACUCUUCGAGUGAAAUUUCAAGAA

>N1288

CUAUUAUCCAAUUUGUUAUUGCAGGACCGUUUUACUUAAAUGCCUUGAAGA

>N1289

GUAAAGAGACGGAAAUUGAUAUUAGACUUCUCCAAUAUGGCGAUAUUUUUA

>N1290

UCGUGUCAUGUCCCUGCGCGAUUGGACUUGCCGUUCCUAUCGUAUUUGUUA

>N1291

GUCACUGCAGUUUAUGCAUUAGAGGACUCUUUACGGGCAGAUGCUGUCUCC

>N1292

GCGAUAGUUCUUCGCAGUCAAAAAGACCGGUUGUUGUUUUUUGCGGUGACG

>N1293

ACGUCAUUAGCUCCAUGAGCAAAAGACAUAGUGGCUGCAGUAAUGGCUUGG

>N1294

CAGUGGACUUAAAGUAAAAAGAUGGACCUCUGAAAAUAUCAAUCAACUUCA

>N1295

UCUUUCCAAAGACUUAACUUCAAGGACAGAAAACCUAGAAAUUGAAAAAAC

>N1296

GCAGUAGCAAAUGUUAACCAACAAGACGAGCCAAUCAAAGCACUGGUCAUA

>N1297

AGACAGCACCCAAGAACUCACAGAGACCCGCCAAAACCAUGGCUUGCCAGU

>N1298

UUGUUUGGAGAUGUCCACCCAGUAGACAUUGUAAAAAAAAUCUGCUAUUUA

>N1299

UAUCUCUAUCAAAAGAGAUUAUAAGACCCAUCGGCAAAACUCGAAAUAUCG

>N1300

AUCGAUACAUUUAGUAUACGGGAAGACAACGUAGUGUUGAGAUUCAAAGAG

>N1301

CUGGCAGGUAAAUGGGUAGAAUGGGACUGAUUGGAACAGCGCAAGUUUUUU

>N1302

AUAGAUAAAGCUGUUGAGAGGCAAGACAAUGUUUUCAAGAUGAUCAGAAAA

>N1303

ACUAGUUUAUGGACUCUGAUAUAAGACAGAGUUGACAAGGAAAUGGUGCCG

>N1304

UAUGAUGACCUUCACAAGCUUUUGGACGAAAAAUACAAUGACCAUUACGUU

>N1305

CUUCUGAUGUAAGAAAUUCAUACAGACUUUGUCGUGAAGCUAUUGGCCUUG

>N1306

CCACCUCAGCCAGAGUUCGUAACGGACCCUCGACUUUCUUUGGAUAGCUUU

>N1307

UCUAGUCGAGGUUCCUGCACCGAAGACUCUCUAAAAAGGAUAUGGAACGAA
